# Supplementary material for: Evidence for a non-canonical JAK/STAT signaling pathway in the synthesis of the brain’s major ion channels and neurotransmitter receptors
Source: BMC Genomics. 2019 Aug 28;20:677. doi: 10.1186/s12864-019-6033-2 (PMC6712773; doi:10.1186/s12864-019-6033-2)
Supplement: Supplementary file 1 — Table S1. Genes of interest in the dataset and their relationship to epilepsy. Several genes of particular interest to the field of epilepsy are listed with the degree of FC from DMSO+Water vs. DMSO+BDNF and the FC of DMSO+BDNF vs. WP1066 + BDNF. Many have not been associated with the JAK/STAT pathway or BDNF regulation. The name of the gene, the related function, and a reference to support the potential relationship with epilepsy is listed. (PDF 196 kb) [file 12864_2019_6033_MOESM1_ESM.pdf]

A

Relationship of Epilepsy Genes with JAK/STAT Inhibition					
Gene of Interest	FC V+B	FC WP+B	Name	Function	References
GALR1	34.36	-115.70	Galinin receptor 1	Protective in Epilepsy	Mazarati, 2004
BHLHE22	-5.1	7.78	Basic Helix Loop Helix E 22	Repressor involved in neurodevelopment	Joshi, 2008
SOCS3	2.89	-3.29	Suppressor of Cytokine Signaling 3	Inhibits JAK signaling, increased in epilepsy	Pernot, 2011
DRD5	14.91	-63.92	Dopamine Receptor 5	Knockout protects from seizures	O'Sullivan, 2008
INHBA	5.26	-5.27	Inhibin Beta A	Reduces excitotoxicity	Lau, 2015
NPTX1	5.01	-6.30	Neuronal Pentraxin 1	Plays role in epileptogenic synaptic remodeling	Sanabria, 2008
GRIN2A	2.18	-8.80	Glutamate Ionotropic Receptor NMDA Type Subunit 2A	Mutations cause epilepsy, involved in LTP	Carvill, 2013
CXCR4	-1.53	1.77	CXC Motif Chemokine Receptor 4	Inhibition protects against epilepsy	Song, 2016
HRH3	-3.93	6.53	Histamine H3 Receptor	Increased Histamine confers protection in epilepsy	Bhowmik,2012
RGS4	4.46	-2.77	Regulator of G protein signaling 4	Inhibition protects against epilepsy	Chen, 2012
SH2D3C	-11.46	14.91	SH2 Domain Containing 3C	Involved in cell migration	Wang, 2012
ELAVL4	-2.04	2.76	ELAV Like RNA Binding Protein 4	Controls mRNA processing, increased in Epilepsy models	Bronicki, 2013
FYN	1.35	NA	FYN Proto-Oncogene, Src Family Tyrosine Kinase	Overactivity leads to epilepsy, activates mTORC1 pathway	Kojima, 1996; Wang, 2015
RPS6K(A2, A3, A5, A6, B1, B2)	A2--2.04	A2-2.59	Ribosomal Protein S6 Kinase	mTOR activated, involved in proliferation and cell growth	Cho, 2011
ARC	33.24	-15.04	Activity Regulated Cytoskeleton Associated Protein	Known to be seizure and BDNF induced, plasticity	Kulpers, 2016
BDNF	3.23	-2.90	Brain Derived Neurotrophic Factor	Increased in epilepsy, neuroplasticity	Binder, 2001
JAK2	1.79	-2.11	Janus Kinase 2	Activated by BDNF and Status Epil.	Lund, 2008
EGR3	5.57	-6.08	Early Growth Response 3	Regulates α4 subunit $GABA_A R$	Roberts et al, 2006
