# Supplementary material for: Evidence for a non-canonical JAK/STAT signaling pathway in the synthesis of the brain’s major ion channels and neurotransmitter receptors
Source: BMC Genomics. 2019 Aug 28;20:677. doi: 10.1186/s12864-019-6033-2 (PMC6712773; doi:10.1186/s12864-019-6033-2)
Supplement: Supplementary file 2 — Table S2. Top Neurological disease relationships determined by IPA. (A) Top 10 significantly enriched neurological diseases in the BDNF transcriptome with their p-value (IPA). (B) Top 10 significantly enriched neurological diseases in the WP/RX2 vs. BDNF set of differentially expressed genes listed with their p-value from IPA. (PDF 180 kb) [file 12864_2019_6033_MOESM2_ESM.pdf]

A

TOP 10 Neurological diseases: V+B vs. CTRL	
**Disease**	**P-value**
Seizure disorder	3.29E-27
Seizures	6.75E-27
Epileptic seizure	7.44E-26
Epilepsy	2.91E-24
Neurological signs	1.55E-16
Dyskinesia	1.57E-14
Motor dysfunction or movement disorder	3.75E-14
Abnormal morphology of nervous system	5.14E-14
Movement Disorders	6.48E-14
Huntington's Disease	1.17E-12

B

TOP 10 Neurological diseases: WP+B/RX2+B reversal of BDNF	
**Disease**	**P-value**
Epileptic seizure	7.32E-23
Seizures	3.82E-22
Epilepsy	7.57E-22
Seizure disorder	1.24E-21
Neurological signs	8.91E-13
Motor dysfunction or movement disorder	2.12E-11
Movement Disorders	5.01E-11
Dyskinesia	1.67E-10
Huntington's Disease	6.53E-10
Abnormal morphology of nervous system	9.99E-10
