# Supplementary figures and images for: Evidence for a non-canonical JAK/STAT signaling pathway in the synthesis of the brain’s major ion channels and neurotransmitter receptors
Source: BMC Genomics. 2019 Aug 28;20:677. doi: 10.1186/s12864-019-6033-2 (PMC6712773; doi:10.1186/s12864-019-6033-2)

A

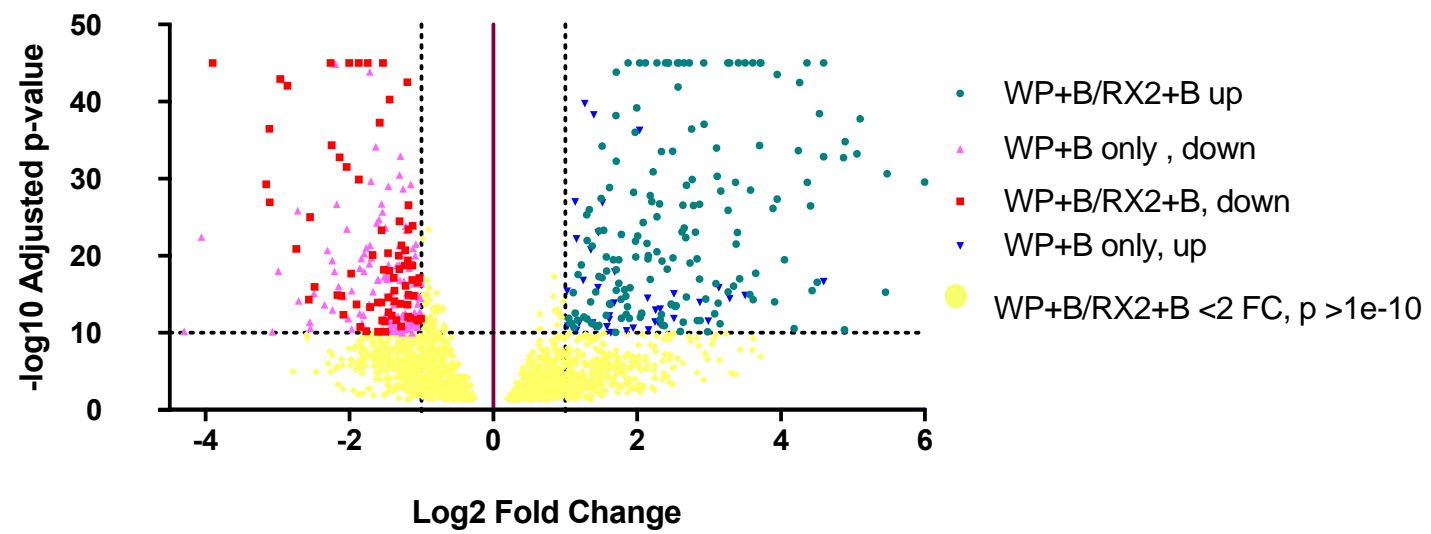

Supplement: Supplementary file 3 — Figure S1. Volcano Plot of BDNF regulated genes whose response is reversed by WP + B/RX2 + B. Representation of degree of fold change and significance used to narrow down the list of top genes to include in the Fig. 3 network. Teal-genes upregulated by both WP + B and RX2 + B; Blue: genes upregulated by WP + B only; Red: genes downregulated by both WP + B and RX2 + B; Pink: genes downregulated only by WP + B; Yellow: genes that did not meet cutoffs of > 2 FC, p < 1e-10. Teal and Red gene list used for Fig. 3 network. Maximum –log10 p value detected was 45, p values more significant than 45 are represented as –log10 of 45. (PDF 103 kb) [file 12864_2019_6033_MOESM3_ESM.pdf]
