# Supplementary material for: Evidence for a non-canonical JAK/STAT signaling pathway in the synthesis of the brain’s major ion channels and neurotransmitter receptors
Source: BMC Genomics. 2019 Aug 28;20:677. doi: 10.1186/s12864-019-6033-2 (PMC6712773; doi:10.1186/s12864-019-6033-2)
Supplement: Supplementary file 4 — Figure S2. Enrichment Analysis of BDNF regulated genes whose expression is reversed exclusively by 10uM Ruxo. Enrichment analysis was performed on the set of 417 DEGs between RX2 + B vs. V + B that were not overlapping with WP + B. (A) KEGG pathway analysis as generated in EnrichR ranked and colored by p value. (B) Top gene clusters involved in the KEGG canonical pathways from A. (C-E) Neurological diseases significantly associated with the RX2 exclusive gene set for (C) Epilepsy, (D) Huntington’s disease, (E) Neurodegeneration. (PDF 279 kb) [file 12864_2019_6033_MOESM4_ESM.pdf]

A

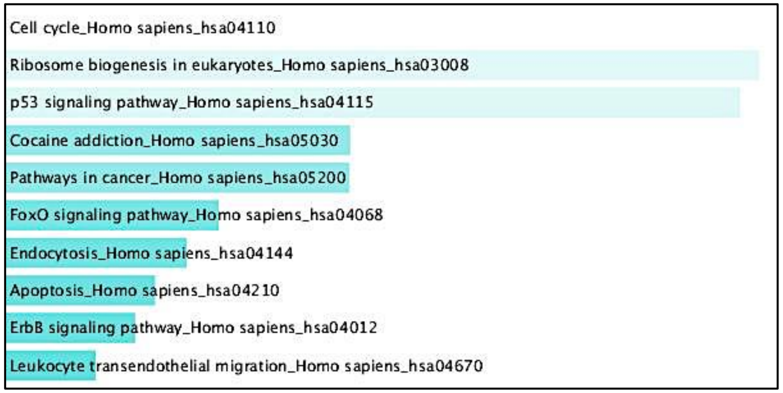

B

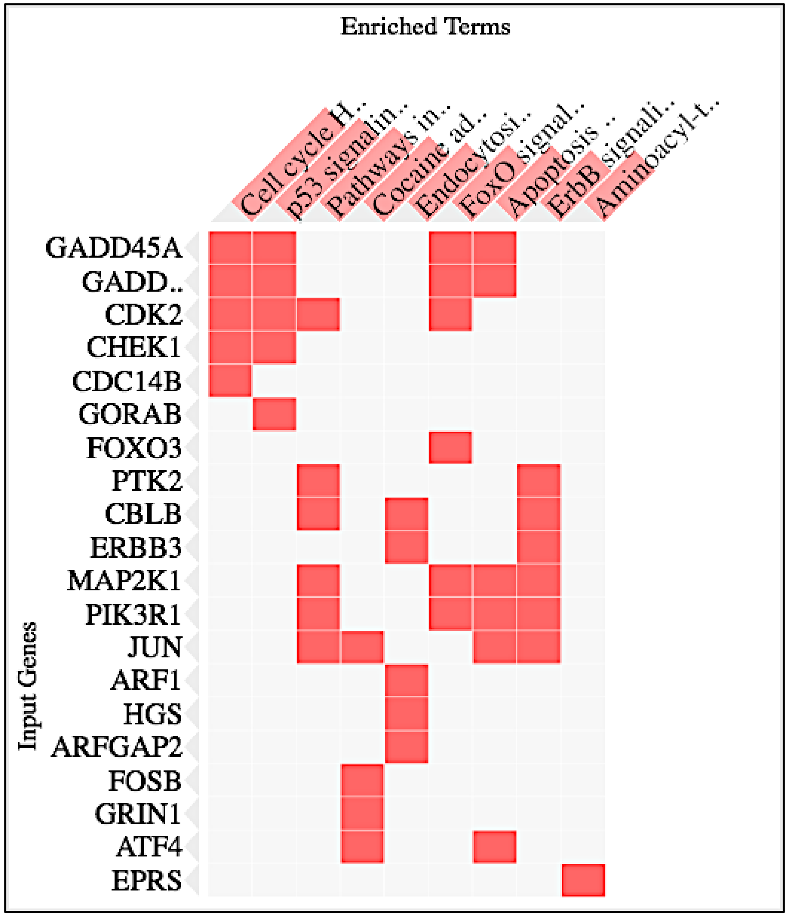

C

Epilepsy	
GENE	FC
EGR4	8.40
EGR1	7.14
RAG1	7.09
PNOC	4.08
MMP9	3.88
GFRA2	3.56
FOSB	3.51
DUSP2	3.06
IER2	2.94
SLC2A1	2.09
JUN	1.93
PNKP	1.83
ADCYAP1	1.79
GADD45G	1.67
PLK3	1.64
ADARB1	1.63
GADD45A	1.47
GRIN1	1.42
PPP1R1B	1.34
RAPGEF3	1.27
MCM6	-0.85
FKBP1A	-0.84
ST8SIA4	-0.70
AKAP5	-0.69
LDLR	-0.67
GABRA1	-0.54
ARRDC3	-0.49
MBNL2	-0.49

D

Huntington's Disease	
GENE	FC
EGR4	8.40
EGR1	7.14
DDIT3	2.75
MLF1	2.13
HDAC5	2.02
JUN	1.93
NGEF	1.81
USP13	1.76
ICAM5	1.65
PRKX	1.49
GADD45A	1.47
PCDH7	1.45
GRIN1	1.42
CHAF1B	1.40
NDRG1	1.38
PPP1R1B	1.34
FBL	1.27
ATP6V1B2	1.26
ENPP5	-0.67
LDLR	-0.67
MAP3K5	-0.65
UGCG	-0.57
HMGCR	-0.57
GABRA1	-0.54
FOXO3	-0.49
MBNL2	-0.49
HBP1	-0.41
ST8SIA2	-0.39

E

Neurodegeneration	
Gene	FC
EGR1	7.14
CRHR1	3.40
SERPINE1	3.01
DDIT3	2.75
CXCL12	2.38
RASSF5	2.28
SLC2A1	2.09
JUN	1.93
NCS1	1.89
ADCYAP1	1.79
HSF1	1.72
GRIN1	1.42
MAP2K1	1.41
PAWR	1.33
RAPGEF3	1.27
ATF4	-1.24
CDK2	-1.41
CDC25A	-1.47
MAP3K5	-1.54
HDAC9	-1.86
FOXO3	-2.03
