# Supplementary material for: Evidence for a non-canonical JAK/STAT signaling pathway in the synthesis of the brain’s major ion channels and neurotransmitter receptors
Source: BMC Genomics. 2019 Aug 28;20:677. doi: 10.1186/s12864-019-6033-2 (PMC6712773; doi:10.1186/s12864-019-6033-2)
Supplement: Supplementary file 5 — List of significant genes and FC values for all relevant comparisons. Comprehensive list of genes that were significantly different when comparing various treatments (PDF 493 kb) [file 12864_2019_6033_MOESM5_ESM.pdf]

V+B v CTRL LIST

Gene Symbol	log2FC_db_dw	pAdj_db_dw	Aliases	Entrez ID
SH2D3C	-3.5186608	0	Nsp3	362111
TXNIP	-2.0627642	0	Vdup1	117514
KLF11	-2.0083106	0	Tcfcp2l2	313994
CCNG2	-1.8418735	0		29157
HECTD2	1.6576949	0		309514
BAIAP2	1.6914603	0	Irsp53	117542
ARHGEF3	1.8163428	0		290541
TPBG	1.8295311	0	5T4|WAIF1	83684
PDP1	1.8499978	0	Ppm2c	54705
THRB	1.9880408	0	C-erba-beta|ERBA2|Nr1a2|R	24831
NEFL	2.0243213	0	NF-L|Nfl	83613
SRXN1	2.0964212	0	Ab2-390|Npn3	296271
SDC1	2.1574545	0	HSPG|SYNDECA|Synd1|Synd	25216
RGS4	2.158761	0		29480
BAG3	2.1794496	0		293524
SPRY4	2.1836648	0		291610
SMTN	2.207883	0		289734
MAS1	2.4278078	0	Mgra|c-mas	25153
GFRA1	2.4593477	0		25454
DUSP6	2.5314097	0	Mkp3	116663
SCN1B	2.5327137	0		29686
KLF10	2.5528164	0	Tieg	81813
GYPC	2.6156454	0	GPC	364837
SMPDL3B	2.759265	0		362619
PDGFB	2.8178334	0	SIS|c-sis	24628
DUSP5	2.8205106	0	Cpg21	171109
GADD45B	2.8589942	0		299626
SCG2	2.8881552	0	Chcg	24765
LPAR1	2.9599757	0	Edg2	116744
GRASP	2.962261	0		192254
HAUS8	2.999679	0	Ny-sar-48|RGD1311565	290626
EGR1	3.0695915	0	Krox-24|NGFI-A|Ngf1|Ngfi|zi	24330
ECEL1	3.1382005	0	Dine	60417
LAMP5	3.2535648	0	BAD-LAMP|LAMP-5|RGD130	362220
GPR158	3.32403	0		291352
RASL11A	3.382681	0		304268
STX11	3.3982918	0		292483
TRPC6	3.5656304	0	Trrp6	89823
NPTX2	3.5869105	0	NP-II|NP2|Narp	288475
ANKRD33B	3.6229866	0	RGD1564227	310200
FOSB	3.7941096	0	fra-2	100360880

CYP26B1	3.9069061	0		312495
EGR2	4.0791073	0	Krox20	114090
FAM150B	4.0822096	0		679566
VGF	4.137181	0		29461
EMP1	4.1743793	0	CL-20|EMP-1|ENP1MR|TMP	25314
HSF2BP	4.194514	0	RGD1565879	499413
SIM1	4.295678	0		309888
RRAD	4.5104423	0		83521
PLAUR	4.754548	0	Par|Plaur3|uPAR|uPAR-2|uP	50692
ARC	5.054995	0	rg3.1	54323
GALR1	5.1025863	0	Galnr1	50577
MMP3	5.109894	0		171045
MMP13	5.2357397	0		171052
GPRC5A	5.4281096	0	Rai3	312790
CITED2	2.067604	1.40E-45	Mrg1	114490
PRSS23	2.4334733	2.80E-45		308807
CCKBR	2.7934568	2.80E-45	Cck2r|Cholrec	25706
CSRNP1	2.6077116	3.20E-44	Axud1	363165
GALNT9	1.649546	7.40E-44		304571
PENK	3.258971	7.60E-44	Enk|Penk-rs|Penk1|Penk2	29237
SPRR1A	4.405632	1.18E-43	Spr|Spr1a|Sprr|Sprr1|Sprr1a	499660
NPTX1	2.3235111	1.19E-43		266777
SSTR3	3.0217788	1.51E-43	SSTR|Smstr28	171044
HOMER1	1.6634928	5.16E-43	HOMER1F|Vesl-1	29546
NAB2	1.9878603	6.96E-43		314910
ALOXE3	2.4174693	2.52E-42	e-LOX-3|eLOX-3	287424
SERTAD1	2.2239814	2.65E-42		361526
GPR68	2.5399456	2.66E-41		314386
EGR4	3.1501546	4.21E-41	Egr4l1|NGFI-C	25129
LBH	1.3661401	6.01E-41		683626
DAAM1	-1.1606426	7.91E-41		314212
RT1-S3	2.45826	5.13E-40	H2-T23|RT-BM1|RT1-BM1|R	294228
SSTR4	2.0445876	6.55E-40	Smstr4	25555
PCDH8	1.7111181	8.03E-40	Arcadlin	64865
PTAFR	3.4609513	1.20E-39		58949
PLCL1	1.8863832	2.60E-39		84587
DHRS9	3.8141215	4.01E-38	Rdhl	170635
SLCO2A1	3.8286128	1.77E-37	Matr1|Slc21a2	24546
KLF9	1.8472241	4.20E-36	Bteb|Bteb1	117560
VASP	1.8119922	7.63E-36		361517
ISM1	3.1125116	8.86E-36	RGD1562551	311760
OSBP2	1.3585519	9.21E-36	RGD1309479	305475
MEIS2	1.2149252	1.08E-35		311311

MYT1L	-1.253483	1.69E-35	Nzf-1|Nzf1|Nztf1	116668
FAM110C	3.6874802	2.14E-35		500638
MAN1A1	2.551768	2.79E-35	Aa2-166|Man1a	294410
IRS2	1.6023843	3.76E-35	4PS|IRS-2	29376
SPRY2	1.3205673	4.32E-35		306141
SPHK1	3.0671668	6.52E-35		170897
AREG	3.6564286	1.21E-34		29183
RDH10	1.7078578	1.30E-34		353252
GCNT1	3.2552552	2.13E-34	core 2 GlcNAc-T	64043
MYCL	-2.4887106	6.92E-34	Lmyc1|Mycl1	298506
DUSP4	2.4186413	1.97E-33	Mkp-2|Mkp2	60587
INHBA	2.3958855	2.04E-33		29200
ADCY8	1.7645378	4.52E-33	Ac8	29241
KCNJ4	1.2723438	5.24E-33	Hirk2	116649
PDXK	2.2978668	1.10E-32		83578
SCN2A	-1.0416238	4.14E-32	NachII|Nav1.2|RII/RIIA|RNSC	24766
LMNA	1.6316702	4.87E-32		60374
PRIMA1	3.1429088	9.42E-32	PRiMA|Prima1l	690195
KCNA1	2.5377295	1.93E-31	Kcna|Kcpvd|Kv1.1	24520
IFRD1	1.5897458	3.00E-31	Pc4	29596
IER5	1.8107331	4.45E-31		498256
TRIB2	1.0827541	5.40E-31	RGD1564451	313974
SMAGP	2.7230294	5.71E-31		300236
DRD5	3.8985677	7.07E-31	D1B	25195
IL6R	2.301657	1.60E-30	IL6R1|Il6ra	24499
FAM84A	1.5672331	1.86E-30	RGD1305779	313969
INSC	3.1004944	1.88E-30	RGD1311379	293166
CYP27B1	2.0879796	3.07E-30	Cyp40	114700
GAL	3.638903	4.39E-30	Galn	29141
ITGA6	1.8318036	4.59E-30		114517
PIK3IP1	-1.2761761	5.60E-30	RGD1311203|zgc:66482	305472
NPL	1.8066884	9.61E-30		304860
HS3ST5	1.6394793	1.43E-29		294449
ACAN	2.3603435	1.89E-29	Agc|Agc1	58968
FST	-2.2953715	3.23E-29	FOL1|FS|Fst-288|RATFOL1	24373
FRMD6	1.591484	3.23E-29		257646
CRHBP	2.8745425	1.69E-28	Crfbp	29625
GEM	2.114901	2.35E-28		297902
FOS	1.8276407	3.46E-28	c-fos	314322
FGFR1	1.739113	1.00E-27		79114
NRN1	2.0853286	1.11E-27	Nrn	83834
EHD4	1.4695604	1.22E-27	Past2	192204
C1QL3	1.7291608	1.40E-27		680404

PRICKLE1	1.6093031	2.63E-27	Rilp	315259
MTUS2	-1.7557187	1.02E-26	RGD1562533	498136
ADORA1	1.7060324	1.44E-26		29290
C1QL2	3.2518566	1.46E-26		288979
LEXM	2.7456307	1.92E-26	RGD1559493	500516
BHLHE22	-2.3506196	2.87E-26	Bhlhb5	365748
RGS16	1.710074	3.55E-26		360857
CXXC5	1.1755618	4.92E-26		291670
ARID5A	1.7327574	7.22E-26		316327
PTPRR	-1.9995065	1.02E-25	PC12-PTP1|Pcptp1|R-PTP-R	94202
ADRA1D	2.8663747	1.21E-25	Adrd1	29413
SEMA3A	2.0394833	1.24E-25		29751
DUSP14	1.8628569	1.29E-25	Dusp14l2	360580
BHLHE40	1.5580747	1.70E-25	Bhlhb2|Dec1|SHARP-2|Sharp	79431
TBX21	3.676611	2.03E-25		303496
SP7	3.4122097	4.62E-25	Osx	300260
TMEM150C	-1.1294942	5.69E-25	RGD1306105	360916
PLCXD2	-1.1535157	7.99E-25	RGD1563504	363781
HTR1B	2.5193267	8.85E-25		25075
CCND1	1.4953775	1.45E-24		58919
PTGS2	2.4685276	2.83E-24	COX-2|Cox2	29527
TMEM65	1.164343	6.48E-24	RGD1563224	500874
RASSF8	2.6404998	6.48E-24	RGD1308820	312846
MTMR12	1.8788508	1.05E-23	Pip3ap	310155
CNGA4	3.2149022	1.55E-23	Cgn2|rOCNC2	85258
RTN4RL1	1.703219	3.08E-23	Ngrh2	303311
MMP10	3.6014867	3.49E-23		117061
MMP9	3.1567497	4.16E-23		81687
FAM102B	1.0435205	5.39E-23	RGD1310037	365903
RNF217	1.0695094	7.63E-23	Ibrdc1	292188
ZYX	1.2675476	1.46E-22		114636
TRIB1	1.8991559	2.54E-22	Gig2	78969
GPAT3	1.9999765	2.57E-22	Agpat9	305166
MYO1E	1.7415842	3.58E-22	MYR5|Myr3	25484
SERPINB2	3.5841072	5.20E-22	Pai2a	60325
DBNDD1	-1.8708739	1.08E-21	RGD1310008	361437
B3GNT7	-1.2929933	1.67E-21		316583
SORBS2	1.1322527	1.91E-21	Argbp2	114901
DOK5	1.6272763	3.00E-21	RGD1562846	502694
HSPH1	1.0666467	4.02E-21	Hsp105	288444
ATG16L1	1.066054	5.70E-21	Apg16l|Wdr30	363278
DLGAP2	2.4980152	6.56E-21	DAP-2|SAPAP2	116681
DCLK3	-2.5603716	7.79E-21	Dcamkl3|RGD1309232	316023

RUNX2	2.8620102	2.15E-20	Cbfa1|OSF-2	367218
HMGA1	1.9424802	2.52E-20	Hmgi|Hmgiy	117062
ZC2HC1A	-1.018401	2.60E-20	Fam164a|RGD1311970	310244
CAV1	1.5572658	2.61E-20	Cav	25404
EFR3A	1.0692867	2.68E-20	RGD1305976	362923
RGS20	1.2998077	3.26E-20		362477
PTH2R	2.567532	4.37E-20	Pthr2	81753
KCNV1	1.3596368	4.89E-20	Kv8.1	60326
GPR83	2.9704585	8.92E-20	Gir	140595
ERRFI1	1.1447444	9.01E-20	RGD1307599|Ralt|Xxx|gene	313729
IER5L	1.5424821	1.11E-19		499772
NEDD9	1.1884135	1.48E-19		291044
WDR43	0.78759044	1.99E-19		362703
BAALC	1.0558892	2.48E-19		140720
DKK2	3.33558	3.13E-19	Dkk4	295445
MAP2K6	-1.2294734	3.97E-19	Mkk6	114495
AP1S3	1.6560899	4.60E-19		367304
PLK2	1.2168903	4.68E-19	Snk	83722
FAM212B	1.5589373	5.27E-19	RGD1306526	310764
FAM196A	1.2841877	5.33E-19		100233213
DNAJB5	1.018804	6.05E-19		313811
SELE	2.8674822	6.41E-19		25544
42805	1.4264649	6.69E-19	MARCH-XI|RGD1559945	499558
VIPR1	1.814615	9.38E-19	PACAP-R-2|PACAP-R2|RATVA	24875
PTPRU	0.9018883	9.45E-19		116680
GCGR	2.2198486	9.56E-19		24953
BAZ1A	1.8905354	1.21E-18		314126
KITLG	1.0291016	1.52E-18	Kitl|Mgf|SCF	60427
POU4F3	2.8643954	3.79E-18		364855
SS18	0.89652956	4.13E-18		361295
ARHGAP10	1.32641	4.25E-18		688429
RGD1307461	-1.6390955	6.60E-18		300990
SYT10	2.6374457	7.16E-18		60567
ATP2B1	0.7975083	7.42E-18	Pmca1a|Pmca1b|Pmca1c	29598
EGR3	2.477239	1.16E-17		25148
TMOD1	1.247792	1.25E-17	E-Tmod|Tmod	25566
BDNF	1.6924942	1.46E-17		24225
CDKN1A	1.5740019	1.68E-17	Cip1|UV96|Waf1	114851
PCSK1	1.3431313	1.73E-17	BDP|PC1|PC3	25204
AJAP1	1.2560861	2.15E-17	neumo48	687031
PPP1R18	-0.9783594	2.15E-17	Kiaa1949|RGD1309543	361790
SHC4	2.0069253	4.60E-17	RGD1309182	679845
KLHDC8A	-1.9683065	5.18E-17	RGD1305132	305096

CARS	0.8850533	6.37E-17		293638
PDE9A	1.5808959	9.09E-17		191569
VEGFA	1.445269	9.35E-17	VEGF-A|VEGF164|VPF|Vegf	83785
CSPG4	1.0248827	1.00E-16	Ng2	81651
HACE1	1.0541575	1.18E-16		361866
ELOVL7	2.0444527	1.19E-16		361895
DNAJB4	-0.92410535	1.29E-16		295549
ADRA1B	1.8079491	1.36E-16		24173
RBMS1	1.556421	2.05E-16		362138
MYBPH	1.4676771	3.00E-16		83708
TRIM9	1.334747	4.48E-16		155812
PIK3CD	-1.2090521	4.90E-16		366508
RGD1563365	1.0237044	4.90E-16		299700
VCL	1.0615209	7.73E-16		305679
PNMA2	-0.8894666	8.17E-16		305977
WDR1	0.78475046	8.63E-16		360950
PLEKHH2	1.9632562	8.70E-16	RGD1304935	313866
CCPG1	-0.99297786	9.71E-16		363098
CREM	1.5828385	1.21E-15	Icer	25620
PRAGMIN	1.5849367	1.32E-15	RGD1311793	306506
SIK1	1.9205624	1.63E-15	Sik|Snf1lk	59329
KCNK2	-1.6440685	1.99E-15	Trek-1|rTREK1d	170899
MAP2K3	1.8152273	2.12E-15	Mek3|Mkk3	303200
UBASH3B	1.6640985	2.29E-15	RGD1310357|Sts-1	315579
SIAH3	2.1123347	3.02E-15		692004
FAM184B	-1.8618681	3.59E-15	RGD1564950	289671
RBM12	1.0843652	3.59E-15		652928
PVR	1.6094185	3.95E-15	Taa1|Tage4	25066
ADCK3	-1.4241498	4.09E-15	Cabc1	360887
HSPA4L	1.0953513	4.81E-15	APG-1|OSP94	294993
NGF	2.332491	5.61E-15	Ngfb|beta-NGF	310738
AGTR1A	1.9648544	5.98E-15	AT1|AT1A|AT1R|Agtr1	24180
SLC6A17	1.475906	6.02E-15	Ntt4	613226
EIF2AK3	0.8770304	6.95E-15	PEK	29702
CDC37L1	0.7691417	7.22E-15	Cdc37l	293886
TIMP1	1.1849104	7.35E-15	TIMP-1|Timp	116510
PANX1	1.0717888	8.85E-15	px1	315435
FAM43A	1.5121964	9.13E-15	RGD1304790	288031
ACVR1	0.99055094	1.10E-14		79558
DDHD1	0.7036751	1.13E-14		305816
FGF2	2.3530617	1.22E-14	Fgf-2|bFGF	54250
GREM1	1.2579631	1.38E-14	Cktsf1b1|drm	50566
ZMIZ1	1.3394367	1.43E-14	Rai17	361103

FGF9	1.2275751	1.46E-14		25444
KLF14	2.2850194	1.77E-14		312203
RGD1305014	0.9552032	2.23E-14		309029
RTN4RL2	1.2922189	2.29E-14	Ngrh1	311169
ETV5	1.6104954	2.42E-14		303828
ST7	0.9344929	2.79E-14		296911
ACTN1	1.5171064	2.86E-14		81634
KLHDC8B	-0.99473333	3.35E-14		306589
GABRG2	-1.2529383	4.04E-14		29709
HIC1	2.4993727	4.96E-14		303310
ERCC1	1.079485	4.97E-14		292673
MUSK	2.7192237	5.00E-14	Nsk1	81725
SYPL2	2.1900775	5.78E-14	Mg29	362018
BAI1	1.1931818	6.54E-14	Bai1	362931
YPEL5	-0.77750516	7.20E-14		298792
JUN	0.8752457	7.57E-14		24516
RRAGD	-1.0506474	8.18E-14		297960
PTPRN	1.0244571	8.32E-14	ICA512|Ia-2	116660
LRRC26	2.4362385	9.51E-14	RGD1308398	311803
ACSL4	0.82943016	1.20E-13	Acs4|Facl4	113976
SCRT2	1.8648645	1.30E-13	RGD1564796	366229
RPS6KA5	-0.8596208	1.54E-13	Msk1	314384
NARF	-0.8929865	1.71E-13	RGD1310894	360681
ANO4	1.349041	1.84E-13	Tmem16d	299714
RAI14	0.94001484	1.88E-13		294804
TRMT6	0.92291427	2.07E-13	RGD1308877	311441
MYH4	2.5091531	2.37E-13		360543
PPHLN1	0.81124485	2.77E-13		366975
UOX	2.5953753	3.03E-13	UOX-2|Uri|Uri2	114768
MTSS1	-0.67216885	3.26E-13		362918
CACNG3	1.1780035	3.64E-13		140724
FAM46A	2.0539212	3.71E-13	RGD1311381	300870
HRH3	-1.9751954	4.26E-13		85268
FKBP5	0.8295817	4.26E-13		361810
SYNJ2	1.9054892	4.92E-13		84018
DLC1	2.12959	6.61E-13	Arhgap7|RhoGAP	58834
EHBP1L1	-1.0739992	7.41E-13		309169
TMTC4	-1.059587	8.35E-13	RGD1560183	290501
SSTR2	0.9105852	9.80E-13	Smstr2	54305
UNG	0.9744515	1.04E-12		304577
GPR37	-1.4318073	1.08E-12	Ednrbl	117549
STAM	0.6097905	1.26E-12	RGD1564499	498798
JUNB	1.1255476	1.26E-12		24517

NUB1	-0.65661305	1.29E-12	BS4|NYREN18	296731
IGSF9	-1.2876827	1.34E-12		304982
CALCA	2.0663934	1.53E-12	CAL6|CGRP|Cal1|Calc|RATC/	24241
TTPAL	1.0955765	1.56E-12	RGD1305754	296349
SIK3	1.1485419	1.63E-12		684112
NEFM	1.3457621	1.64E-12	Nef3|Nfm	24588
DMP1	2.437485	2.08E-12	DENTMAT	25312
PNMAL1	-1.2880445	2.17E-12	RGD1310803	361515
LYVE1	2.454409	2.17E-12	Xlkd1	293186
MRM1	0.79004884	2.17E-12	RGD1566232	363661
LYRM9	-1.2623076	3.30E-12	RGD1562012	497962
CDC42EP4	-0.9025045	3.30E-12		303653
RNF152	-1.9679434	3.43E-12		293561
SLCO3A1	1.2057765	3.58E-12	Slc21a11	140915
MICAL2	0.9951284	4.08E-12	MICAL-2|RGD1311773	365352
EPHA4	-0.9142841	4.79E-12	RGD1560587	316539
HDAC11	-0.90699375	4.79E-12		297453
RGD1305587	-1.0728447	6.45E-12		294499
DPF3	1.2705431	6.45E-12		299186
SPRED3	1.4192659	6.57E-12	RGD1311713	308478
SYNPO	1.3086188	6.83E-12		60324
ZFP143	1.0058349	6.95E-12	STAF|Znf143	361627
KCNF1	1.4807354	6.95E-12	Kh1|Kv5.1	298908
HTR1F	2.439059	7.05E-12	Htr1eb	60448
SESN1	-0.86736435	7.19E-12		294518
EZR	1.0318053	7.50E-12	Vil2	54319
ARVCF	-0.6820622	9.00E-12		303798
EFNA4	-1.4177372	1.06E-11		310643
P4HB	0.5871119	1.10E-11	PDI|PDIR	25506
IFFO2	1.2169538	1.11E-11		641315
VSTM2B	1.2963742	1.25E-11	RGD1306235	361560
KCNC4	1.3986031	1.34E-11		684516
RPS6KA2	-1.0282438	1.41E-11		117269
RGD1561849	1.280649	1.50E-11		500393
VWA1	-1.4944415	1.56E-11	RGD1311476	298683
MCM3	0.88786876	1.83E-11		316273
RGD1304810	1.324045	1.96E-11		306504
WSCD1	0.8512739	2.08E-11	RGD1308212	287466
TSPAN5	0.97393215	2.12E-11	Tm4sf9|Tspan-5	362048
MYC	0.7217986	2.57E-11	RNCMYC|c-myc|mMyc	24577
FLT1	2.370946	2.83E-11	FLT-1|VEGFR-1	54251
NPY1R	-0.9346991	2.91E-11	NPY-1	29358
CLMP	-1.2245033	3.02E-11	ACAM|Asam|Ol16	286939

GPR12	-1.6918236	3.45E-11	Gpcr12	80840
AK1	-0.9593049	3.91E-11	Ak 1	24183
SEMA6C	-1.6502132	3.95E-11		29744
CXCL12	1.1677482	4.43E-11	Sdf1	24772
HECA	0.6286029	4.48E-11		308624
SLC22A3	-1.5044653	4.74E-11		29504
EDNRA	1.3215874	4.97E-11	ET-A|ET-AR|Endor|Eta|RGD1	24326
PCED1B	-1.3029708	5.22E-11	Fam113b|RGD1561028	315283
GPR137C	-1.3275257	5.98E-11	RGD1563070	305812
PIK3C3	-0.86102486	6.10E-11		65052
FILIP1	-1.8423249	6.32E-11	Filip	246776
IPPK	1.1657133	6.91E-11	C9orf12|RGD1311271|rIpk1	306808
SLC9A2	1.4313039	7.30E-11	Nhe2	24783
HSH2D	2.2285504	7.33E-11		100360518
ABHD13	0.65878487	7.49E-11	RGD1308317	306630
KLHL29	0.87309074	7.65E-11	Kbtbd9	298867
ARMCX6	-1.0612724	9.60E-11		363496
CYLD	-0.87643355	9.95E-11	LRRGT00003|Rp1|Rp1h	312937
STAC2	1.1889721	9.95E-11		363674
DIXDC1	-0.8126806	1.38E-10	Ccd1	363062
GUCY1B3	-0.68394923	1.40E-10	Gucy1b1|SGC	25202
NGEF	0.7739159	1.50E-10	Besh3	246217
SLC4A11	1.8949499	1.59E-10		311423
LOC680663	1.8478743	1.69E-10		680663
RUNX1	2.1376946	1.70E-10	Aml1|B|CBF-alpha-2|Cbfa2|I	50662
ZFP238	-1.0958638	1.71E-10	Rp58|Zbtb18|Znf238	64619
SLC43A2	-0.6407087	1.72E-10	RGD1305819	287532
BCAR3	1.0566084	1.86E-10		310838
RND1	-1.0471507	1.98E-10		362993
PHEX	1.9048415	2.01E-10	PEX	25512
UNC5B	1.3878748	2.11E-10	Unc5h2	60630
GRWD1	0.9444853	2.14E-10		308592
LHFPL2	0.89294094	2.19E-10		294643
CDK17	0.75188833	2.35E-10	Pctaire2|Pctk2	314743
MCHR1	1.4962466	2.37E-10	Gpr24|Mch-1r|Slc1	83567
CBLN2	1.2851559	2.71E-10		291388
UAP1	0.9331793	2.85E-10	RGD1561967	498272
HES6	-0.73470825	3.25E-10		316626
SDCBP	0.6194749	3.28E-10	TACIP18|mda-9	83841
CYGB	-1.2168483	3.42E-10	Staap|Stap	170520
ME1	0.57811105	3.42E-10	MOD1	24552
TMEM246	-0.8830453	3.44E-10	RGD1307218	362518
ELMOD1	-0.77389467	3.91E-10	RGD1564186	315670

EML4	0.7334703	4.16E-10		313861
NRF1	0.98583704	5.41E-10	Nfe2l1_retired	312195
RBM24	-1.4161272	5.43E-10		690139
ZBTB46	1.3315436	5.43E-10	Btbd4	311718
POU4F2	2.4603977	5.44E-10	Brn3b	171355
SMAD3	1.0452014	6.06E-10	Madh3|Smad 3|mad3	25631
CREBL2	-0.84521633	7.53E-10		362453
TACR3	1.8273549	7.79E-10	Nkr|Nmkr|Tac3r	24808
LIMK2	-0.8737091	8.14E-10	Limk2b|Link2	29524
RAG1	2.31254	8.26E-10		84600
NDUFAF4	1.0272276	8.27E-10	Hrpap20	362495
KCNN1	-1.3946551	9.01E-10	KCa2.1	54261
KRAS	1.1000634	9.01E-10	Kras2|c-Ki-ras|p21	24525
FMNL1	1.1394138	9.39E-10		287746
MAL2	-0.8264061	1.04E-09		362911
PRSS12	-0.833376	1.55E-09	Nt	85266
HBEGF	1.0555133	1.62E-09	Dtr|GFHB|Hb-egf|Hegfl	25433
RASSF7	-1.6510895	1.65E-09	RGD1306244	293623
SLC25A37	1.4660352	1.76E-09	Mscp|RGD1359361	306000
LRRC61	-0.8664268	1.81E-09	RGD1561800	500111
SLITRK4	-0.9375122	2.01E-09		302473
RAB3D	-1.1076238	2.07E-09		140665
PSME3	0.65283084	2.24E-09	Ab2-371	287716
GALNT7	1.2785124	2.26E-09		29750
LRRC71	-0.78167814	2.38E-09	RGD1309453	310689
CCDC92	0.75157523	2.62E-09		100036765
ZC4H2	-0.76520795	3.37E-09	RGD1561708	367838
DHX57	-0.58144784	3.59E-09		366532
MCM2	0.8999621	3.80E-09		312538
SLC41A2	1.037418	3.80E-09		362861
AOC3	1.9076623	3.96E-09	SSAO	29473
SLC2A3	1.2184542	4.09E-09	GLUT3	25551
NRIP1	-0.7432924	4.11E-09	RIP140	304157
CLDND1	0.586019	4.17E-09		288182
BLES03	-0.8337398	4.38E-09		266609
RASGEF1C	-0.9259993	4.41E-09		360519
TAGAP	1.5805598	4.44E-09		308097
PRKAR2A	1.4116378	4.84E-09		29699
SPNS2	1.150435	4.93E-09		100270678
MCM6	0.58436936	5.17E-09	Mcmd6	29685
TMEM218	-0.89298445	5.23E-09	RGD1311364	300516
ADCY7	1.4964327	5.23E-09		84420
CFLAR	1.031613	5.25E-09	Flip	117279

ATP1B1	0.7771755	5.34E-09	ATPBS	25650
CCDC86	0.79873097	5.44E-09		293738
CHN2	-0.97858006	5.54E-09		84031
DOK4	-0.7422019	5.64E-09		361364
SAMD4A	1.6091677	5.70E-09	Samd4	305826
ZFPM1	1.1657131	6.00E-09		691504
MAN2A1	1.3874837	6.05E-09	Mana2|Manno	25478
NETO1	0.9790565	6.11E-09	RGD1566269	307206
SLC4A7	1.6119723	6.16E-09	NBC3|NBCn1	117955
ORAI2	-1.1369072	6.24E-09	RGD1310213	304592
LRRN3	-1.0593017	6.28E-09	Nlrr3	81514
FAM89A	1.0497959	6.56E-09	RGD1309879	361441
LOC688613	-1.1505996	7.25E-09		688613
LRRTM2	-0.8271959	7.34E-09		685472
GUCY1A3	-1.0689547	7.49E-09	Gucy1a1|SGC	497757
NCBP1	0.5205126	7.53E-09		298075
RASGRP2	-0.6573872	7.58E-09		361714
RXRG	-1.9081825	7.88E-09		83574
FAR1	1.0130634	7.96E-09	Mlstd2	293173
RGD1563072	-1.1282976	8.27E-09		313595
TMEM163	1.1404581	8.67E-09	RGD1306212|Sv31	360839
BMPER	1.1796024	9.18E-09	RGD1563373	300455
PER2	0.7842451	9.52E-09	rPER2	63840
KLC4	-0.75756204	1.23E-08	1200014p03rik|Knsl8	316226
ANXA11	0.9251612	1.25E-08		290527
SF1	0.58964086	1.27E-08	Zfp162	117855
NR1D2	-0.97861964	1.31E-08	HZF-2	259241
KCNN3	1.3866298	1.32E-08	KCa2.3|SK3	54263
ARRDC1	-0.8560828	1.37E-08		366001
DDX21	0.96944666	1.42E-08	Ddx21a|Ddx21b	317399
TM4SF1	1.8135058	1.55E-08		295061
STK26	2.1175604	1.66E-08	Mask|Mst4|RGD1563568	317589
CPNE8	-0.8491475	1.71E-08		362988
CRHR1	1.1218804	1.73E-08	CRFR1	58959
KLF7	-1.2560346	1.84E-08		363243
GADD45G	1.1591874	1.88E-08		291005
DLK1	1.3777293	1.91E-08	Pref-1|Zog	114587
TMEM200C	-0.834058	1.91E-08	RGD1561734	501201
SHROOM2	-1.0640309	1.97E-08	Ab2-404|Apxl	317435
PAM	1.0347998	1.97E-08		25508
CCDC129	1.7321779	1.97E-08	RGD1561131	500139
PRRT4	-1.488174	2.00E-08	RGD1559885	500059
MAP2K4	0.80338305	2.04E-08	MKK4|Sek1	287398

POPDC2	-1.1629769	2.05E-08		360718
CAPN5	-0.91000926	2.26E-08	Htra3	171495
COL4A1	1.1251773	2.29E-08		290905
HMGCLL1	-1.0695127	2.33E-08	RGD1565090	367112
CTSF	-0.81760997	2.34E-08		361704
KLHL24	-1.0576439	2.43E-08	Dre1	303803
NFIB	-0.60702807	2.45E-08		29227
KLHL5	-0.5935278	2.49E-08		305351
RGS17	0.872459	2.49E-08		308118
RGD1564036	-0.82035017	2.69E-08		497895
RGD1561931	-0.6385058	2.73E-08	KIAA2022	302396
KLF5	0.81559616	2.88E-08	Bteb2|IKLF|bteb2	84410
PDSS2	-0.96159464	2.90E-08		365592
M6PR	0.597658	2.90E-08		312689
SLC16A14	1.4101831	2.90E-08		316578
DENND2C	1.1995537	2.96E-08	RGD1308197	295333
GOLPH3	0.58364147	2.96E-08	Gmx33	78961
MYH1	2.0129728	3.14E-08	MYHC	287408
IGSF3	-0.91093475	3.42E-08		295325
ALDH1B1	-0.90188223	3.43E-08		298079
RAMP3	-1.5966079	3.50E-08		56820
BSDC1	-0.55472773	3.50E-08	RGD1311622	297890
DUSP2	1.0111473	3.79E-08		311406
EHBP1	-0.5281663	4.14E-08	RGD1310604	305556
LZTS1	-0.97071993	4.18E-08	Psdzip70	266711
ZFP775	-0.81265396	4.21E-08	RGD1304910|Znf775	312309
LRRTM3	0.59607244	4.21E-08		294380
RET	1.0616585	4.21E-08		24716
BVES	1.2946421	4.30E-08	Popdc1	365603
SOCS3	1.5331312	4.83E-08	Cish3|Socs-3|Ssi-3	89829
WNT6	1.939307	4.89E-08		316526
SPHKAP	-1.3342793	5.50E-08	RGD1311951|Skip	316561
NAMPT	0.7191915	5.65E-08	Pbef|Pbef1	297508
NIPSNAP3B	-0.71185213	5.84E-08	Nipsnap3a	313211
ELAVL4	-1.0310686	5.85E-08	HuD	432358
USO1	0.5623836	6.19E-08	TAP|Vdp	56042
CITED1	1.1248223	6.22E-08	Msg1	64466
LOC100911664	0.53927714	6.60E-08		100911664
CMPK2	-0.8952171	6.66E-08	Tyki	314004
PRKG2	1.1620419	7.14E-08	GDPKII|cGKII	25523
CDCA7L	0.7639772	7.14E-08		619566
MAF1	-0.6388791	7.44E-08		315093
GFPT2	-1.1508073	8.02E-08		360518

DMKN	2.0456488	8.23E-08	RGD1561521	361548
CAPN2	0.5824006	8.33E-08		29154
FBXO16	-1.0601866	8.40E-08		305970
TMEM185B	0.64008254	8.72E-08	RGD1309602	304731
RHOV	-1.5801151	9.32E-08	Arhv|Chp	171581
TRAFD1	-0.48449132	9.58E-08	Fln29	114635
IER2	1.325633	9.85E-08		494344
SEC16B	1.8399274	1.10E-07	Lztr2|Rgpr	89868
INHA	-0.918004	1.12E-07		24504
LOXL2	1.6070518	1.12E-07		290350
AK4	1.0069963	1.17E-07	AK 4|Ak3l1|Ak3l2	29223
PFKFB3	1.393767	1.17E-07		117276
R3HDM1	1.0576135	1.18E-07	R3hdm	304763
SPSB1	1.363512	1.19E-07	RGD1309319	313722
ARSJ	1.7609345	1.21E-07	RGD1307640	311013
STT3B	0.8318333	1.27E-07	RGD1311563	363160
LRRN1	-0.65649915	1.29E-07		500280
NYAP1	-0.97091067	1.33E-07	RGD1310964	304376
SDCCAG8	-0.66369975	1.33E-07		305002
EIF3B	0.49294233	1.33E-07	Eif3s9	288516
ZC3H18	0.51839393	1.35E-07	Nhn1	292067
SEMA3D	1.1440028	1.39E-07		246262
MAN1C1	1.0923942	1.43E-07		362625
UTP4	0.62835366	1.46E-07	Cirh1a|Cirhin|Naic|Teg-292|	291987
IL34	-1.3838671	1.49E-07		498951
PCDH19	-0.9742993	1.51E-07	RGD1565392	317183
GPR50	2.0853536	1.51E-07	H9	117097
GMEB1	0.9823674	1.53E-07	RGD1563208	500558
FHL2	0.9380043	1.53E-07		63839
CUBN	2.0080068	1.71E-07	GP280|IFCR|MGA1	80848
KIF27	-1.0214224	1.71E-07	Krp5	246209
SPATA5	0.7741053	1.71E-07		361935
MTHFD2	0.72602046	1.71E-07		680308
PLEKHG5	1.1178672	1.75E-07	Tech	310999
FAM214A	-0.65628093	1.77E-07	RGD1310552	300836
GCK	-2.0055048	1.81E-07	GLUKA|RNGK2	24385
KDSR	-0.7911181	1.81E-07	Fvt1	360833
WASH1	-0.5980873	1.84E-07	ORF19|Wash|Wash2	367328
EXTL2	-0.638926	1.94E-07		310803
NUDT6	1.2347888	1.98E-07	Gfg	207120
THBD	1.3691242	2.02E-07		83580
FRS3	-1.0738213	2.04E-07		316213
PPAN	0.7546475	2.14E-07		298699

PSEN2	-0.95383596	2.16E-07		81751
TAF12	0.7070982	2.20E-07		682902
PIEZO1	1.3669088	2.22E-07	Fam38a|Mib	361430
POLR1B	0.85832816	2.26E-07	RPA2|Rpo1-2	83582
JAK1	0.6872115	2.32E-07		84598
ST8SIA5	1.5325679	2.32E-07	SIAT8E	364901
HEATR1	0.97933376	2.34E-07	Heatr1	103689953
NIPSNAP1	-0.55160445	2.35E-07		360971
DIAPH1	0.7853345	2.44E-07	Diap1	307483
BID	-0.71934384	2.61E-07		64625
SHROOM4	1.8252568	2.68E-07	RGD1563434	317391
CAMKK1	0.8717963	2.94E-07		60341
GPR4	1.6370957	2.95E-07		308408
COL5A1	1.3160245	3.08E-07		85490
CISH	-1.1958967	3.11E-07	Cis	83681
WISP1	1.8965559	3.13E-07		65154
SPIRE1	0.5689147	3.20E-07		307348
DCLK1	0.74735874	3.34E-07	Ania4|Cpg16|Dcamkl1	83825
KCNMB4	-0.9178091	3.37E-07		66016
MCM5	0.86161107	3.38E-07		291885
FAM189B	-0.7885296	3.38E-07	RGD1306107	310640
PDE6D	-0.7544312	3.39E-07		363272
NFE2L3	-1.3880478	3.41E-07		312331
RBM11	-1.686274	3.45E-07		288321
GPR3	1.6430944	3.48E-07	Gpcr3	266769
CTPS2	-0.5169076	3.54E-07		619580
SEMA4A	-1.0655426	3.60E-07		310630
TMEM182	1.8668323	3.73E-07	RGD1563696	501129
SH3BP5	-0.78393334	3.89E-07	SH3BP-5|Sab|Vesp18	117186
ANO1	1.9408473	3.91E-07	Tmem16a	309135
MAGEE2	-1.1971086	3.91E-07	RGD1564872	302392
PHACTR1	-0.4929978	3.94E-07		306844
CRY1	0.66173536	4.20E-07		299691
BBX	1.1857326	4.25E-07		303970
EXOC5	0.6701291	4.31E-07	Sec10l1	60627
LIPG	0.99999833	4.38E-07	lipase	291437
TUBA8	1.4122529	4.45E-07		500377
RASIP1	-0.8784184	4.49E-07		292912
PHACTR3	-0.74370235	4.52E-07	Scapin1|scapinin	362284
MORN4	-0.7344535	4.60E-07	RGD1307336	293950
GINS2	0.9516325	4.76E-07	RGD1311055	292058
TEC	1.0602809	4.79E-07		84492
LIX1	-0.71060413	4.83E-07		292381

CRELD1	-0.76312834	4.87E-07		312638
PCGF2	-0.76668996	5.66E-07	Rnf110	287662
ARIH1	0.778141	5.85E-07		300756
ABCG1	-0.99028426	5.87E-07	Abc8	85264
RXFP3	1.8421255	6.14E-07	GPCR135|RGD1311550|Rln3r	294807
KCNJ14	0.88561434	6.25E-07	Kir2.4	276720
PAK1IP1	0.8685161	6.37E-07		361232
FGF3	1.916067	6.37E-07	Int2	170633
RPP38	0.7958621	6.46E-07		291317
MYADML2	-1.8337954	6.52E-07	RGD1311499	303744
USP46	-0.5572835	6.76E-07	RGD1564808	289584
NOG	1.203545	6.77E-07		25495
FAM107B	0.8400705	6.98E-07		498796
NETO2	0.7171609	7.04E-07		307757
CHST12	-0.99370766	7.57E-07		304322
RAB33A	-1.1109589	7.82E-07	RGD1563280	317580
RNF182	-1.174699	8.11E-07	RGD1560399	498726
SMTNL2	-1.0423588	8.31E-07		679629
SFMBT1	0.7640687	8.31E-07	Sfmbt	58967
DOT1L	1.225937	8.76E-07		362831
DHX40	-0.69222575	8.81E-07		287595
NOL10	0.62870246	9.12E-07		313981
PHLDA1	1.0072471	9.22E-07	Tdag	29380
TYRO3	0.7946464	9.49E-07	Brt	25232
EBF3	1.7820029	9.59E-07		361668
AGBL5	-0.7093159	9.90E-07		362710
NEUROD1	-1.4374832	9.93E-07		29458
MUM1	-0.8432682	1.11E-06		362838
HEPHL1	2.001065	1.14E-06	RGD1564835	500946
RHBDD3	-0.65523726	1.19E-06	RGD1311827	289753
PM20D2	-0.5811111	1.20E-06	Acy1l2	313130
MFSD6	-0.62734413	1.21E-06	RGD1562317	301388
TRIM23	-0.6204789	1.21E-06	Ard1|Arfd1	81002
DLEU7	-1.3473	1.24E-06	RGD1305071	290308
PAK6	0.85644025	1.27E-06		296078
RHBDF2	-1.5396582	1.28E-06	Rhbdl6	303690
DMTN	-0.46067888	1.31E-06	Epb4.9|Epb49	361069
TMEM169	-0.63025296	1.32E-06		690294
SV2B	-1.1675445	1.33E-06		117556
FURIN	0.85177886	1.40E-06	Pace|Pcsk3	54281
CACNB1	0.829659	1.48E-06		50688
ACKR3	-0.89520156	1.50E-06	Cmkor1|Cxcr7|Rdc1	84348
GPR88	0.56655765	1.55E-06		64443

ZDHHC14	1.0382525	1.59E-06	Dhhc14	499014
KMT2E	-0.5801543	1.67E-06	Mll5	311968
FDFT1	-0.5545826	1.74E-06		29580
S100A10	0.73875827	1.77E-06	p11	81778
APOLD1	1.394856	1.77E-06	Verge	444983
KCNH4	-1.2192159	1.78E-06	Bec2	114032
LRSAM1	-0.7647853	1.81E-06	RGD1564403	311866
KLHL23	-0.50264704	1.82E-06	RGD1307166	311114
TAC1	1.7067176	1.82E-06	PPTA3|Ppt5fl|RATPPTA3|TA(	24806
SLC30A3	1.1141921	1.84E-06	ZnT3	366568
RIN1	1.6419429	1.84E-06		207119
ACTR3B	-0.59213006	1.90E-06	RGD1565759	362298
CTRB1	1.2300013	1.91E-06	Ctrb	24291
OPRL1	-0.82617456	1.97E-06	KOR-3|KOR3|LC132|MOR-C|	29256
FYTTD1	0.55285794	1.99E-06	Ac1176|RGD1306899	360726
JAK2	0.8380113	1.99E-06		24514
ANKS1B	0.6663737	2.02E-06	AIDA-1|EB-1|RGD1565556	314721
HP1BP3	-0.42026517	2.03E-06		313647
HSF1	0.59237087	2.05E-06		79245
WDR45B	0.736058	2.07E-06	RGD1305141|WIPI-3|Wdr45l	360682
RAB30	-0.98914146	2.08E-06	Rsb30	308821
FLRT1	1.3457637	2.08E-06	RGD1565152	499308
MRTO4	0.5574501	2.12E-06	RGD1311709	298586
GRM1	-1.2940531	2.16E-06	Gprc1a	24414
PTGER4	1.7010512	2.17E-06	EP4|Ptger|Ptgerep4	84023
SH2B2	-0.9922354	2.18E-06	Aps	114203
NUAK1	-1.0095615	2.19E-06	RGD1309956	299694
NOP56	0.6859545	2.21E-06	Nol5a	362214
C1S	0.96534324	2.28E-06	r-gsp	192262
ANKRD34A	-0.8755667	2.31E-06	Ankrd34|RGD1308412	295283
UBE2G2	0.7997212	2.34E-06		294331
ACVR1C	1.5148302	2.40E-06	Alk-7|Alk7|habrec1	245921
P2RY14	1.9522917	2.41E-06	Gpr105	171108
EZH1	-0.90584284	2.46E-06		303547
CD59	-0.5351856	2.66E-06	Cd59a|Cd59b|MAC-IP|MACII	25407
RGD1305537	0.8518127	2.66E-06		363528
MXD4	-0.6188515	2.75E-06		360961
RWDD2A	-1.2374972	2.75E-06	Rwdd2	363110
LEF1	1.3694166	2.80E-06		161452
MAP7D1	0.87675613	3.05E-06	Mtap7d1	681287
GPR22	0.5187866	3.07E-06		298944
MLLT3	0.5354938	3.07E-06	Af-9|Af9	114510
GRM6	-1.2690232	3.12E-06	mGluR6	24419

MED15	0.88584226	3.17E-06	Pcqap	360743
TRMT61A	0.90787005	3.18E-06	Gcd14|RGD1359191|Trm61	314462
PNRC1	-1.1859689	3.19E-06	Prol2	286988
TRIM24	0.5507146	3.20E-06		500084
RYK	0.48857123	3.21E-06		140585
CCDC184	-0.8354163	3.21E-06		500925
PNPLA3	0.5412728	3.25E-06	Adpn	362972
DDX3X	0.61162984	3.26E-06		317335
CBX8	-0.77355605	3.33E-06		303731
AMOTL2	-0.72246194	3.36E-06	Lccp	65157
MUSTN1	1.5033047	3.40E-06	Mustang	290553
SMR3B	1.9007351	3.43E-06	Arp|P2-VA1|RATSMR1A|Smr	24867
PPP4R1	0.5003029	3.52E-06	Pp4r1	140943
WIPI2	0.59669757	3.53E-06		288498
CHRNA5	-1.0467976	3.57E-06		25102
SEMA4G	-0.8667875	3.59E-06		361764
ANKRD6	-0.76407593	3.61E-06		500430
STK40	0.6637745	3.61E-06	Lyk4|RGD1307310	360230
PSD2	-0.88123	3.78E-06		307500
PTK2	0.50821215	3.91E-06	FAK|FRNK|p125FAK	25614
ADAMTS1	1.6356838	3.93E-06		79252
TMEM2	0.9436111	3.98E-06		309400
CAMK1G	0.65445167	4.01E-06		171358
DUSP23	-1.3225392	4.08E-06		360881
SLC6A15	-0.55569607	4.13E-06	Ntt73	282712
EVA1A	1.090327	4.15E-06	Fam176a|RGD1559797|Tmei	500221
FLOT2	-0.824491	4.20E-06		83764
UCK2	0.7105085	4.28E-06	Umpk	304944
RCAN2	0.67361695	4.29E-06	Dcip2|Dscr1l1|Zaki-4	140666
SLC17A5	-0.5625441	4.46E-06		363103
GLRB	-0.499963	4.53E-06		25456
POLG	1.0007834	4.56E-06		85472
KY	-1.4094783	4.59E-06		315962
LRRC28	-0.76801777	4.67E-06		361588
KLHL7	-0.6506814	4.74E-06		362303
SNAP47	-0.87896234	4.74E-06	RGD735194|SNAP-47	303183
42984	-0.6011752	4.76E-06		691335
CHODL	-1.4929341	4.78E-06		288289
RGD1560108	-0.43833774	4.79E-06		499309
EDRF1	0.54816943	4.90E-06	RGD1306820	309069
CNTFR	0.72213113	4.95E-06		313173
LAMB3	1.7776339	5.01E-06		305078
HS6ST1	0.5523936	5.03E-06		316325

PKD2	0.824576	5.14E-06	RGD1559992|Trpp2	498328
CTBP2	0.5165653	5.20E-06		81717
POP1	0.5550531	5.20E-06		315045
ARHGAP17	0.8164248	5.39E-06	Rich1	63994
KLF2	1.3385966	5.52E-06	Lklf	306330
SEMA4F	-0.8862101	5.93E-06		29745
CDT1	0.92074645	6.04E-06	Ris2	292071
CD200	-0.64339024	6.06E-06	Cspmo2|MRCOX2|Mox2	24560
SIDT1	-0.6057637	6.46E-06		288109
SIGMAR1	0.45852426	6.50E-06	Oprs1	29336
GPR165	-1.2783878	6.53E-06	RGD1564566	296866
ADGRB3	-0.7330781	6.67E-06	Bai3	301309
THAP3	-0.98641676	6.70E-06	RGD1308109	362667
MBNL2	-0.6090734	7.04E-06		680445
SERPINE1	1.582575	7.13E-06	PAI1A|Pai1|Pai1aa|Planh|RA	24617
GBX1	1.7539651	7.19E-06		246149
ZFP746	0.50782764	7.19E-06	RGD1306209|Znf746	312303
GABRA1	-0.7911423	7.22E-06		29705
DIP2C	1.0089853	7.51E-06	RGD1560155	307067
PPP2R2B	-0.44699788	7.90E-06	Pppr2b2	60660
ARHGAP20	-1.062759	7.97E-06	RARhoGAP	367085
GPR6	-1.4827788	8.05E-06		83683
GMEB2	1.0013711	8.15E-06		83635
PINX1	0.8684557	8.17E-06	RGD1566025	305963
ROCK2	0.7401482	8.20E-06	ROCK-II|ROK	25537
HMGA2	1.7194638	8.42E-06	Hmgic	84017
DDX20	0.65328026	8.54E-06	Dp103	84473
LOC682102	-0.9888662	8.75E-06		682102
RND3	0.6508618	9.11E-06	Arhe|RHOE	295588
AIPL1	1.7215822	9.75E-06		59110
PRSS35	1.2670162	9.79E-06		315866
RXRB	-0.709745	1.02E-05	RXR-beta	361801
IFT88	-0.60537183	1.02E-05	Ttc10	305918
TOR1B	0.5286337	1.02E-05		311854
RNF39	0.929221	1.02E-05	Hzfw1|Lirf	171387
LRRC17	0.96172076	1.03E-05		502715
TMEM143	-0.8737467	1.03E-05	RGD1305013	308593
SH3GL1	0.6641487	1.05E-05	SH3P8	81922
GNAI1	0.4840802	1.07E-05	BPGTPB	25686
AHI1	0.7709468	1.11E-05	Ahi-1	308923
CRLF3	0.7767652	1.12E-05	Cytor4	54395
REEP1	-0.51963574	1.12E-05	RGD1305230	362384
LRRC56	-0.7891569	1.13E-05	RGD1311654	365389

CALCOCO1	-0.6872678	1.13E-05		246047
ARHGEF9	0.41439357	1.13E-05		66013
GFOD1	1.3111984	1.13E-05		306842
DDIT3	1.1716156	1.13E-05	CHOP|CHOP-10|Chop10|Gac	29467
AEN	0.58869857	1.14E-05	Isg20l1|RGD1305051	361594
SUOX	-0.5694383	1.15E-05		81805
ETF1	0.7109775	1.15E-05		307503
SLAIN2	0.45768374	1.17E-05	RGD1311593	305310
GPC2	-0.5883749	1.19E-05		171517
MNT	-0.8582651	1.21E-05		287521
KLHL36	-0.8790037	1.22E-05		498957
GTPBP4	1.0413588	1.22E-05	Crfg|RGD1563564	114300
PLK3	0.5974189	1.25E-05	Cnk|Fnk	58936
ACSL3	0.60574263	1.27E-05	Acs3|Facl3	114024
CHST8	0.5964559	1.27E-05		308511
DISC1	1.3237586	1.28E-05		307940
FBN2	1.3433425	1.30E-05		689008
SKP2	0.5353479	1.30E-05	RGD1562456	294790
USP16	0.87004346	1.32E-05		288306
FAM65B	0.9654181	1.32E-05	Ab2-162|RGD1306939	306934
FZD1	-0.8275878	1.33E-05		58868
UST	1.0437173	1.34E-05		361450
AGFG1	0.48620692	1.34E-05		363266
ADRA1A	1.5817711	1.34E-05	Adra1c	29412
APC2	-0.98123884	1.36E-05		299611
ZFP786	-0.7909573	1.41E-05		100158223
RGS12	-0.6019134	1.41E-05		54292
SRPK1	0.5421408	1.42E-05		361811
PNOC	1.4730601	1.42E-05	N23K|Npnc1	25516
JADE3	1.0835265	1.43E-05	RGD1563945	299305
GPR85	-0.59075433	1.43E-05	Srep2	64020
SCN3B	-0.73834014	1.48E-05	Scnb3	245956
MYO9B	0.7637376	1.48E-05		25486
SMPD3	-0.6857319	1.49E-05	cca1	94338
GPR139	-1.578461	1.50E-05		293545
DOCK8	1.2737018	1.55E-05		499337
KBTBD8	0.83205956	1.59E-05	RGD1563166	500262
FAM98A	0.43262425	1.65E-05	RGD1305486	313873
QPCT	-0.9462288	1.66E-05	Qpctl1|RGD1562284	313837
RAP1B	0.7506237	1.66E-05		171337
RGD1359127	0.82780486	1.69E-05		299612
LOC314140	0.6524896	1.70E-05		314140
LGI1	-1.0688856	1.70E-05		252892

ADORA2A	-1.735874	1.75E-05	A2ar|ADENO|Adora2l1	25369
DES	1.4596198	1.75E-05		64362
FAM185A	0.63159645	1.78E-05	RGD1562135	499979
IQSEC3	0.91643864	1.78E-05	sag	404781
GRPEL2	0.7165067	1.81E-05	Afap1l1	688777
CDR2	0.5496149	1.81E-05	RGD1310578	308958
TDRD7	0.48013663	1.84E-05	Pctaire2bp	85425
QTRT2	0.6236451	1.88E-05	Qtrtd1	288364
SPTSSB	-1.2580304	1.89E-05		100362555
WBSCR22	0.64965624	1.89E-05		368084
STRADB	-0.4698745	1.90E-05	Als2cr2|RGD1559449	501146
TAF1C	0.68430364	1.90E-05		361420
FAM110D	-1.0217975	1.93E-05	Grrp1|RGD1564994	500563
CEP70	-0.81666386	1.93E-05		367153
ARHGEF28	-0.7162797	1.96E-05	Rgnef	361882
CLN8	-0.578793	1.97E-05		306619
CDC14A	1.0829693	1.97E-05		310806
TOX3	-0.69983387	2.04E-05	Tnrc9	291908
UTP20	0.86299187	2.14E-05	RGD1560606	314713
BTBD10	0.4444746	2.15E-05	RGD1306301	308890
MB21D2	-0.5345009	2.21E-05	RGD1559643	498100
RGD1306227	-0.7342393	2.21E-05		310377
FYN	0.42917925	2.24E-05		25150
SPIRE2	-0.7114268	2.24E-05		307925
USP7	0.71191597	2.34E-05	Hausp	360471
TRIQK	-0.8763315	2.35E-05		500413
GPR52	-1.0711756	2.39E-05		684623
POLL	-0.72658974	2.41E-05		361767
EAF1	0.6044422	2.41E-05		306261
MAT2B	-0.71800137	2.59E-05		683630
APLP2	0.4543415	2.60E-05	APLP-2|Apph|WALPLP2	64312
HOMER2	0.74575526	2.72E-05	Vesl-2	29547
CCNF	0.7937148	2.72E-05		117524
CCDC28A	-1.1651317	2.75E-05	RGD1310326	361454
CARF	-0.8988426	2.77E-05	Als2cr8	301446
CSNK2A2	0.50321984	2.78E-05		307641
ING4	-0.56335753	2.79E-05		297597
RAB20	1.3043	2.80E-05		689377
CD63	0.48863277	2.87E-05		29186
ZFP90	-0.8119344	2.88E-05		498945
KL	1.7558788	2.90E-05		83504
NOC3L	0.7142255	2.90E-05	RGD1560656	361753
SYTL5	1.532306	2.90E-05		302538

USP36	0.6773198	2.91E-05		303700
EPHA3	-1.1606913	2.95E-05		29210
LRFN5	-0.6818887	2.95E-05		314164
TMEM25	-0.8241418	2.97E-05		689172
ZFYVE9	0.61857194	2.99E-05		313477
GCLC	0.66398966	3.03E-05	Glclc	25283
PAK7	-0.50225145	3.05E-05	PAK-5|PAK-7	311450
AKIRIN1	0.4189056	3.07E-05		595134
DGKA	0.7506357	3.07E-05	Dagk1	140866
ST3GAL3	-0.68850714	3.15E-05	Siat6	64445
IGSF8	-0.7526169	3.16E-05		304979
NR4A1	0.8427543	3.23E-05	HMR|Ngfi-b|Nur77	79240
PDE8A	1.5737009	3.23E-05	RNPDE8A	308776
PLAA	0.5160917	3.25E-05	PLA2P|Plap	116645
GOLGB1	-0.776303	3.28E-05		192243
FNDC3B	1.1798513	3.28E-05	RGD1311673	294925
TRMT10C	0.54266685	3.33E-05	Rg9mtd1	304012
LMO7	-0.53625363	3.35E-05		361084
ZFP180	0.539561	3.35E-05	rKr1	246279
NSUN2	0.41097412	3.38E-05	RGD1311954	361191
CPNE4	-0.5717217	3.45E-05		367160
PLEKHA3	0.5255029	3.53E-05		295674
SNTG2	-1.1350543	3.58E-05		298936
PLPP6	-0.62889516	3.58E-05	Ppapdc2	619549
SLC30A4	0.54896474	3.58E-05		64469
PUS3	0.74455327	3.64E-05	RGD1310757	315554
LOC100125362	0.5085785	3.65E-05		100125362
AUH	-0.6037643	3.67E-05		361215
DIO3	1.3018848	3.67E-05	5DIII|DIOIII	29475
LPL	-0.6849811	3.69E-05		24539
ABI1	0.47359344	3.74E-05	E3b1	79249
LRRC20	-0.63568723	3.79E-05	RGD1566140	499430
ZFP217	1.2785103	3.79E-05	Znf217	311764
SPTBN4	0.690293	3.86E-05	Spnb4	308458
PXDC1	0.7435586	3.88E-05	RGD1311307	361238
ZBTB2	0.50025356	3.90E-05		308126
LZIC	-0.4655248	3.93E-05		366507
ARRDC4	-1.01276	3.93E-05	Ab1-209|LRRG00117	293019
FHDC1	0.9313067	3.99E-05	RGD1311955	295161
MFAP3L	1.1268398	4.04E-05		306424
DLG1	0.9289756	4.09E-05	Dlgh1|SAP97	25252
GPRC5C	-1.4309026	4.09E-05		287805
STOML1	-0.912747	4.12E-05	RGD1559463	300748

CDC6	0.76084006	4.13E-05		360621
MAML3	1.4926767	4.14E-05	Glrp1	310405
MICU3	0.52570915	4.16E-05	Efha2	364601
CSNK1A1	0.44439343	4.18E-05		113927
PIANP	-0.7880445	4.18E-05	PANP|RGD1304952|leda-1	312711
TARS	0.61816424	4.22E-05		294810
PAPPA	1.6625159	4.26E-05		313262
SNX9	0.6418308	4.33E-05		683687
GMPS	0.5733719	4.34E-05		295088
HSPA1B	1.301831	4.35E-05	HSP70.2|Hsp70-1|Hsp70-2|H	294254
FFAR4	1.700831	4.36E-05	Gpr120|O3far1	294075
PDE4C	1.7066025	4.45E-05	Dpde1	290646
RYBP	0.52183354	4.59E-05		312603
GPR63	1.2530905	4.71E-05		297952
GSPT1	0.6912279	4.78E-05		24420
MAPK15	-1.170575	4.78E-05	Erk7	286997
CNTN2	-1.07381	4.82E-05	TAG-1|TAG-564|TAG1|Tax	25356
NFX1	0.3948233	4.82E-05		313166
KCNJ12	0.87608784	4.89E-05	IRK2|Kir2.1|Kir2.2	117052
NOLC1	0.45543104	4.90E-05	Nopp140	64896
AFTPH	0.57212704	4.90E-05	RGD1311920	305544
CUX2	-0.9501184	4.99E-05	Cutl2	288665
CHST7	0.7684695	5.02E-05	Gn6st-4	302302
CDH17	1.6838155	5.18E-05		117048
TBC1D17	-0.6419177	5.20E-05		292886
ITM2C	-0.7251217	5.24E-05		301575
GSPT2	0.48441496	5.29E-05	RGD1563213	501582
CDK13	0.6838744	5.43E-05	Cdc2l5	306998
SRD5A1	-0.5419333	5.63E-05	S5AR 1	24950
LOC688459	1.670172	5.78E-05		688459
KPNA4	0.6832937	5.95E-05	importin	361959
SLC35E3	0.3921884	5.99E-05	RGD1564876	362883
RABGEF1	0.44292256	6.03E-05		360797
TTC8	-0.66576236	6.14E-05		299246
PGAM1	0.51403296	6.19E-05	PGAM-B|Pgmut	24642
LPCAT2	0.8675699	6.19E-05	LPCAT-2|RGD1563994	100359680
LSS	0.47092003	6.24E-05		81681
DNAJC12	-0.8063608	6.25E-05	Jdp1	619393
MIOS	0.52154845	6.26E-05	RGD1308432	362324
NUTM1	1.1681896	6.33E-05	Nut|RGD1564529	366153
VWC2L	-1.4620417	6.33E-05	RGD1565501	501160
ADAMTS5	1.2069982	6.36E-05		304135
ZBTB5	-0.68663055	6.41E-05		298084

COBL	-0.50902236	6.44E-05		305497
EPHB6	-0.86060816	6.53E-05		312275
MFSD9	-0.6942437	6.59E-05	RGD1562212	316356
TTC9	0.6224589	6.69E-05	RGD1565985	500689
IL17RA	0.61163193	6.82E-05	Il17r	312679
MAFK	-0.61563605	7.17E-05		246760
RNF150	0.78642243	7.18E-05	RGD1304572	364983
ZFAND5	0.5366909	7.23E-05	Zfp216	293960
PDIK1L	-0.41942006	7.28E-05	RGD1307476|Stk35l2	313609
HAS1	1.446656	7.31E-05		282821
FAM118A	-0.7019109	7.37E-05	RGD1560783	300120
CX3CL1	0.60973	7.42E-05	Cx3c|Scyd1	89808
CUL9	-0.67350787	7.45E-05	Parc|RGD1562008	316228
EIF4A1	0.7466136	7.47E-05		287436
MMS22L	0.6067737	7.50E-05	RGD1304693	313108
PNCK	-0.8705656	7.59E-05	Camk1b	29660
CHRM2	1.338223	7.59E-05	Acm2	81645
ARRB2	-0.75203335	7.63E-05	BARRES	25388
UHRF1BP1L	0.48176548	7.63E-05		363009
42987	0.5782184	7.70E-05	Eseptin|Msf|Slpa	83788
BAD	-0.535574	7.98E-05		64639
ACTN4	0.5552806	8.00E-05		63836
MTMR14	-0.68917155	8.07E-05	RGD1304842	312634
TEF	-0.535716	8.07E-05		29362
SYK	0.8088468	8.11E-05	p72syk	25155
BBC3	-0.8880315	8.11E-05	Puma	317673
TMEM53	-0.9829783	8.27E-05	RGD1307066	313529
TOR4A	1.0359523	8.44E-05	RGD1308019	311795
CLIP2	-0.5369201	8.51E-05	CLIP-115|Cyln2	29264
XKR8	0.7255713	8.53E-05	RGD1305649|XRG8	313033
EPDR1	0.46413752	8.55E-05	Epdr2|MERP2	291180
DIRAS1	-0.8976908	8.84E-05		366826
IFNGR2	-0.4437649	8.88E-05		360697
PRKAR1A	0.49870515	8.98E-05	RIIA	25725
KLHL14	-1.0380788	9.21E-05	RGD1566178	364823
DDX18	0.685768	9.26E-05	MrDb	308490
OTUD3	0.9555002	9.28E-05	RGD1560468	500572
ARID3C	1.5952957	9.28E-05	RGD1560943	502946
MAST1	-0.60119736	9.42E-05	Sast	353118
CIRBP	-0.72022355	9.57E-05		81825
ARF6	0.44755846	9.57E-05		79121
ZHX1	-0.44386098	9.62E-05		171159
DDAH2	-0.56704617	9.77E-05		294239

ARG2	0.5525799	1.01E-04		29215
EPB41L4B	1.0035497	1.02E-04	RGD1562988	500464
UNC119	-0.6921164	1.03E-04	RRG4|Uncl19	29402
AMIGO2	-0.78318065	1.03E-04	Ali1	300186
CYS1	-0.99969214	1.03E-04		690489
POU6F1	0.9946661	1.03E-04	Brn5	116545
VIPAS39	-0.49750003	1.04E-04	Spe39|Vipar|hSPE-39	681989
GDNF	1.6205387	1.07E-04	gndf	25453
MPEG1	-1.1206188	1.09E-04	Mpg-1	64552
PALMD	-0.99204695	1.10E-04		310811
CXCR4	-0.614934	1.11E-04		60628
COQ10B	0.5414451	1.11E-04	RGD1359509	301416
LDLRAD4	0.8536135	1.11E-04		679578
LUZP1	0.72387207	1.11E-04	Luzp	79428
PPRC1	0.7030932	1.13E-04		294007
MSS51	0.8746994	1.14E-04	Zmynd17	289904
TPPP3	-1.1469681	1.15E-04	CGI-38|RGD1305061	291966
VPS9D1	-0.5139628	1.16E-04	RGD1565149	307923
CTU2	0.70760155	1.16E-04	Ncs2	292069
LYPD1	-0.7614784	1.17E-04	Lypdc1	360838
SPEM1	0.72259897	1.18E-04		691981
USP28	0.65976477	1.21E-04		315639
PRR15	1.2841624	1.21E-04	RGD1311589	312358
CEP131	-0.7585036	1.23E-04	Azi1	360672
WFS1	0.7863216	1.23E-04		83725
PARP16	-0.77560216	1.23E-04	ARTD15|LRRGT00109|RGD13	315760
COX19	-0.611949	1.24E-04	RGD1305631	304330
SLC35F2	-0.8826118	1.25E-04		300713
LRRC10B	1.3151493	1.25E-04	RGD1564983	309208
STEAP1	1.3752855	1.25E-04	Steap	297738
LMO2	1.2145776	1.26E-04		362176
BMP6	0.74068916	1.27E-04	VGR	25644
GRIN1	0.56461716	1.36E-04	GluN1|NMDAR1|NR1	24408
PTCHD1	1.2826488	1.36E-04	RGD1564527	317517
ARPC5	0.51324344	1.36E-04		360854
FAM136A	-0.5223806	1.39E-04	RGD1304825	297415
CYP2S1	-1.3139243	1.41E-04		308445
PNPO	-0.6106862	1.41E-04		64533
MVK	-0.59577304	1.42E-04	Lrbp	81727
IMPDH2	0.5349753	1.44E-04	IMPD 2|IMPDH 2	301005
SOGA3	-0.4308755	1.45E-04	Kiaa0408|RGD1307525|Rgd1	292199
CHRNA3	-1.1553017	1.46E-04		25101
TRPV2	-0.7396124	1.49E-04	Vrl1	29465

B4GALT7	-0.6417235	1.52E-04		364675
ZFP467	-0.6218755	1.52E-04	Znf467	500110
GPSM2	0.4762065	1.52E-04	RGD1560967	362021
TBKBP1	0.5302589	1.55E-04	Prosapip2	266764
ICK	-0.6124105	1.57E-04		84411
NAB1	0.438405	1.61E-04		64824
SLC18A2	0.5498699	1.62E-04	MNAT|VMAT-2|VMAT2	25549
PAPOLA	0.44860807	1.63E-04		314417
RALYL	-0.802912	1.65E-04	RGD1305844	294883
ZFAT	0.6622704	1.66E-04	Zfat1|Zfp406	362925
TGFBR2	1.289802	1.66E-04	TGF-beta 2|Tgfbr2T	81810
POLR1C	0.42630336	1.67E-04	Ac2-127|Rpo1-1	301246
PTER	1.0322663	1.69E-04	Rpr-1|Rpr1	63852
CDYL2	0.95827675	1.69E-04		292044
AKAP7	-0.7377203	1.69E-04	AKAP-18|AKAP18d|Akap15|A	361458
EFNB3	-0.5920111	1.69E-04	ELK-L3	360546
GIPC3	1.6110032	1.69E-04	RGD1563255	500789
NOCT	0.5487951	1.74E-04	Ccr4|Ccrn4l|Ccrn4lb	310395
BCAS3	-0.5178273	1.74E-04	RGD1560788	363662
DAZAP1	0.45886728	1.75E-04		362836
MXI1	-0.47463524	1.76E-04	MXI-WR	25701
HDAC5	0.48885363	1.77E-04		84580
MC5R	1.6079197	1.77E-04		25726
POMK	-0.58920217	1.78E-04	RGD1310810|Sgk196	306549
PODXL	-0.6923312	1.78E-04	PC|PCLP-1|podocalyxin	192181
IRF6	1.2191257	1.82E-04		364081
PECAM1	-1.3774275	1.82E-04	CD31|Pecam	29583
TRPV6	-0.93850434	1.82E-04	CaT1|Ecac2|Otrpc3	114246
RGD1308117	-0.9308683	1.82E-04		361066
ADAMTS2	-1.2053839	1.84E-04	RGD1565950	287899
TNFRSF12A	1.0359695	1.85E-04	Fn14	302965
ACOT3	-0.62287575	1.85E-04	RGD1564089	314304
MMP17	0.8486851	1.88E-04		288626
TMEM68	-0.5819448	1.88E-04	RGD1309006	312946
METTL13	0.46811473	1.89E-04	RGD1311526	289159
GALNT16	-0.53217185	1.91E-04	Galntl1	362760
DAB1	0.71107423	1.92E-04		266729
IL1RAP	0.9475349	2.00E-04	IL-1RAcp|Il1racpb	25466
ETV4	0.7502681	2.00E-04	Pea3	360635
PITX1	1.5863794	2.00E-04	Bft	113983
CLASP2	-0.45628664	2.00E-04		114514
TADA2B	0.43645033	2.01E-04	ADA2B|RGD1561605	289717
LTBP1	0.91750896	2.01E-04		59107

DUSP26	-0.6058669	2.02E-04	RGD1310090	306527
ADAMTS7	-0.74705875	2.04E-04	ADAMTS7B|COMPase	315879
FAIM2	-0.72629684	2.05E-04	Lfg|NMP35	246274
CYB5R1	-0.7076814	2.05E-04	Nqo3a2	304805
MEPE	1.3387411	2.06E-04		79110
PTPN1	0.64497983	2.07E-04	Ptp	24697
TPM4	0.82039404	2.07E-04	Tpm4.2|Tpm4.2cy	24852
RGS2	0.7381537	2.08E-04		84583
TEX10	0.5948173	2.11E-04		298065
KCNB1	0.86163217	2.11E-04	DRK1PC|Kcr1-1|Kv2.1|Shab	25736
CBFB	0.6844125	2.12E-04	Pebp2	361391
TP73	1.3698492	2.13E-04	P73|Trp73	362675
STAU1	0.3779561	2.14E-04	Stau	84496
ARL14EP	0.7410797	2.14E-04	ARF7EP|RGD1311463	311279
IGF2	1.5672538	2.17E-04	IGFII|RNIGF2	24483
CDK19	-0.5549146	2.17E-04	Cdc2l6|Cdk11	309804
LRRC59	0.4165463	2.20E-04		287633
RRS1	0.7456884	2.28E-04		297784
ANKRD12	-0.6539705	2.30E-04		316775
LRRC4	0.68762976	2.31E-04		641521
RAB15	0.59578437	2.36E-04		299156
PPP2R3A	-0.8871097	2.40E-04		363122
OS9	-0.4035507	2.40E-04	Os-9	362891
SEMA3E	0.8192995	2.40E-04		296789
CMAS	-0.65439934	2.42E-04		312826
OPRD1	1.2956082	2.46E-04		24613
NLN	0.36868855	2.46E-04		117041
HSD11B1	1.0060592	2.47E-04	LRRGT00065	25116
MRRF	-0.47376493	2.50E-04		311903
42803	-0.62812364	2.53E-04	March-IX|Rnf179	679272
LRTM2	-1.2352533	2.57E-04		680883
LHX2	-0.7194863	2.57E-04	LH2A|Lh-2	296706
GADD45A	0.8438053	2.60E-04	Ddit1|Gadd45	25112
PLCL2	0.49759674	2.67E-04		301173
UTP15	0.69488347	2.68E-04	RGD1310992	310019
PRUNE	-0.5479131	2.71E-04		310664
SMOC2	-1.477256	2.71E-04		292401
YARS	0.430864	2.71E-04		313047
RRP8	0.6214512	2.71E-04	RGD1308302	308911
ARHGAP31	1.1611425	2.71E-04	Cdgap	288093
DESI1	0.42232907	2.72E-04	DeSI-1|Fam152b|Pppde2|RG	315160
RHEB	0.5235065	2.74E-04		26954
ASGR1	1.3252411	2.80E-04	ASGR|RATRHL1|RHL1	24210

PFKP	0.4141759	2.84E-04	ATP-PFK|PFK-C|PFK-P	60416
P4HA1	0.66392094	2.85E-04	PHalphaI	64475
ARMC7	-0.6029792	2.85E-04	RGD1310043	287827
SFMBT2	1.2059495	2.86E-04		307106
OSBPL6	0.9556358	2.87E-04		311129
USP20	-0.5476291	2.93E-04		311856
ADAMTS15	-0.94080544	2.93E-04		300474
CTDP1	0.4451366	2.93E-04		291414
ZW10	0.4267897	2.94E-04		363059
AP3S2	-0.52299076	3.01E-04		683402
CDYL	0.79401857	3.01E-04		361237
E2F4	0.48663586	3.02E-04		100360427
NDRG1	0.6972355	3.02E-04	Ndr1	299923
PPM1H	0.7127094	3.03E-04		314897
NCOR2	0.8484824	3.11E-04		360801
NFATC1	0.7563985	3.12E-04		100361818
RGD1311164	-0.46310118	3.15E-04		297607
SLC6A7	-0.712864	3.28E-04	Prot	117100
NFRKB	0.6441671	3.31E-04		315523
ABHD6	-0.4444501	3.32E-04		305795
KCND2	-0.5871494	3.33E-04	Kv4.2|RK5|Shal1	65180
NRSN1	0.46664348	3.35E-04	Vmp	291129
ADD3	-0.5705513	3.37E-04		25230
CYB561	-0.60420316	3.39E-04		303601
ELP2	-0.41660187	3.39E-04	Statip1	307545
OSBPL1A	-0.36456922	3.50E-04		259221
RGD1312005	-0.9231772	3.50E-04		291580
AMIGO3	0.73918504	3.52E-04		316003
TEX264	-0.50394505	3.53E-04		300988
SIRT5	-0.6803784	3.54E-04		306840
ABCD1	-0.59828013	3.59E-04	RGD1562128	363516
ATP13A3	0.7735666	3.59E-04		678704
ARFGAP3	-0.45546272	3.60E-04		503165
RALGDS	-0.51777	3.62E-04	Rgds	29622
ADGRE5	1.0853325	3.64E-04	Cd97	361383
CEBPA	0.7842719	3.67E-04	DBPCEP	24252
ATP5SL	-0.4827523	3.72E-04		361520
IFT46	-0.6445975	3.72E-04	RGD1307682	300675
YTHDF3	0.40102714	3.86E-04		361920
ESPNL	1.5138139	3.86E-04	RGD1562432	301606
NIP7	0.4737033	3.90E-04	CGI-37|Nip7p|pEachy	192180
LOC691141	1.0126132	3.96E-04		691141
BLOC1S6	-0.54068834	4.01E-04	Pldn	317630

LRRC8B	1.0917848	4.03E-04	RGD1563429	305135
ECH1	-0.6108725	4.03E-04	Pxel	64526
KDM1A	0.36938342	4.03E-04	Aof2|Kdm1|RGD1562975	500569
DNASE2B	1.5245335	4.18E-04	Dlad|UOX	59296
CYP11B2	1.5138268	4.25E-04	Cp45as|Cyp11b3|RNCP45AS	24294
SERTM1	0.830482	4.26E-04		690333
ANAPC2	-0.45406723	4.37E-04		296558
CAND1	0.57176256	4.39E-04	Tip120|Tip120A	117152
FAM20B	0.37168434	4.45E-04	RGD1311162	304885
TCP11L2	-0.7020828	4.48E-04	RGD1307494	314683
SRC	0.7262243	4.55E-04		83805
NEBL	0.81218123	4.55E-04	Lasp-2	307189
TMIE	-1.1572524	4.56E-04	RGD1562523	501061
QPCTL	-0.47004032	4.56E-04	RGD1308128	292687
SERPINB7	1.505848	4.56E-04	Megsin	117092
NOC4L	0.5610823	4.58E-04	RGD1310661	360828
HAGH	-0.6924093	4.59E-04	Glo2|RSP29	24439
PCDHA2	-0.5102854	4.59E-04	rCNRv02	393086
TSPAN2	-0.4740401	4.59E-04	Tspan-2	64521
NRIP3	0.5417548	4.59E-04		361625
DSE	0.5761879	4.59E-04	Sart2	365583
TRMT44	0.69999003	4.66E-04	Mettl19|RGD1308380	305443
PCDHA12	-0.5639758	4.67E-04	rCNRv12	116779
OXTR	1.3693583	4.69E-04	OT-R|OTR|OTR1	25342
ZBTB22	-0.48097795	4.72E-04	Bing1|Zfp297|Znf297	309630
REM2	0.7931316	4.73E-04		64626
PRRT1	-0.61716336	4.87E-04	DSPD1|Ng5|Orf31	406167
GRHL2	1.4127645	4.90E-04	RGD1561191	299979
RBBP5	0.38272616	4.91E-04		304794
CLK3	0.43377548	4.91E-04		171305
AGPAT5	0.37164775	4.95E-04	RGD1306405	306582
PRKX	0.47665304	4.96E-04		501563
BTAF1	0.86330533	5.02E-04	RGD1564130	368042
PCSK4	-1.0208651	5.09E-04		171085
MPPED1	-0.6468225	5.10E-04	RGD1308244	362971
RGD1310110	-0.6975931	5.15E-04		361032
DNAJC25	0.5981939	5.15E-04		362526
H2AFY2	-0.4295681	5.20E-04	RGD1561371	361844
POLRMT	0.61828196	5.28E-04		299604
NACAD	-0.5167076	5.31E-04		289786
PCSK5	0.6855457	5.31E-04	PC6|Pc5	116548
STK17B	0.713867	5.33E-04	Drak2	170904
ZFP36L2	0.57546765	5.41E-04		298765

RGD1305938	-0.5517365	5.43E-04		310362
POLH	0.64425135	5.48E-04		316235
RBM14	0.50478554	5.49E-04	CoAA	170900
PLAGL1	0.6053042	5.49E-04	Lot1|Zac1	25157
MARK3	0.3763424	5.61E-04		170577
PCMTD2	-0.57118213	5.62E-04	RGD1305684	311726
ZFP362	-0.6280068	5.62E-04	RGD1306498	297879
HCN1	0.86012423	5.63E-04		84390
GPR21	-0.44384855	5.65E-04	Gpr21	311911
GPR45	-0.5449812	5.67E-04		301372
DBP	-0.8268259	5.68E-04		24309
PCDHA6	-0.50182587	5.68E-04	rCNRv06	393088
EMD	0.42466643	5.68E-04		25437
GPR156	-1.0159905	5.73E-04	Gababl	260430
SLC48A1	-0.3968802	5.78E-04	HRG-1|Hrg1	300191
PRKCZ	-0.5678957	5.87E-04	14-3-3-zetaisoform|Pkcz|r14	25522
FTSJ3	0.51962984	6.00E-04		303608
NMT2	0.43841127	6.07E-04		291318
CHRAC1	-0.6103794	6.09E-04		315058
HSPA2	0.5357155	6.14E-04	Hspt70|Hst70	60460
TMEM86A	-0.7264606	6.16E-04	RGD1305749	308602
SLC40A1	1.4047453	6.16E-04	Fpn1|Slc11a3|Slc39a1	170840
SLC37A4	-0.5257178	6.18E-04	G6pt1	29573
LOC500877	-1.3927882	6.18E-04		500877
LINGO1	0.6413327	6.21E-04	Lrrn6a	315691
PRMT2	-0.62597674	6.24E-04	Hrmt1l1	499420
SLC28A3	-1.4889654	6.31E-04	Cnt3	140944
CAMK2N1	0.49420154	6.33E-04		287005
TMTC3	0.5425814	6.33E-04	RGD1306351	314785
TACR2	1.4816157	6.36E-04	Tac2r	25007
CITED4	-1.242663	6.37E-04	MRG-2|Mrg2	114491
PRKRA	-0.56349343	6.37E-04	RAX	311130
ABCB10	-0.54307234	6.38E-04		361439
SEC24D	0.83843863	6.48E-04		310843
LEPROT	-0.4193454	6.53E-04	Ob-Rgrp|Obrgrp	56766
PKLR	-0.9121864	6.56E-04	PK1|PKL|Pklg	24651
NOC2L	0.3833833	6.76E-04	RGD1309387	313777
TTLL1	-0.56456447	6.79E-04		362969
ZNRF2	-0.70722926	6.85E-04	Znrf1	362367
RIT2	-0.5996122	6.87E-04	Rin|Rit1	291713
CALHM2	-1.169739	6.87E-04	Fam26b|RGD1308276	294019
PNMAL2	-0.50676274	6.95E-04	RGD1560435	308393
XPO4	0.72588956	6.95E-04		290280

PACSIN3	-0.63019615	6.97E-04	SdpIII	311187
SYNGR3	-0.4901226	6.97E-04		302975
JRK	0.63231033	6.97E-04		315073
CALCRL	0.9037969	6.98E-04	CLR|Crlr|RATCRLR|RNCLR	25029
CYHR1	-0.8087888	6.99E-04	RGD1561328	100362155
KIF5B	0.42993933	6.99E-04	Khc	117550
NFKBIE	-0.7287623	7.15E-04	Slc35b2	316241
OSBPL2	-0.37741908	7.17E-04		296461
PLEKHA5	0.5339661	7.17E-04	Pepp2|TRS1	246237
PDE10A	0.9542575	7.17E-04	Pde10a3	63885
STRADA	-0.49474013	7.18E-04	Lyk5	303605
RASAL2	0.9257782	7.19E-04		304893
BEND7	1.0420662	7.35E-04	RGD1305898	361275
PHC1	0.48105434	7.35E-04		312690
IGF2BP2	1.0324925	7.49E-04	RGD1305614	303824
TPK1	-0.6448231	7.55E-04		680668
CHD6	-0.6291748	7.64E-04	CHD-6	311607
CHKA	0.42648286	7.71E-04	CK-R|Chk	29194
TMUB2	-0.3868561	7.73E-04	RGD1304758	303567
DOC2A	-0.58669204	7.80E-04		65031
USP10	0.53595936	7.80E-04		307905
CTC1	-0.45218167	7.83E-04	RGD1563106	303238
B3GNT2	-0.6569873	7.84E-04	B3gnt1	305571
CCKAR	-1.4610728	7.84E-04	Cck-ar	24889
OGDHL	-0.44704428	7.85E-04		290566
NKD1	-0.644302	7.96E-04		364952
PDE7B	0.7817121	8.00E-04		140929
PHF20	-0.40930417	8.04E-04	RGD1305020	311575
ZC3H6	-0.8585249	8.05E-04	Zc3h6	103690012
FSD1	-0.5281462	8.13E-04		301506
P2RY1	-0.8910657	8.19E-04	P2y|P2y1	25265
PURB	0.4570141	8.19E-04	pur-beta	498407
TMCC2	-0.6654097	8.26E-04	RGD1311960	305095
ILVBL	-0.6613915	8.34E-04		362843
CBFA2T3	0.8064325	8.46E-04		361431
TWIST1	1.1040758	8.48E-04	Twist	85489
PHLPP1	0.5765138	8.52E-04	Phlpp|Plekhe1|Scop	59265
PWP2	0.4914513	8.54E-04		690297
SHQ1	0.618061	8.56E-04	RGD1310610	297483
NCALD	-0.3158198	8.67E-04		553106
TIGAR	0.42375776	8.67E-04		502894
BYSL	0.44311565	8.71E-04		359727
MLF1	0.7294841	8.72E-04		310483

BHLHE23	1.2740167	8.75E-04	Bhlhb4|RGD1559760	499952
VAC14	-0.38587335	8.80E-04		307842
KCTD6	0.4704236	8.86E-04		305792
MAD2L2	-0.52181566	8.86E-04		313702
RNF4	0.43907994	8.86E-04		29274
TUSC5	1.3077588	8.86E-04	DSPB1	360576
D2HGDH	-0.7407282	9.03E-04	RGD1307976	301624
PPP1R21	-0.44658285	9.11E-04	RGD1565310	362697
PIGW	0.58660764	9.11E-04		378774
SH3BP1	0.7184306	9.11E-04		300067
FOPNL	-0.64248985	9.11E-04	RGD1305823	360461
RITA1	-0.6617135	9.16E-04	RGD1306772|Rita	288683
DHX15	0.37572557	9.16E-04		289693
TXNRD1	0.43897274	9.16E-04	Tr	58819
TRPM4	-0.7388621	9.16E-04	LTrpC-4|Mls2s	171143
WNT7A	-0.44404274	9.17E-04		114850
SLC24A4	0.65773785	9.17E-04	Nckx4	314396
RBFA	-0.6280929	9.22E-04	RGD1311910	307235
LIPA	-0.48053014	9.24E-04	Chole|Chole2|LAL|Lip1	25055
BACH1	0.8233174	9.38E-04		304127
APEX2	0.5453076	9.39E-04		317628
SYT9	-0.6798635	9.45E-04	Sytv	60564
PCSK7	0.42560545	9.45E-04	PC7	29606
DGCR8	0.3542616	9.58E-04		287954
ATP1A1	0.5371216	9.66E-04	Nkaa1b	24211
KLHL21	0.52049124	9.70E-04	RGD1305863	313743
DMTF1	0.74144053	9.78E-04		114485
PDK1	0.49384907	9.83E-04		116551
VLDLR	0.4947575	9.90E-04		25696
INPP5D	0.9800654	9.92E-04		54259
COASY	-0.5244441	9.95E-04		287711
AATK	-0.7821023	9.97E-04		690853
TFRC	0.6394503	0.001003031	Trfr	64678
IVNS1ABP	0.55952954	0.001011257		289089
FZD4	0.8261322	0.001014388		64558
MYOCD	1.4390517	0.001021537	Mycd	246297
P2RX2	-1.0103608	0.001063429	P2X2	114115
WNT3	-1.0721376	0.001075465	INT4	24882
NGLY1	-0.5888386	0.001078654		361014
DCAF4	-0.5240574	0.001085269	Wdr21	362762
SOX4	-0.666895	0.001087743		364712
CDH15	-1.0086491	0.00109044		361432
SHMT2	0.7363225	0.001096925		299857

NOP9	0.46830082	0.001098523	RGD1308396	290235
DIEXF	0.6520972	0.001115796	Def	305076
XPO1	0.64450747	0.001121369		85252
CHD1	0.70372075	0.001121369		308215
OSBPL8	0.42345697	0.001121503	RGD1561474	314824
FRMD3	-0.49199355	0.001123587		298141
IFT52	-0.48374653	0.001123977	RGD1311004	362265
MMP16	0.7201553	0.001137568	Mt3-mmp	65205
NTF3	-0.91430616	0.001137797		81737
UGCG	-0.408513	0.001153735		83626
PPIF	0.4912731	0.001161996	CyP-D|CypD|PPIase	282819
ACAP1	0.87039363	0.001164913	Centb1	287443
LANCL2	-0.40217626	0.001165692		362375
LOC100302465	1.327915	0.001170263		100302465
GPR176	-0.6699465	0.001175242	Gm1012|Gpr|RBU-15	117257
BORCS5	-0.97918296	0.00118038	Loh12cr1	362452
TEAD1	1.1423496	0.001182609	TEF-1	361630
TMCC3	0.6989743	0.001187061	RGD1307241	314751
CCDC82	-0.55604905	0.001195598		300359
KCNH3	-0.70602757	0.001198509	Bec1|Elk2	27150
WSCD2	-1.1164229	0.001199013	Wscd2-ps1	360824
EHD1	-0.48566034	0.001199013	RGD1306960	293692
BNIP3	-0.6796134	0.00120515		84480
STAMBP	-0.60814065	0.001211643	Amsh	171565
PTPDC1	0.50092864	0.001211643		291022
ANXA7	0.4737687	0.001220179		155423
NPY2R	1.174916	0.001229187		66024
TMEM106C	-0.50079596	0.001229815	RGD1311532	315286
BRINP3	-0.8265199	0.001230632	Fam5c	286901
PDCD6IP	0.46702114	0.001230632	AIP1|Alix|RGD1561176	501083
NUDT18	-0.6500521	0.001234377	RGD1311802	361068
SLC45A3	-1.2641804	0.001236445	RGD1309764	304785
TRAPPC3	-0.42669937	0.001236766		362599
HARS	0.37955672	0.001241341	Dnd1	307492
SFPQ	0.59208065	0.001242321		252855
SPRTN	0.60773844	0.001254553	RGD1559496	292101
LRRC16B	-0.5078315	0.001264088	Lrrc16b	361041
RAB9B	-0.56710654	0.001279833	RGD1562951	367915
OLFM4	0.9606124	0.001293636		290409
NUP93	0.45545802	0.001306387		291874
ING1	0.40621704	0.001317824	p33ING1b|p33ING1c	306626
KCNK3	0.85008407	0.001321135	Task-1|rTASK	29553
CYSRT1	-1.1947086	0.001322035	RGD1560880	499747

MMP15	0.5081275	0.00132987		291848
NXF1	0.5484511	0.001358358	Mex67h|Tap	59087
URI1	-0.58499944	0.001370479	RGD1310358	308537
WDSUB1	-0.56799155	0.001376847		362137
CEP295	0.6915539	0.001376847	RGD1311723	363018
PSTPIP2	0.85054135	0.001378867	RGD1563090	307248
FUCA2	-0.77564716	0.001382232		292485
SULT2B1	-0.75320095	0.001387261	ST2B1	292915
BCL2L13	0.55887043	0.001388408		312682
ASAP1	0.8193613	0.001390398	Ddef1	314961
EML5	0.90218073	0.001393962		444982
RNF6	0.40930188	0.00139603		304271
SERINC2	0.8296205	0.001399499	Tde2l	313057
NAA25	0.5468156	0.001402575	LRRGT00164|Mdm20|RGD13	360811
AGK	-0.46472198	0.001407379	RGD1562046	502749
TOR1AIP1	0.45769244	0.001441091	Lap1b|Lap1c	246314
ANKRD13D	-0.5459929	0.001462133	RGD1309156	361699
DIRC2	-0.5177993	0.001462133		303902
HPCAL4	-0.65064806	0.001465978	NVP-2|Nvjp2	50872
NPAP60	0.37747714	0.001465978	Nup50|Rtp60	25497
TNIP1	-0.5493757	0.001474619		363599
SPAST	-0.37951675	0.001488689	Spg4	362700
BCORL1	-0.75904447	0.001489755	RGD1566108	302810
HMGCR	0.49033245	0.001514448	3H3M	25675
MYLIP	0.5107507	0.001516362	MIR	306825
RNF112	-0.5731631	0.001517352	BFP|Bfb|Zfp179|Znf179	24916
PLA2G4A	1.1080424	0.001519433	Pla2c|Pla2g4|cPLA2	24653
RRAGA	0.5169129	0.00152748	Raga	117044
42796	-0.45689023	0.00153112	MARCH-II|RGD1306395	362849
FAM217B	-0.88913196	0.001552614	RGD1311952	311692
SYT3	-0.7124081	0.00156439	SIII	25731
ZFP672	0.51476556	0.00156439	Znf672	303165
IMPDH1	-0.459075	0.001573219	IMPD 1|IMPDH 1	362329
TVP23A	-0.81732684	0.001580614	Fam18a|RGD1566405	360467
KIFAP3	-0.34634274	0.001587511		289168
RAB27A	-0.86322165	0.001593067	ram	50645
RBM18	0.33643258	0.001593067		311902
GLUL	0.38647527	0.001595521	Glns	24957
PXN	0.6410701	0.001595521		360820
DEPDC1B	0.5622565	0.001595577		310074
CYB561D2	-0.57080746	0.001609138		363137
RGD1559896	-0.34047088	0.001613119		498967
CD55	-0.96448857	0.001613967	Daf|Daf1	64036

NDST3	1.1187375	0.001627725		295430
YPEL3	-0.76707417	0.001634564	RGD1564579	293491
DIS3	0.6484738	0.001654481	RGD1304646	306103
PLXND1	-0.66652536	0.001662025		312652
AHCYL2	0.5672737	0.001666295	RGD1564895	312192
LSM10	-0.5304686	0.001670468		366468
ZFP523	-0.43426538	0.001670468	Znf76	361809
BCL11A	-0.89537686	0.001678072		305589
CLK2	0.4075584	0.001678072		365842
GLI2	1.040282	0.001678072		304729
NCBP3	0.48621503	0.001684382	RGD1308139	360563
RAB3GAP2	0.56497294	0.001684382	RGD1311518	289350
ZFP295	0.62897	0.001685243	Znf295	304056
MAP3K7	0.41928765	0.001690861	Tak1	313121
NEUROD4	-1.0769848	0.001694129		288821
SYT4	0.5085085	0.001725132		64440
FNDC3A	0.80316854	0.001731631	Fndc3	306022
HTR7	0.8087953	0.001731631	5Ht7	65032
TAF1A	0.46075687	0.001742467		360893
ZMYND8	-0.4993775	0.001744596	Prkcbp1|spikar	296374
RQCD1	0.35330376	0.001749681	Rqcd1	301513
LDAH	-0.34138876	0.001750941	RGD1311648	313949
CYP39A1	-0.94485986	0.001756238		301264
ACPP	1.3829361	0.001760973	5'-NT|Acpp11|FRAP|Ppal|RN	56780
AZIN1	0.5310784	0.001783718	Oazi|Oazin	58961
SEMA3C	0.5066261	0.001806473		296787
CARNMT1	0.6264513	0.001806898	RGD1311863	293871
SLC27A3	-0.43738768	0.001808057		295219
GOLGA7B	0.6574997	0.001808057		309378
SIPA1L2	0.5657982	0.00181921	Sersap2|Spar2	361442
APLF	-0.669483	0.001822101	RGD1565557	500247
NFIX	-0.61413676	0.001822101	Nfix	81524
MRPL10	-0.44270423	0.001824196	L10mt|MRP-L10|MRP-L8	691075
ZBTB24	0.49677444	0.001824461	Bif1a|ZNF450	365590
SMARCD3	-0.60978127	0.001829849		296732
GALK2	-0.5638727	0.001834044		296117
SMARCA5	0.6474431	0.001834044		307766
TXNDC11	0.35675973	0.001842396	RGD1307538	302899
TBC1D9	-0.5926496	0.001843215	RGD1308221	304645
TPH1	1.3470098	0.001868024	Tph	24848
PNMA1	-1.1105555	0.001874523	MA1	170636
JAKMIP1	-0.7710171	0.001885091	Marlin-1|Marlin1	305434
ARPP21	0.4453368	0.001891512	Arpp-21|Ppp1r1c|Ppp1r1cl|F	363153

KCNMB2	0.95317215	0.001892463	Kcmb2	294961
MAP2K5	-0.37010694	0.001892602	Mek5	29568
STC2	0.84162474	0.001892602		63878
NKIRAS2	-0.44531527	0.00191021		287707
SLC20A1	0.6861232	0.00191021	PiT-1	81826
RNF128	0.62710315	0.001913067	RGD1566282	315911
PCDHA7	-0.47689474	0.00191786	rCNRv07	393089
SPOP	-0.542691	0.00194976		287643
TRPC1	-0.52421254	0.001959578	Trrp1	89821
MYO19	0.7242043	0.001980904	RGD1559961	497974
FBXO41	-0.82166547	0.001982358	RGD1566130	312504
GALE	-0.53963566	0.001982854		114860
NAGA	-0.41248217	0.00198457		315165
KCND1	-0.6402698	0.001986341	Kv4.1	116695
SERPINB8	1.3687408	0.002010378		288937
ZFP36	0.76764697	0.002015744	Tis11	79426
TBC1D8	-0.4443501	0.00201678		680133
PDZD8	0.5937467	0.002042684	Pdzk8	308000
ALYREF	0.62773097	0.002062709	Thoc4	690585
EXO1	0.76198125	0.002064636		305000
FADS6	-0.6694298	0.002069547	RGD1311950	303671
PTP4A3	-0.6268247	0.002074878		362930
PPEF1	-0.81774217	0.002097266		317498
NEIL1	-0.71396124	0.002099288		367090
PIK3R1	-0.64042646	0.002099413	PI3KA	25513
DARS2	-0.48116833	0.002103762	RGD1308286	304919
MED9	-0.56644887	0.002105041	RGD1563669	497914
BRIX1	0.48678407	0.002134174	Bxdc2|RGD1308508	294799
ETNK2	-0.7629403	0.002158593	EKI 2	360843
ADM	0.98095083	0.002187922	Ap|H39316|RATAP	25026
LOC688452	0.7606615	0.002189862	Spaca6	688452
SENP2	0.5625889	0.002194873	Axam	78973
CHAF1B	0.49415588	0.002200997		288242
API5	0.39791232	0.002205337		362170
RUNX1T1	-0.83150077	0.002217144	Cbfa2t1	362489
CARS2	-0.49256566	0.002218968	RGD1311612	361184
SIN3A	-0.43387488	0.002218968		363067
MBLAC2	-0.6480122	0.002231585	RGD1306703	365627
CAT	-0.39946076	0.002231585	CS1|Cas1|Cat01|Catl|Cs-1	24248
ESR1	1.1407906	0.002235185	ER-alpha|Esr|RNESTROR	24890
DDX51	0.43235165	0.002315611		304570
SFRP4	1.0897659	0.002315611		89803
NDRG4	-0.48663014	0.002326731	Bdm1|Ndr4|smap8	64457

INIP	0.49384215	0.002329927	RGD1307983	298032
FSCN3	1.01676	0.002329927		296947
GRIP2	-0.678192	0.002331911	Abp	171571
IGF1	1.2108998	0.002331911		24482
XKR4	-0.59722596	0.002337273	XRG4	297801
VAT1	-0.4805402	0.00235633		287721
ST6GALNAC6	-0.46399876	0.00235633	Siat7F	407765
SLC44A2	-0.44919485	0.00235633	RGD1309680	363024
SLC24A3	0.5326149	0.00235633	Nckx3	85267
ORC1	0.9550075	0.00235633	Orc1l	313479
NDST4	-1.3208786	0.00235929		362035
RNF219	0.35777017	0.002367857	RGD1564706	361088
MCM4	0.64696366	0.002370721	Mcmd4	29728
PCDHA4	-0.38769412	0.002405588	Cnr1	116741
RIT1	-0.36586645	0.00246091	RGD1559874	499652
RIIAD1	1.0830668	0.002471164		100270669
ZMYND10	-0.7166203	0.002474723		363139
MYBBP1A	0.6370758	0.002480002	DBP	60571
GTF3C5	-0.38180616	0.002491373		362095
ACTL6A	0.40495035	0.002514606	RGD1307747	361925
NKX3-1	1.2755096	0.002515555		305999
DISP2	-0.6134049	0.002556581		311324
ARFGAP2	0.383457	0.002566636	Zfp289	362162
NDEL1	0.4134144	0.002567926	NUDE2|Nudel	170845
SPRED1	0.8288227	0.002567926		296072
PELI3	-0.749179	0.002570655	RGD1305989	309157
CDO1	-0.601678	0.002577847		81718
SLC25A32	0.49625838	0.002578091	RGD1565789	315023
SLC6A8	0.570552	0.002578091	CHOT1|CHT1|CRT|CT1	50690
ROR2	0.6379546	0.002579488		306782
NFXL1	0.7204357	0.002600309	RGD1359201	289595
NPLOC4	0.5224384	0.002606698	Npl4	140639
SLC25A42	-0.4402367	0.002609801		689414
MFAP2	-0.44396982	0.002639372		313662
TTC19	0.5083155	0.002639372	RGD1311797	691506
GPATCH2	0.6533226	0.002639372	Gpatc2	289362
LEMD3	0.40933073	0.002642271	RGD1561001	680066
RFXAP	0.61888963	0.002650466		499617
MCOLN3	1.2846128	0.002662769		308022
PLPPR4	0.4323068	0.002678223	Lppr4|Prg-1|Prg1	295401
VPS37D	-0.67426366	0.002682377		687208
ASUN	0.42108116	0.002704072		690728
PDK2	-0.44153845	0.002713821		81530

ZFP395	-0.49914	0.002722246		305972
CLDN3	-1.327775	0.002735947		65130
SV2C	1.3235396	0.002735947		29643
MAFF	0.6750922	0.002736387		366960
FKBP1A	0.31115192	0.002739557	FKBP12|Fkbp2	25639
ITPR3	1.1131233	0.002747432	IP3R3|IP3R3X	25679
CYP11B3	1.2890716	0.002758251	CYPXIB3	353498
ZBTB16	-1.1543972	0.002766361	Lx|Plzf|Zfp145|Znf145	353227
BPHL	-0.57253987	0.002774489		361239
HNRNPF	0.6241387	0.002774495	Hnrpf	64200
ZFP18	0.42917475	0.002779537	Zfp535|Zkscan6|Znf18	303226
HPN	1.3208531	0.002788		29135
B4GALNT4	-0.4399508	0.002813124	RGD1310052	309105
HERC3	-0.5670623	0.002856638		362377
SLC30A10	0.7927553	0.002856638	RGD1305098	289353
SLC35B4	-0.37633476	0.002863239		296969
ATAD3A	0.32929096	0.002863239	Atad3	298682
TBCK	-0.3988576	0.002872885	RGD1307816|Tbckl	295446
NOL3	-0.4864958	0.002873274	Arc	85383
NIFK	0.5546444	0.002931919	Mki67ip	246042
OPRK1	-1.2661084	0.002945226		29335
LRRC75B	-0.89601713	0.002945226	Fam211b	100365744
TBC1D25	-0.5088058	0.002945226	Oatl1|RGD1559711	302552
EEA1	0.5295778	0.002949305		314764
BRINP1	0.58867437	0.002951721	Dbc1|Dbccr1	140610
SPTLC1	0.39000693	0.00295267	Lcb1|RGD1306617|Spt1	361213
ARL4D	0.7306127	0.00295267	Arf4l	303559
HGS	0.36550793	0.002959091	Hrs	56084
FAM26F	1.3035073	0.002999965	RGD1304835	294430
ANO6	0.74228007	0.003006419	Tmem16f	315272
LOC361346	0.6765565	0.003009195	Las2	361346
RIMS4	0.9382637	0.003045795	Rim4gamma	266976
PSMB11	1.047436	0.003048026	RGD1308773	290206
SEC24B	0.36643368	0.003058924		295461
NMBR	1.0635699	0.00308967	NMB-R	25264
HNRNPU	0.44230294	0.003123493	Hnrpu|SN1	117280
RBPJ	0.64910966	0.003143098		679028
WDR6	-0.50963014	0.003184359		301007
SLC12A6	-0.7204348	0.00319381	Kcc3	691209
CIR1	-0.8313745	0.003194235	1700023b02rik|Cir|RGD1309	362149
ATRAID	-0.5178097	0.003194644	RGD1311605	298841
DCAKD	-0.30305466	0.003211073		360639
DND1	1.0508786	0.003215087		679841

LCK	-0.7098316	0.003231213	Lck1|Lcktkr	313050
NR1D1	-0.4220045	0.003231213	REV-ERBAALPHA	252917
RSRP1	-0.39317167	0.003245802	RGD1359529	362626
FBL	0.6051908	0.003247209		292747
BBS2	-0.5848547	0.003267711		113948
ZFP639	0.4032127	0.003267711	Znf639	683504
MRPS2	0.35226396	0.003272506		362094
CDK18	-1.0914624	0.003274151	PCTAIRE3|Pctk3	289019
FAM222B	0.5397364	0.003284344	RGD1566149	497960
DYNC1I1	-0.30571437	0.003284492	Dnci1|Dncic1	29564
ENGASE	-0.6188538	0.003294754	RGD1310868	303702
PCBP4	-0.31813145	0.003294754		363133
CHSY1	0.3295991	0.003300143		292999
SCN4A	-1.1988186	0.003331173	NCHVS|Nav1.4|microl	25722
ZFP800	0.69300103	0.003331173	RGD1560157	500057
TMEM87B	0.45328468	0.003332206	RGD1307907	362212
PPP1R10	0.43675312	0.003333753	Fb19|Pnuts	65045
ZFYVE19	-0.47516018	0.003340885		499871
FN3K	-1.18426	0.003346953	Fnsk	498034
MYCN	-0.46250296	0.003346953	N-myc|Nmuc1|Nmyc	298894
MON2	0.66772544	0.003356609	RGD1308808	314894
EMILIN3	1.0215374	0.003366859		362262
SLC30A2	-1.2455347	0.003374921	ZnT-2|Znt2	25362
PURG	0.5889565	0.003383831		361162
PTGIR	1.2722929	0.003413173		292661
FSTL5	-0.35508668	0.003419268		365823
FCHO1	-0.44358185	0.003452883		290639
CHMP1A	-0.41559383	0.003452883	Pcoln3	365024
EMP3	1.2313905	0.003452986		81505
RPS6KA6	0.47431427	0.00346415	RGD1560817	317203
NPAS2	0.5455637	0.00346415		316351
SHD	-0.56852055	0.00346793		316507
ELOVL4	0.3928217	0.003474993		315851
HMGCL	-0.47724962	0.003477131		79238
JUP	-0.6522347	0.003483449		81679
MFN1	0.42748147	0.003500277	Fzo1b	192647
SLC2A8	-0.49465877	0.003501701	Glut8	85256
ZDHHC20	0.5142524	0.003510928	RGD1305755	305923
CCDC32	-0.6085224	0.003520041	Gm631	296081
ZPBP2	-1.0296645	0.003535947		363676
FADD	0.8071368	0.003547048	Mort1	266610
FBXO30	-0.5166577	0.003554638		308283
MTPAP	0.4852335	0.003593015	Papd1	307050

FOXP2	-0.6970624	0.003596837	RGD1559697	500037
LOC100125367	0.70325047	0.003596837	C1orf74	100125367
OSCAR	1.1101156	0.003609305	RGD1559897	292537
KCNA4	0.64054894	0.003625348	KCHAN|Kv1.4|Kv4|RHK1|RK3	25469
FAM129C	-0.9176881	0.00363949		498604
NAT14	-0.5717621	0.00363949	RGD1565991|Zfp628	361500
SMIM17	-0.68858564	0.003641049	RGD1559613	499067
AKAP9	-0.52254903	0.003653498	CG-NAP|Gisp|Yotiao	246150
ORC2	0.6520922	0.003659118	Orc2l	301430
BCKDHA	-0.4117469	0.003673959	BCKDA|E1a	25244
RNF139	0.37509334	0.003691356	TRC8	315000
DNAAF5	0.6442604	0.003704938	Heatr2	304332
APBB3	-0.45330966	0.003707689	Fe65l2	117026
TYMS	0.58960056	0.003712491		29261
PXYLP1	-0.49903175	0.003724332	Acpl2	315939
DTX2	0.49222773	0.003763813		304591
ZFAND6	-0.3773252	0.003771226	Za20d3|zgc:101121	293067
REEP2	-0.35044065	0.003773972		682105
N4BP3	-0.7732296	0.003786323	C330016o10rik|RGD1308356	303112
CCDC28B	-0.6469193	0.003786323		682445
RPS6KB2	0.38501665	0.003795693		361696
RGS14	-0.69307476	0.003797186		114705
PUS7	0.67190504	0.003797186	RGD1307054	296751
RANGAP1	0.41167435	0.003804426		362965
LRCH1	0.58920455	0.003804426		502020
TENM3	-0.6950083	0.003808736	Odz3	306451
TFAP4	-0.67507505	0.003827042	Tcfap4	360482
TBK1	0.40808332	0.003830434		299827
TMEM223	-0.5691301	0.003839845		690285
IFT81	-0.6005608	0.003870222	Cdv-1|Cdv1	373066
ITSN1	0.6095012	0.003875809	Itsn	29491
FOXO3	-0.89672095	0.003884692	Fkhrl1|Foxo3a	294515
PPP4R3A	0.38124272	0.003885117	RGD1309059|Smek1	314388
CSRP2	-0.6035509	0.003912169	SmLIM	29317
RGD1308147	0.37867263	0.003940911		307008
WNT9A	-0.85681874	0.003952992		287357
POLR3E	0.34231988	0.003956293	RGD1308086	361640
TBC1D14	-0.4229025	0.003971899	SRF-2|UR-NR#2	360956
NCS1	0.49619532	0.003984428	Freq	65153
KTI12	0.5730082	0.003994137		685656
NRSN2	-0.8531885	0.003997315		689978
CISD2	0.42032087	0.004040704	RGD1566242	295457
RHOQ	0.6162784	0.004042533	Tc10	85428

SDE2	0.3299553	0.004044133	RGD1305572	289315
PHOSPHO2	0.3698227	0.004055211		295663
BNIP3L	-0.42774191	0.004081985	Nix	140923
RBBP7	0.4944776	0.004090612		83712
GRIN3B	0.5486226	0.004097416	GluN3B|NMDAR3B|NR3B	170796
GEMIN5	0.62930375	0.004097416		691231
CDC16	0.28580996	0.004109758		290875
GPATCH11	0.39006427	0.004110932	Ccdc75|RGD1311424	362685
SLC5A4	-1.2244954	0.00411264	Slc5a4a	294341
RGD1564804	-0.6460024	0.004115207		313551
FN3KRP	-0.46802858	0.004123667	RGD1304570	303755
UBE2F	0.41229182	0.004123667	RGD1307608	363284
AMZ2	-0.40870693	0.004130729	RGD1304846	360650
SLC9B2	-0.7989277	0.004135514	Nhedc2	679958
SUN1	0.59840167	0.004141931	Unc84a	360773
SLC25A39	-0.4051778	0.004168978	RGD1306193	360636
ATF1	0.51113796	0.004168978	Atf-1	315305
SPATA5L1	0.5796718	0.004168978		691729
IDH1	-0.4401083	0.004169217		24479
UBE2D3	0.3849438	0.004222871		81920
EML1	-0.66444993	0.004228776		362783
PRPF18	-0.37852702	0.004251674	KCRF|Pprf18	171552
ACSL1	0.34920642	0.004251674	ACS|Acas|COAA|Facl2	25288
LAMC1	0.7354476	0.004274136	B2e	117036
NAPEPLD	0.77145904	0.004277008	NAPE-PLD	296757
DDX52	0.5531235	0.004299835	Rok1	85432
ULK2	-0.33573863	0.004315719		303206
SH3GLB1	0.4935133	0.004339478		292156
CEBPG	0.5350657	0.004339478	C/EBP|CEBPRNA	25301
ABHD8	-0.7104634	0.004342752		306338
SYT6	0.79413813	0.004346998		60565
RECK	0.7344724	0.004355526		313488
MPLKIP	0.4674159	0.004360512		684996
PCDHA10	-0.61110485	0.004377054	rCNRv10	116778
ELK3	0.551439	0.004377054		362871
NEPRO	0.6178104	0.004377054	RGD1311458	303948
SLC5A3	1.0699618	0.004377054	Smit	114507
TUBB4B	0.28440395	0.004377083	Tubb2|Tubb2c	296554
PLEKHA4	-0.593785	0.004413514		308584
MCMBP	0.49211955	0.004427683	MCM-BP|RGD1306730	309009
TJP3	-0.85026395	0.00444724		314640
UXS1	0.37348494	0.004454902	UGD	246232
VWC2	0.865909	0.004454902		501231

MEPCE	-0.39203325	0.004465452		304361
CSTF2	0.29538757	0.004491416		683927
FUNDC1	-0.5906039	0.004509994		363442
CUL3	0.36666733	0.004511394		301555
ALKBH1	0.41555202	0.004555387	Alkbh	362766
CEP85L	0.7712387	0.004556595		100365935
THBS2	1.1592374	0.004579172	TSP-2	292406
CCDC71	-0.5524986	0.0045851		498678
PRTN3	-0.91662306	0.004593573		314615
TUBA4A	0.42560843	0.004598877	Tuba4	316531
TSPAN17	-0.51210874	0.004600765	Fbxo23|GHB-R	306771
TMEM202	1.0555767	0.004626515		691306
RHOA	0.49570227	0.004637632	Arha|Arha2	117273
GNE	0.51726013	0.004663155	Uae1	114711
ALKBH2	-0.8614533	0.004668592	RGD1306377	304578
KDM4A	-0.49764067	0.004688337	Jmjd2a	313539
HNRNPH1	0.46152624	0.004688713	Hnrph|Hnrph1	140931
PAX1	1.1155308	0.00469122	Pax-1	311505
TUBG1	-0.47636124	0.004773877		252921
NFYA	0.48423356	0.004798219	CBF-A	29508
PRKAA1	0.3957278	0.004844106	AMPKalpha1	65248
POLD1	0.49632335	0.004845094		59294
CNNM2	-0.65561235	0.004852129		294014
AKAP5	-0.8019565	0.00488042	AKAP150|Akap79|P150	171026
RNF145	-0.44215265	0.004890411	RGD1309561	287212
COMMD7	-0.49026376	0.004907491		296285
GAR1	0.56295294	0.004907491	Nola1	499709
NTRK3	0.7086573	0.004907491	trkC	29613
SMYD5	-0.3803803	0.004916756		312503
PCDHA5	-0.5710929	0.004936068	rCNRv05	393087
FIG4	-0.4024622	0.004944686	Kiaa0274|RGD1311375|Sac3	309855
42802	-0.4099952	0.00495365	Mir	312656
MZF1	-0.408548	0.004959129	Zfp98	361508
PPP1R1A	0.6349888	0.004966796		58977
PEAK1	1.2385699	0.00496826	RGD1312026	315686
RNF167	-0.35393977	0.00497668	RGD1305972	360554
PPP3CC	-0.38419008	0.004986884		171378
TRPC4	-0.5223144	0.005011922	Trp4|Trrp4	84494
NAP1L3	-0.72873604	0.005018402		170914
CHST10	0.52101976	0.005029481	Hnk-1st	140568
RPAP2	-0.4148779	0.005081292	RGD1309034	305120
F2R	0.36301935	0.005097748	Par1|TRGPC	25439
TTC14	0.639218	0.005097748		310314

GAS7	-0.5019012	0.00510623		85246
SDK2	1.196949	0.005116664		360652
SPDYA	-0.76542056	0.005124109	Gs4|Lm23|Spdy1|Spy1	192209
BOK	-0.701874	0.005136154	Bok-BH3	29884
EMC9	-0.8998065	0.005136166	Fam158a|RGD1308113	290224
TMPO	0.32697394	0.005138164	LAP2	25359
KCNK1	0.36667544	0.005139695	Twik	59324
TERF1	0.5834361	0.005147464		297758
TGIF1	0.6937128	0.005147464	Tfig|Tgif	316742
HPGD	1.1811209	0.005147464		79242
FJX1	0.5376544	0.005156112		366140
CDC25B	-0.48590094	0.005168988		171103
SVOP	-0.60297906	0.005182482		171442
HEMK1	-0.5954756	0.005182482	RGD1308293	300989
ZDHHC5	0.6428169	0.005216695	DHHC-5	362156
RCOR1	0.44634447	0.005250085	RGD1305743	102554884
IL1RL2	1.1775132	0.005268157		171106
NASP	0.58709365	0.005275937		298441
FBXL6	-0.5333863	0.005339098		362941
MED8	0.45743755	0.005354017		362575
SOBP	-0.631317	0.005394171	RGD1560479|Sobpl|jcx1	309860
LOC361635	-0.35061666	0.005394371		361635
CDCA4	0.38979167	0.005422936		500727
FZR1	-0.509077	0.005477336		314642
MELTF	-1.0656476	0.005504429	Magea1|Mfi2	288038
FAM220A	-0.41932482	0.005504429	Sipar	498145
CCDC93	0.5447762	0.005507648		304743
TFDP1	0.34993988	0.005546859		361178
GLRX	-0.48387155	0.005596032	Glrx1|Grx	64045
KIF3B	-0.31642193	0.005596032		296284
ASCC3	0.49893677	0.005650464	Helic1	309887
PPFIA4	-0.76504517	0.005665115		140592
ISG20L2	0.7826963	0.005676423		361977
TNRC6B	-0.6648719	0.005709778	Cbl27	192178
TNFRSF25	1.0643183	0.005733798		500592
TMEM141	-0.6813307	0.005735843	RGD1561492	499755
WNT4	0.8826062	0.005740115		84426
CLN5	0.60114914	0.005746855		306128
WDR3	0.3375209	0.005779792		310720
ABTB1	-0.6536297	0.005859064		297432
ERI2	0.51821464	0.005869466	Exod1	691484
PIGM	0.56453097	0.005895754	GPI-MT-I	79112
CORO1C	0.5309538	0.005907807	RGD1564490	501841

PKDCC	0.49999848	0.005909331	RGD1311939|Sgk493	313860
RGD1307315	-0.4464015	0.00591617		362793
MSANTD3	0.42342773	0.005918904	RGD1308165	362516
EPHA2	1.0286369	0.005919148		366492
FBXO31	-0.6204082	0.005923453	RGD1561069	498959
MAGED2	-0.35522595	0.005938329		113947
AKAP12	0.4540927	0.005938329	AKAP12A|AKAP12B|AKAP12(	83425
ACVR1B	-0.52200156	0.005945652	Skr2	29381
CNTN3	-0.86453986	0.005964303	BIG-1|Pang	54279
YPEL2	-0.9454961	0.00598012	RGD1311564	360590
MSL3L2	0.7059998	0.005988806	RGD1308699	309790
PRR13	-0.40907007	0.006000093	RGD1307129	363004
FAM129B	0.54152995	0.006012125	RGD1307018	362115
NR2F6	0.46301594	0.006019252		245980
SLC35F5	-0.36386016	0.006063604		288993
NOV	-0.5798898	0.006083168		81526
ASIC1	-0.42842707	0.006100501	Accn2	79123
SVBP	-0.8070299	0.006107451	Ccdc23|RGD1311232	362578
NABP1	0.7346499	0.006111566	Obfc2a|RGD1306658	363227
TMEM205	-0.6299742	0.006129767	RGD1563250	300441
NAT10	0.37204736	0.006133545	RGD1306717	311257
CANT1	-0.5045032	0.006144266	srapy	246272
RNF180	-0.37024853	0.006163667		685384
CISD3	-0.6918637	0.006166389	Mel13|RGD1559720	287661
IPO7	0.39745855	0.006166389		308939
DFNB31	0.5800239	0.006166389	Cip98|Whrn	313255
PACSIN2	0.63012505	0.006166389	SdpII	124461
EPRS	0.5938935	0.006239232		289352
AP4B1	-0.3710514	0.006244707		310746
TRANK1	0.74175256	0.006244707	RGD1563130	316022
AGFG2	-0.5693147	0.006261388	Hrbl	304375
SAMHD1	0.49654856	0.006312627		311580
SAMD5	0.8477639	0.006338316	RGD1311012	365038
LIG4	0.66022676	0.006354985		290907
LRRFIP1	0.3652048	0.006383461		367314
RAB2B	-0.3584162	0.006421351		305853
TAF2	0.30742243	0.006444161	TAFIIB	170844
SYT12	0.75857925	0.006454528	Srg1	191595
ATG4C	-0.42357612	0.006478442		313391
VGLL4	0.35816586	0.006478442		297523
AQP11	-0.61925507	0.006514886		286758
NARS	0.44664752	0.006528283	LRRGT00113	291556
IGFBPL1	-0.6693971	0.006588765		366366

INO80C	-0.36147293	0.006594931	RGD1310199	291737
TACR1	-1.0150286	0.006642334	Tac1r	24807
LENG8	0.5379686	0.006642334		361506
FRAS1	1.1956491	0.006642334		289486
NRAP	-0.94512296	0.006742248		307982
RAI2	-0.4963332	0.006761554	RGD1560139	501555
ATP5S	-0.8233238	0.006810316		362749
CNR1	-0.59843177	0.006857776	SKR6R	25248
FGF8	1.2183199	0.00687262		29349
PEX14	-0.43377188	0.006914046		64460
CDC42EP2	-0.8827879	0.006927021		309175
KCTD15	-0.39976555	0.006962657		499129
BAMBI	-0.64160264	0.006993972		83837
TIMP4	0.61468256	0.006993972		680130
TARBP2	-0.3733423	0.007031335		363006
HMG20A	-0.3441888	0.007074592	RGD1564760	315689
TMEM114	1.1656243	0.007099612	RGD1560593	501675
KCNE2	-0.7082734	0.007102726	Mirp1	171138
BEND5	-0.6119894	0.007102726		362564
MMS19	-0.3799196	0.007102726	Mms19l	171124
PARD6B	0.4408882	0.007102726		362279
RGD1310769	-0.47779393	0.007161525		299207
RBAK	0.40330344	0.007161525	RGD1562875	288489
WDR4	0.66925246	0.007161525		690032
NECAB3	-0.8259294	0.007169013	Apba2bp	311562
MRPL34	-0.7356181	0.007183372		290632
DTX3	-0.41688272	0.00719039	RGD1566181	500847
FRY	-0.8267952	0.007203703	RGD1307034	304244
LDB1	-0.3872721	0.007323738		309447
NR0B1	1.0694661	0.007323738	Ahch|DAX-1	58850
DEAF1	-0.55294883	0.007333743	NUDR	83632
TARDBP	0.3015849	0.007337708	Tdp-43	298648
ATG10	-0.7369567	0.007339263		688555
ARL4A	0.5981951	0.007339263	Arl4	29308
TDRD6	-1.1833401	0.007401314		316254
TMEM106B	-0.30082932	0.007401314	LRRGT00101	312132
PCDH7	0.76379704	0.007401314		360942
CDK5RAP1	-0.53716445	0.007436976		252827
SNRPA	0.3253762	0.007436976		292729
SLC43A1	0.850067	0.007438363		311168
TMEM178A	0.5657237	0.007500956	Tmem178	362691
TES	0.5907334	0.007521784		500040
TRAPPC4	-0.64285696	0.007616847	Sbdn	367073

TOP1MT	-0.59850335	0.007637879		300029
CBLN1	-0.61230785	0.007648109		498922
HIBCH	-0.42652848	0.007669291		301384
CRY2	0.55243105	0.00776325		170917
CBLN4	-0.76772314	0.007824593		499947
FRA10AC1	-0.6739591	0.007824593	RGD1309482	365458
LRRC7	0.74293244	0.007852638		117284
NHLH2	-0.99175644	0.007871		295327
PHYHD1	0.6154232	0.007871		296621
ATXN2L	0.67690605	0.007871434	RGD1565868	361649
WDR5	0.3201417	0.007872803		362093
VARS2	-0.37360045	0.007873848	Vars2l|Varsl	309596
HRH1	0.7724292	0.007873848	Hisr	24448
PLXNA2	0.79055667	0.007896097		289392
RNGTT	-0.30786905	0.007917888		313131
ATG13	-0.5128015	0.007935271	Harbi1|Harbi1l|RGD1310685	362164
DEF6	-1.148882	0.007943125		309642
SLC9A5	0.49069187	0.007967993	Nhe5	192215
TMEM55B	-0.39198777	0.008072576	RGD1307475	364298
SARS	0.39547387	0.008072576	Sars1	266975
OLIG2	0.36800122	0.008075546		304103
BCOR	0.44199145	0.008079262	RGD1562735	317346
MFSD3	-0.58512545	0.008091276		500899
IWS1	0.46569702	0.008118515	RGD1304762	291705
ST8SIA4	-0.7798197	0.008123681	Siat8d	116696
RBM46	-0.7862903	0.008132804	RGD1560205	310548
RGD1311188	0.5532214	0.008146983		315088
CYR61	0.95583934	0.008146983		83476
SGMS1	0.44758293	0.008148881	Mob|Tmem23	353229
SLCO1C1	0.6127385	0.008177418	Bsat1|Oatp14|Slc21a14	84511
DPF1	-0.58634126	0.008184612	Neud4	50545
RIOK3	0.3015605	0.008250138		361293
PTPRH	-0.9853315	0.008254619	Bem2	171125
DUOX1	1.0812548	0.008254619		266807
RGS9	-0.7473781	0.008289885		29481
COQ10A	-0.4284515	0.008343717	RGD1562447	362810
ANKEF1	1.2020665	0.008350425	Ankrd5	296184
PJA2	-0.35182375	0.008373673	Neurodap1|praja2	192256
ST3GAL2	-0.54038936	0.008385118	Siat4b|Siat5	64442
C1QL1	0.43491355	0.008396974		363686
SMOX	0.34669903	0.008435047		308652
PIH1D1	-0.47758472	0.008503411	RGD1309809	292898
WLS	0.37792286	0.008532126	Gpr177	362065

TMF1	0.63926774	0.008532126		114206
TTC33	-0.42918146	0.008553218	RGD1564042	294774
KEAP1	-0.48125336	0.008556129	Inrf2	117519
RASGRF1	-0.44856116	0.008556129		192213
KPNB1	0.56883645	0.008556129	Impnb	24917
CUL4A	-0.3229395	0.008583327	RGD1563853	361181
GALNT3	-0.77247727	0.008656305		366061
PRPSAP2	-0.40071207	0.008660885	Pap41	117272
CCDC40	-0.65257496	0.008794177		287867
CFAP97	-0.57325876	0.008794177	RGD1307325	306469
NFKB1	0.59376323	0.008794177	EBP-1|NF-kB	81736
ZFP189	0.6014152	0.008794177		313219
EFNA5	0.8253024	0.008794177	Lerk7	116683
IL11	-0.8465593	0.008835731	Il-11	171040
BANP	-0.35008252	0.008862481		292064
FSCN1	0.5502662	0.008885613	Fascin	683788
ELAC2	0.37857094	0.00893575		282826
LMBR1L	-0.5199525	0.009033621	RGD1309631	300215
HERPUD2	-0.53365934	0.009096939	RGD1307343	300463
MAPK1IP1	-0.50829536	0.009096939	RGD1565431	499280
GNL3	0.5454993	0.009097274	Ns	290556
KCNK13	1.0015669	0.009097274	THIK-1|prdx1	64120
KLF13	-0.5957496	0.009102651	RGD1565099	499171
LZTFL1	-0.57112455	0.009116273		316102
XKR7	1.15487	0.009132666		311549
BTG4	-1.0542605	0.009133044	SCIR-27	315650
FLOT1	-0.57301366	0.009232132	Rareg	64665
WDR77	0.35151115	0.009239938	Ac2-269|MEP-50|RGD13104	310769
NPY	0.94925565	0.009372319	NPY02|RATNPY|RATNPY02	24604
PPP1R1B	0.43043384	0.009427398	Darpp-32|Darpp32	360616
ELF3	-1.1831183	0.009427593		304815
KATNB1	-0.4570654	0.009427593		291852
SGCA	0.9726473	0.009429377		303468
ZC3H11A	0.73905	0.009568936	RGD1308290	360845
KCNK5	0.898048	0.009568936	TASK2	364241
CDK8	0.34470317	0.009589051	RGD1560888	498140
ASCC1	-0.4827921	0.009596734	Asc-1|CGI-18	294512
CFB	0.99190015	0.009596734	Bf|Da1-24	294257
CDK2AP2	-0.44704607	0.009633761		688405
USP13	0.3351111	0.009750959		310306
BZW1	0.4769006	0.009750959		363232
RASA1	0.46877527	0.009786127	GAPX|Rasa	25676
MAK16	0.60697174	0.0098085	RGD1311297|Rbm13	306526

INTS12	0.40871048	0.009844609	Phf22	295448
RAB27B	0.7839486	0.009873074		84590
MSRB1	-0.37079212	0.00987861	Sepx1|selX	685059
MAP3K12	-0.36958146	0.009950466	DLK|MUK|PK|Zpk	25579
PDE3B	0.55044544	0.009950466		29516
VAV3	0.66234994	0.009977018	RGD1565941	295378
TMPRSS6	1.0566071	0.009998485		315388
HSCB	-0.53802866	0.010015708	RGD1311005	360826
TTC39C	0.5109129	0.010026858		686179
NOTCH4	-0.5388284	0.010029635		406162
RPS6KA3	0.7044436	0.010052121	RGD1563860	501560
GNA11	0.5249587	0.010080943		81662
PVRL1	0.6996072	0.010245699	HveC|Pvrl1|nectin-1	192183
PBX1	-0.7391616	0.010258959		304947
TBP	0.43738207	0.010327858	TFIID	117526
TMEM229B	-0.5321619	0.010432375	RGD1562622	503035
HINT3	-0.47867116	0.010432375	HINT-3|HINT-4|Hint4	246769
MET	0.7387908	0.01045371	Hgfr	24553
HBP1	-0.462579	0.010578232	Hmgb1	27080
BMS1	0.4483293	0.01058447	Bms1l	362426
POLDIP2	-0.2770717	0.01068762		287544
CDK12	0.72352934	0.01068762	Crk7|Crkrs|Pksc	192350
GLDN	0.7637601	0.010722259	Colm|Crgl2	315675
SDHC	-0.27536422	0.010724688		289217
AFAP1L1	0.7564462	0.010724688	RGD1311580	291565
BFAR	-0.32794145	0.010738542		304709
KMT5A	0.734331	0.010748866	Pr-set7|RGD1305893|Setd8	689820
ERBB3	0.7934279	0.010816959	nuc-ErbB3	29496
RAB43	0.8154377	0.010816959		500249
MYBL1	0.7658934	0.010897748		297783
JAKMIP3	-0.56649476	0.010920779	Necc2|RGD1307177	365380
PHRF1	0.45779696	0.010951469		245925
MRVI1	1.1131667	0.010966362		308899
COMTD1	-0.38202608	0.010984688		305685
RIOK2	0.51462376	0.010996142		308201
PCGF5	0.7161094	0.011064585		681178
PRRT2	-0.5121309	0.011066943	DSPB3|RGD1564195	361651
HDAC9	-0.72362775	0.011070985	RGD1310748|RGD1563092	687001
XRCC5	-0.4033135	0.011080731	Ku80|Kup80	363247
HPCAL1	-0.5157591	0.011088899	NVP-3|Nvp3|VILIP-3	50871
BMF	-0.83336586	0.011220546		246142
DDX39A	0.40229562	0.011228486	Ddx39|Ddxl	89827
LMO4	-0.76822114	0.011260265		362051

DDX56	0.33297938	0.011277332	Ddx21|RH-II/Gu	289780
PTBP1	0.5902062	0.01129836	PTB1|Ptb|Pybp|Pybp1|Pybp:	29497
METTL18	0.68845814	0.011364142	RGD1306783	304928
GOLPH3L	-0.35808986	0.011370461		310669
SRPRA	0.31458172	0.011376195	Srpr	315548
TRPC3	-0.5177324	0.011378234	TrpC3c|Trrp3	60395
ANKRD34C	1.1576787	0.011426149	RGD1305403	300889
CCDC148	-0.6401252	0.011467871	RGD1561169	311051
G3BP1	0.29962832	0.011478704	G3bp|RGD1310666	171092
HSPB11	-0.645847	0.011492399		685284
SLC19A2	0.44552222	0.011543294		289175
TRMT12	0.47362167	0.011628785	RGD1307127	314999
GRIP1	0.5774581	0.011628785		84016
FNDC1	0.82207674	0.011628785		308099
SLC39A6	0.297998	0.011633094		291733
DCAF10	0.5334191	0.011633094	RGD1304730|Wdr32	313242
DDI2	0.62694544	0.011633094	RGD1311815	313668
BANK1	-1.1293828	0.01166602	LRRGT00104	365948
PNKD	-0.36677125	0.011666717	Mr-1	100188944
SNX16	0.35181302	0.011668363		64088
LOC363337	0.9969346	0.011669235		363337
ANAPC7	-0.29959714	0.011677975		304490
CDV3	0.5922145	0.011718756		315970
ARHGAP25	0.9579346	0.011726267	RGD1562105	500246
PLOD3	0.3947694	0.011803632		288583
TEKT2	-0.93230605	0.011809778		298532
SRRM3	-0.4555189	0.011809778	RGD1307391	685890
MOSPD3	-0.51183414	0.011891845		288557
RAD51	0.44940814	0.01191205	RGD1563603	499870
PARD6A	-0.63022274	0.011914798	Par-6a|Par6a	307799
SMIM20	-0.5709944	0.011914798	RGD1565192	501923
GTF2IRD1	-0.46814457	0.011914798	Gtf3	246770
ARHGEF7	0.43864736	0.011914798	P85spr|Pak3bp	114559
LFNG	0.44454327	0.011976814		170905
RGD1309028	-0.6242827	0.012039031		299265
CASP9	-0.4441282	0.012155866	Apaf3|Casp-9-CTD|Casp9_v1	58918
ADAP1	-0.4007203	0.012155866	Centa1|p42IP4	171097
PI16	-0.7531638	0.012169292		294312
TCEAL1	-0.6499034	0.012176254		302593
OGFOD1	0.5635196	0.012176254	RGD1308848	307657
SEMA6B	-0.58345705	0.012255387		84609
ZFP9	-0.5530713	0.012271681		100158232
PAFAH1B3	-0.60000294	0.012316116		114113

PTP4A2	0.5444053	0.012316116		85237
CLCN5	0.99063647	0.012317729	CLC5	25749
CRAT	-0.43266946	0.012332224		311849
NUDT16	-0.59488744	0.012413353	RGD1311387	363129
CELF6	-0.35775325	0.012423826	Brunol6	300758
CDK2	0.42351413	0.012472671		362817
FAM227A	-0.5646193	0.012541516	RGD1305939	300074
RAD18	0.48092374	0.012632669		362412
HSPA14	0.34657094	0.012634094		307133
PPP1R12A	0.4900083	0.012656978	M110|MBSP|Mypt1	116670
ABTB2	0.52522504	0.012657382	Cca3	171440
E2F3	0.6646341	0.012657382	RGD1561600	291105
U2AF1	0.47243112	0.012676724		687575
SLC7A5	0.64414984	0.01268377	E16|TA1	50719
ALKBH4	0.4706152	0.012723504	RGD1308608	288587
B3GALT5	-0.60520667	0.012782929		288161
FEM1A	0.43934175	0.012786375		316131
IRGQ	-0.4388204	0.012798666	RGD1309409	292708
HPS4	-0.36125737	0.012807782		304555
PIK3CB	0.6236102	0.012816889		85243
ASCC2	0.56986994	0.012851208	RGD1561422	498402
WDR45	-0.4638789	0.012886619		302559
PRKCQ	-0.4221288	0.01289057	Pkcq	85420
ZFP575	-0.6800261	0.012934943	Znf575	308430
OSBPL9	-0.42365465	0.012934943		298369
FADS3	-0.3418549	0.012934943		286922
CDKN2AIPNL	-0.51119804	0.012948143	RGD1308696	287278
EME1	0.70008147	0.01298084		287634
GRK5	0.5016199	0.012997693	Gprk5	59075
LCMT2	0.6007042	0.013089576		296098
FAM134A	-0.4706435	0.013092566	RGD1306844	363252
ARF1	0.2535262	0.013098046		64310
SEC13	0.2940295	0.013098046	Sec13l1	297522
KIFC3	0.33618397	0.013098096	KRP4	307644
UROD	-0.39947385	0.013158204	porphyrinogen carboxy-lyase	29421
RCAN1	0.3098915	0.013201669	Dscr1|Mcip1	266766
PSENEN	-0.3733969	0.013263653	RGD1312037	292788
KIF18B	0.54401064	0.013345053	RGD1310360	303575
MEOX2	1.1383067	0.013349704		29279
CMTR2	0.40433457	0.013375053	Ftsjd1|RGD1309394	292016
TMA16	0.66073817	0.013386441	RGD1305222	290686
KCNJ5	1.139306	0.01343561		29713
PDXP	-0.627077	0.013486668	Rbp1	727679

GABARAPL1	-0.3361119	0.013505544	Gec1	689161
FNTB	-0.32460427	0.013505544		64511
CRIPT	-0.5646176	0.013523571		56725
FUT7	0.94434845	0.013527854		296564
SAPCD2	0.5823559	0.013559467		680531
PLK4	0.45452306	0.013645521		310344
WHSC1	0.56271935	0.013645521	RGD1565590	680537
RAPGEF3	0.41137132	0.013665983	Epac	59326
ITGA11	-1.0088171	0.013686501		315744
NUDT22	-0.7193156	0.013696576		293703
E2F6	0.47217578	0.013747766		313978
CCDC175	-0.68771666	0.013760201		500668
TRPT1	-0.62095404	0.013778904	Tpt1h	293704
MAD2L1BP	0.46765026	0.013778904		316237
CYTH1	-0.42518106	0.013819262	Pscd1|Sec7	116691
MAPK3	-0.44389716	0.013960122	ERK1|ERT2|Erk-1|Esrk1|MAF	50689
SRP68	0.2553731	0.013960122		363707
PNKP	0.41672245	0.013967045		308576
APBA2	0.44258118	0.013969976	Mint2	83610
NCOA4	0.2536262	0.01399342		619385
DVL1	0.41346362	0.014030753	dvl-1	83721
CEP104	0.44551	0.014032714	GlyBP	246295
ZFP35	0.46781194	0.014046456	Zfp239	307547
TAS1R3	1.1216946	0.014046456	T1r3	170634
CNNM1	0.64462084	0.0140548		309387
CROT	0.31769052	0.014072188		83842
CLDN9	-1.0229357	0.014098884		287099
VKORC1	-0.584093	0.014123564		309004
ZFAND2B	-0.54397416	0.014211214	RGD1306260	363253
MGAT1	-0.33278877	0.014421033		81519
POPDC3	0.64929414	0.014451542		641520
TPD52L3	-1.1269943	0.014482369	RGD1309391|Trpd52l3	293894
SLC9A3R1	0.2791779	0.01448429		59114
ZFP358	-0.40169713	0.01453809		360754
ELOVL5	0.6144908	0.014565824	rELO1	171400
SLC22A23	0.546605	0.014620495	Nritp	64559
WBSCR17	0.59746975	0.014620495		288611
GRIN2A	1.1237993	0.014670977	GluN2A|NMDAR2A|NR2A	24409
EGLN3	-0.36376014	0.014675131	PHD-3|PHD3|SM-20	54702
RGD1307443	-0.6906255	0.014710051		361244
SLC35A5	-0.42048854	0.014710051	RGD1564361	498081
RSPO3	-0.7410595	0.014804967	RGD1563246	498997
USP1	0.47711465	0.01482081		313387

CELF1	0.6000399	0.014868277	Cugbp1	362160
HSPA12A	-0.4305887	0.014875834		307997
RTFDC1	-0.34047946	0.014875834	RGD1311072	296410
GZF1	0.31087232	0.014882887	RGD1562321|Zfp336	311508
ITGA7	0.5440397	0.014901846		81008
ZFP414	-0.53686273	0.01492048	RGD1308123|Znf414	299647
EIF1B	-0.46571234	0.014938674	RGD1309241	301068
POLR1A	0.60301477	0.014951176	Rpa1|Rpo1-4	83581
ENTPD6	-0.42570895	0.014967268		85260
RGD1565498	0.47882587	0.015042431		500843
KBTBD2	0.3364676	0.015217288		312372
LRRC42	-0.41950768	0.015220032	RGD1308919	298309
MGAT5B	-0.5500894	0.015225536		303693
LRRC55	-0.77066547	0.015227393		311171
PAPD7	0.287108	0.015227393	Pols	306672
COL26A1	-0.7256622	0.015306952		685612
MCCC1	-0.42266458	0.015306952		294972
ST6GAL1	0.30966005	0.015314262	Siat1	25197
FSTL4	0.6717412	0.015316743		303130
TMEM45A	1.1240699	0.015316743		680866
HIBADH	-0.38129097	0.01535139		63938
RGD1304694	0.4424138	0.01535139		362974
ZFP865	-0.4434425	0.015435516	RGD1565545|Znf865	308337
C2CD5	-0.28698078	0.015568304	RGD1304592	362461
KAT8	-0.3544251	0.015594526	Myst1	310194
INO80	0.46175486	0.015594526	Inoc1|RGD1310969	296084
CTTN	0.32078534	0.015613903	Cttnb	60465
UBAP2	0.53915226	0.015616591		313169
PRKRIP1	0.42274854	0.015644388		498171
TMEM42	-0.65324134	0.015668258	RGD1307118	363171
LOC688765	0.57121044	0.015714934		688765
FEZ1	-0.38018176	0.015769731		81730
MRPL14	-0.63503325	0.015814753	L14mt|L32mt|MRP-L14|MRF	301250
PEO1	-0.37689152	0.015904427		309441
FAM127B	-0.40364155	0.015928643	Cxx1a	679038
SLC27A2	-0.83193815	0.015976228		65192
GRB10	-0.3867585	0.016015625	RGD1566234	498416
SLC44A1	0.42136672	0.016015625	Cdw92|Ctl1	85254
GRP	-0.75931096	0.016039979		171101
LRRC6	-0.97423357	0.016069578	Lrtp|Tslrp	299920
BRE	-0.40150228	0.016130334		362704
SLC22A1	-1.0284437	0.016288156	Oct1|Orct1|Roct1	24904
ISPD	-0.5942794	0.016308328	Nip	493574

TSPYL1	-0.30987325	0.016322302	Tspyl	29544
PITPNB	0.24328303	0.016322302		114561
EPHA5	0.42673656	0.016352832	EHK-1|Els1|Els1.	79208
NOP58	0.49382052	0.016380163	Nap65|Nol5	60373
SPON1	0.5561997	0.016466217	Sponf	64456
ATPAF2	-0.41099223	0.016475318		303190
POLE4	-0.47191355	0.016498253		362385
ZC3H14	0.34251946	0.016498253	Npuk68	192359
DIS3L2	-0.39656162	0.01651732	RGD1560168	367307
TMEM39A	0.46733102	0.016577259	RGD1306421	288092
RNF125	-0.96201056	0.016589224		361296
ATP6V0A2	0.57361895	0.016614597	Cc1-3|J6b7|Tj6	116455
HIGD2A	-0.31916255	0.01662287	RGD1309691	290999
TMEM184B	-0.44063416	0.016624494	RGD1306591	362959
AMN	-0.93529457	0.016706992		314459
DOC2B	0.7099712	0.016706992		81820
SATB1	-0.48735073	0.016751138		316164
CIAPIN1	0.42386988	0.01675911		307649
USP15	0.44951677	0.016768282	Ubp109	171329
ZFP93	-0.88612896	0.01677114		296399
TBX22	-1.0988843	0.016847761	Tbx 22	302369
PCDHA9	-0.42499796	0.016847761	rCNRv09	393090
SLC3A2	0.46668822	0.016847761	Mdu1	50567
BTRC	0.47048524	0.016847761	beta-TrCP1	361765
HSPA9	0.6106112	0.016955905	Crp40|GRP-75|Hspa9a|PBP7	291671
LINGO3	0.6689029	0.01696763		690755
GRN	-0.40805992	0.017058002	PGRN	29143
MFSD13A	-0.43792814	0.017107178	RGD1309313|Tmem180	309454
TAL2	0.94439465	0.017111102	Tail2	685229
ADARB1	0.49465048	0.017120646	Adar2|Red1	25367
USP47	0.29100683	0.017180504		308896
OSCP1	-0.37596297	0.017206836	RGD1306596	362595
RGD1565059	-0.7956027	0.017226197		499630
GLYCTK	-0.54001683	0.017226197		684314
LOC100911367	0.5705844	0.017255178		100911367
ADAT1	0.4908129	0.017425861		690810
PTPRE	0.55275923	0.017460024	PTPepsilon	114767
CSTF1	0.33187506	0.017541908		311670
PEX13	0.3088094	0.01754587		305581
KIF19	-0.71489835	0.017548606	Kif19a|RGD1559936	303659
STRAP	0.30872402	0.017723577		297699
AHRR	-1.0712992	0.01773828		498999
STK4	0.39856547	0.017745363		311622

CACNB3	-0.37787104	0.017808218	CACH3B	25297
RHBDL1	-0.7174056	0.017820828	Rhbdl	117025
GPR160	-1.0051844	0.017873589		499588
LIAS	-0.52962303	0.017964575		305348
TMEM107	-0.5602709	0.017983124		691750
RNF19B	-0.3713631	0.018190706	Ibrdc3	313806
MTERF4	0.3561925	0.018190706	Mterfd2	363289
STRN3	0.31938872	0.018196741	Gs2na|Sg2na	114520
VOPP1	-0.31295136	0.01823828	Ecop|RGD1306494	362374
SEC22A	-0.5121607	0.018246332	Sec22l2	117513
PTRH2	0.3744117	0.018246332	RGD1306819	287593
ZFP259	0.2686922	0.018263027	RGD1562173	500989
MYH7	-0.7258306	0.018271018	Bmyo|Myhcb|myHC-beta|m	29557
TSC22D2	0.5230815	0.018339287		499624
WDR55	0.50927746	0.018367928	LRRG00133|RGD1305640	307494
ANKMY2	-0.5344849	0.018450173	Gna14	314046
SCARF1	1.0565767	0.018450173		303313
ZBTB20	-0.70726675	0.018489016	RGD1560387	288105
NT5C1A	-0.8567769	0.018526126		313574
KCNJ9	-0.45441687	0.018526126	Girk3|Kir3.3	116560
RAB11FIP2	-0.3372077	0.018526126	RGD1308538	308003
PBX4	-0.6258097	0.01855699	Edg4	361131
ABHD14A	-0.7208101	0.0185698	Dorz1|RGD1309721	300982
FARS2	-0.53872687	0.018574912	Fars1	306879
GPR61	-0.5982247	0.01859059		310780
BROX	-0.3832211	0.018613735	RGD1307161	305031
ZCCHC8	0.4474043	0.01883635		288661
HRK	-0.4555785	0.018928798	Bid3|Dp5	117271
USP11	-0.30351844	0.018964652	Uhx1	408217
PRSS36	-0.6603448	0.019085756		497040
KLHDC1	-0.65808123	0.019107081		314190
AAK1	0.53069514	0.019107081	RGD1563580	500244
PDGFC	0.74214494	0.019112902	SCDGF	79429
SURF2	0.75827086	0.019112902		619345
STIM1	-0.3825069	0.019153882		361618
ZCCHC24	0.30016264	0.019153882	RGD1306164	361104
PPP1R15B	0.42969123	0.01926163		304799
CBX7	-0.637661	0.019262733		362962
CCM2	0.3330669	0.019262733		305505
ASB8	-0.46658117	0.019325513		315287
SEC23B	0.46436992	0.019342592		362226
FGF22	0.56972426	0.019345097		170579
CAMSAP3	-0.42870077	0.019371033	RGD1307246	689074

ATRIP	0.3553003	0.019394368		301014
CPSF7	0.39920464	0.019409502	RGD1305441	365407
METRNL	0.83298993	0.019446347		316842
VEZT	0.27029166	0.019518334		299738
SFTPA1	1.0221486	0.019572165	PSAP|PSP-A|SP-A|Sftp1|Sftp	24773
TRIM3	-0.3747128	0.019587457	Berp|Rnf22	83616
PGAM5	0.47645056	0.019648103	RGD1312028	288731
RNF34	-0.35055918	0.01968367	Momo	282845
BMP7	0.5152237	0.01968367	BMP-7	85272
IDI1	0.5555658	0.01968367		89784
SPTLC2	0.5762459	0.01968367	Pomt2	366697
SCNN1G	1.0740247	0.01968367	ENaC	24768
LARS	0.5295304	0.0197218		291624
EBF1	-1.0893587	0.019734237	Ebf|Olf1	116543
ZFP503	0.55792856	0.019744331	Nolz-1|Nolz1|Znf503	305687
NTNG2	0.64086926	0.01978038		311836
CCNL2	0.6622199	0.01978038		298686
PKNOX2	-0.5545568	0.019788744		680549
FBXO3	-0.28655007	0.019788744		690634
PSME4	0.48247126	0.01982289		498433
GNB5	-0.48535332	0.019877074		83579
BRAP	0.31765878	0.01990787		687346
RAB36	-0.407907	0.019967712		690407
FAM163B	0.5645095	0.019967712		685169
F8	0.5986479	0.019967712		302470
PWWP2B	0.70017093	0.019967712	Pwwp2	361671
GPR155	-0.38577795	0.020069456		311730
TNIP2	0.6872239	0.020082528		305451
APBB1	-0.36937773	0.02017821	FE65	29722
ALG9	-0.32408738	0.020257102	Dibd1	367083
KDELR3	0.44304436	0.020277565		315131
MESDC1	0.28909972	0.020294696		308795
SEPHS1	0.33469778	0.02044995		291314
AIG1	-0.42043447	0.020462526	RGD1562920	292486
CCDC91	-0.60597724	0.0204778	Ccdc91	103690009
CALY	-0.7370509	0.02062924	Calcyon|Drd1ip	192349
FIP1L1	0.42561775	0.02062924		289582
GJB2	0.87944615	0.02066671	CXN-26|Cx26	394266
SNRNP40	0.26904786	0.020728698	RGD1309198	313056
MPDU1	-0.49394405	0.020758046		303244
ZMPSTE24	0.4096436	0.020809803		313564
LYRM7	-0.7201525	0.020845149		686506
DHX33	0.6332344	0.021010872		287464

MAP2K1	0.46555167	0.021087652	Mek1	170851
TRAK1	-0.44992325	0.021176055	RGD1307844	316085
HCN3	-0.58350736	0.021250414		114245
COX11	0.3311598	0.021282801		690300
DECR2	-0.7600805	0.021355916	Dcrakl|putativeperoxisomal2	64461
NEMP2	0.3647779	0.021356642	RGD1560421|Tmem194b	503257
MPHOSPH10	0.49548197	0.021419972		293828
FAM212A	-0.98108214	0.021421747	RGD1560778	316001
CDC45	0.44240254	0.021473555	Cdc45l	287961
TRIM36	0.64333475	0.02148699		291597
ATF4	0.45487285	0.021494819		79255
NVL	0.39071107	0.021549927		289323
SHTN1	-0.3805176	0.021569615	RGD1311558|Shootin1	292139
MCCC2	-0.4543736	0.021715138		361884
TWIST2	-1.073762	0.021729017	Dermo1	59327
WDR34	-0.35201335	0.021729643		296618
UBE2D1	-0.5558267	0.02176936		361831
ANGPT2	0.9950371	0.02176936	Agpt2|Ang-2	89805
MSTN	-0.9719133	0.021809116	Gdf8	29152
SPIN2A	-0.66989213	0.021809116	RGD1560898|Spin2	317395
CDH7	-0.7649024	0.02182333	Cad7	29162
NOP14	0.37772366	0.021906657	Nol14|RGD1305605	289724
AZIN2	-0.74265516	0.02193875	Adc|Azl2|ODC-p|RGD156477	366473
TUBGCP3	0.33934143	0.021972759		306599
VPS37C	0.3543599	0.022023605	RGD1309258	308178
COG3	0.46740416	0.022023605		361073
SCLY	-0.41821226	0.02206824		363285
MIDN	0.48622456	0.02206824		314623
SLC5A10	1.0390279	0.02206824		303205
MAP7D2	0.6383688	0.022087066	Brelin|Mtap7d2|RGD156485	317508
DDX31	0.40275928	0.0221322		311835
ALDH3A2	0.41460213	0.0221322	Aldh4|FALDH	65183
EHD3	0.45017213	0.02218002	Ehd2	192249
CCDC116	0.72348905	0.022228336	Sdf2l1	287936
SCHIP1	0.44048518	0.02227399		295105
NEUROD2	-0.57589155	0.022393469		54276
ZFYVE1	-0.39420262	0.022393469		299188
ST18	0.9323301	0.022393469	Nzf3|r-MyT3	266680
SFI1	-0.3593983	0.02240066	RGD1560636	305467
IARS	0.5432607	0.02241786	Iarsl	306804
THNSL1	-0.41821316	0.02246256		498805
KRT16	1.0515325	0.022464631	Ka16|Krt14|Krt14l	303530
SLC39A10	0.41648242	0.022551391		363229

SSR1	0.23996204	0.022580769	Ac2-238	361233
TMEM95	0.99867725	0.02259108		691982
MRPS12	-0.37447104	0.022634368		292758
TMEM19	-0.45933667	0.022701034		299800
RBM26	0.35263962	0.022743149	RGD1308297	306137
DPYD	-0.40603387	0.022779727		81656
DPM2	-0.34453657	0.022779727		29640
ICAM5	0.6350212	0.023105703		313785
NR1H4	1.0299447	0.02314844	Fxr	60351
AMPD2	-0.35778847	0.02316695	Ampd	362015
SF3A1	0.30246204	0.023192532		305479
RTF1	-0.37656203	0.023204755	Gtl7	366169
BNIP1	-0.47995958	0.02324584	Bnip1l1	140932
C2	0.65125006	0.023256697		24231
EIF5A	0.3161303	0.02325939		287444
PODXL2	-0.46132386	0.02325958	RGD1305815	297433
GPRC5D	1.0628122	0.023274288	RGD1563887	500349
EBPL	-0.81103384	0.023533585		361054
RNFT2	-0.47578886	0.023533585	RGD1560195|Tmem118	304521
SDR39U1	-0.47096366	0.023644267	RGD1309307	361044
SYNJ2BP	0.44070727	0.02364881	Omp25	64531
HDGFRP3	0.23701152	0.0237519		252941
POFUT2	0.2858751	0.02376734		309686
AGPAT4	0.3172423	0.023829859		170919
ARHGAP26	0.6762538	0.023879444		307459
PTH2	1.0623065	0.0239008	RGD1559447|Tifp39|Tip39	499149
HNMT	-0.7935456	0.023969265		81676
EXOC6B	0.51423776	0.024024148	RGD1560638	500233
ENHO	-0.77281755	0.024061421	RGD1565232	100912292
ZFP598	0.38935694	0.024068875	Ntrap|Znf598	287119
HEATR5B	-0.47741523	0.024102585		362683
RAB26	-0.6244838	0.024215654		171111
ZMAT3	0.60328597	0.024220372	PAG608|Wig1	64394
FBXW17	-0.52784884	0.024243416	RGD1566133	361219
TRIM45	-0.6569792	0.024276678		295323
SMIM3	-0.43222162	0.02429222	Nid67	286910
PCDHA3	-0.4330155	0.024306206		116780
HIPK2	0.72276527	0.024310857		362342
DSG3	1.039333	0.024361389		291752
DUSP8	-0.4727663	0.024405938	Hb5|M3/6	361679
NR1I3	-0.47867706	0.024651837	CAR	65035
MAP3K5	-0.5257784	0.024662388	Ask1|RGD1306565	365057
RNMTL1	0.5180982	0.0246857	RGD1309077|Rnmtl1	360569

NXNL2	0.8030359	0.0246857	Rdcvf2	689232
CLTC	0.4065264	0.02470702		54241
DCTD	0.5415651	0.02474614		290741
GMPPB	0.35616145	0.024762154	RGD1560458	363145
SHISA2	0.67983407	0.024807438	RGD1561256|Tmem46	498528
N4BP2L2	0.4002115	0.024890715		288416
RGS7	0.4333365	0.024890715		54296
PLPPR3	-0.36046594	0.025061086	Lppr3|Prg-2	314614
RGD1309870	-0.9370675	0.025096979		289778
SMARCA2	-0.3563186	0.025176045		361745
ATMIN	0.3480729	0.025186088	Asciz|RGD1305781	315037
CCBL2	-0.4399762	0.025223214	Kat3	541589
CIDEA	1.0524814	0.025334332		291541
CRIP2	-0.36622167	0.025359625	CRP2	338401
HIRA	0.5345021	0.025456967		363849
TWISTNB	0.56608593	0.025475383		362728
ENTPD3	-0.6030954	0.025481807	NTPDase3	316077
PMP22	0.30523092	0.025500532	Gas-3	24660
TRPC7	-0.9399961	0.025614291		282822
KATNBL1	0.4713918	0.025625682		691543
USP24	0.584131	0.025641214		313427
RAVER1	0.44098482	0.025709024	Raver1h	298705
FNDC5	-0.41621748	0.025931872		260327
PMAIP1	0.9662358	0.025931872	Noxa	492821
NR2F1	-0.6552084	0.02600139	COUP-TFI	81808
PDIA5	0.75548553	0.02600139		360722
HPDL	0.6105229	0.026015805	Gloxd1|RGD1310014	313521
INHBB	0.5896933	0.02605992		25196
CAPN1	-0.8966967	0.026103247		29153
EHF	1.021225	0.026108855		295965
ARFIP1	0.44239298	0.026112726		60382
MCL1	0.3782775	0.026118701		60430
SIKE1	0.24370816	0.026174108	RGD1311316|Sike	362007
SRPK2	0.29034224	0.026197786		296753
ZBTB9	0.33083826	0.026280228	3930402F13Rik	294289
ZFP213	-0.49818873	0.026280472	Znf213	287094
NTM	-0.28882337	0.026316976	Hnt|RNU16845	50864
AIFM1	-0.3125007	0.026340885	Aif|Pdcd8	83533
CASP8AP2	0.4594791	0.026352275		313128
LDHA	0.43479967	0.026459916	Ldh1	24533
VPS25	-0.42980328	0.026719892		681059
SLC25A20	-0.3190037	0.026738843		117035
LOC100910945	-0.46788025	0.026746746		100910945

SNCA	-0.6050173	0.026817571		29219
PLEKHA1	0.25035676	0.026821863	RGD1564153	361659
AKT2	0.5054009	0.026897125		25233
KLF4	0.90540993	0.02692236	GKLF	114505
DEPDC5	-0.40845168	0.026927		305464
NGB	-0.6928232	0.026994903		85382
IVD	-0.39578953	0.027016677		24513
PSMG1	-0.47655776	0.027284902	Dscr2	288236
GORASP2	0.2791223	0.027284902	GRASP55|Grs2	113961
ATP10A	0.7995832	0.02731377		365266
TBL3	0.31145382	0.027333464		287120
QDPR	-0.28917953	0.02734116		64192
SPSB4	-0.6106562	0.027451115	RGD1562307	300950
TYR	-0.9649715	0.02745966	C	308800
RNASEH2A	-0.5348562	0.02767752		364974
SDPR	-0.81919664	0.027761383		316384
CACNG8	0.47115186	0.027788473		140729
IFNG	0.96064746	0.027807288	IFNG2	25712
RBL1	0.63687265	0.027824886		680111
RANBP6	0.26083702	0.027894719		309326
GPM6A	-0.5804157	0.028009865	M6a	306439
TIMELESS	0.5192112	0.02803853	Tim	83508
SLITRK3	-0.5794967	0.028080568		310519
CEP120	-0.25485238	0.028080568	Ccdc100|RGD1565619	307302
MIPEP	-0.30178705	0.02809246		81684
CDC42SE1	0.23582925	0.028155314		499672
SLC41A3	-0.53476036	0.028180331		641603
TMEM8A	-0.41689226	0.02822031	M83|Tmem8	303004
ACTN2	-0.4354072	0.028269438		291245
VPS53	-0.28410313	0.028292362	RGD1311391	287535
COMMD1	-0.6125608	0.028329818		289831
CDH22	0.44884676	0.028351601		29182
HIST3H2A	-0.5952332	0.028359205	H2a	64646
EFNA1	-0.98023325	0.028365927	B61	94268
RNF111	0.2780099	0.028372575		300813
WDHD1	0.58513665	0.028372575		305827
LASP1	-0.43409932	0.028413778		29278
CDK2AP1	0.51430607	0.028428685		360804
SDC2	0.40368718	0.02844198	HSPG	25615
DGUOK	-0.7135703	0.028450714		297389
TNFRSF11A	-0.5661064	0.0285034	RANK|RGD1563614	498206
SPATA2L	0.43527207	0.028505994	RGD1560840	498963
TANGO2	-0.4104154	0.028557584	RGD1310348	360738

LYAR	0.6589188	0.028607719		289707
TAF5L	0.6951462	0.028670723		307927
FIBP	-0.52428794	0.028681373		282837
N4BP2L1	-0.5847066	0.028689358		498131
PCBP2	0.33674398	0.028723367		363005
RGD1309104	0.38246995	0.028741185		289084
EIF2AK4	-0.41502848	0.028792525		114859
DPYSL2	-0.5521578	0.02881728	Crmp2|TOAD-64	25416
NAAA	-0.9980757	0.02890205	Asahl	497009
CHMP6	-0.37391767	0.028960612	RGD1565325	287873
ACKR2	-0.9902222	0.02896535	Ccbp2|D6	140473
TMBIM6	0.24474776	0.02903055	Ab1-011|Ac1-149|Cc1-27|Te	24822
SAPCD1	-0.5870442	0.029040692	Ng23	406170
GPAT4	0.29193175	0.029048502	Agpat6|RGD1310520	290843
GPR26	0.79486054	0.02906515		192153
DMC1	-0.954366	0.029074695	Dmc1h	362960
ACAA1	-0.3464555	0.029277159	Acaa|Acaa1a|Pktaa	24157
MKRN1	-0.32218188	0.029280579		296988
GDAP2	0.2811545	0.02932361		362004
GPANK1	-0.55446744	0.029328948	Bat4	415064
BLOC1S5	-0.52388054	0.029396914	Muted	306868
MAPRE1	0.30936545	0.029399548	Eb1	114764
ZIC2	0.5563749	0.029415943		361096
SLC2A13	-0.44457495	0.029434267	Hmit	171147
TIMP3	0.66125244	0.029497175		25358
SLC2A1	0.4447203	0.029523157	GLUTB|GTG1|Glut1|Gtg3|RA	24778
GFRA2	0.7065282	0.029685542	Retl2	25136
RARS	0.41149497	0.029705409		287191
FAF1	0.34779784	0.029716888		140657
TPM2	0.8883627	0.029716888		500450
SPCS3	0.35186204	0.029738758		680782
NUDT14	-0.6949743	0.029768933		299346
TRAPPC2B	-0.56372106	0.02983081		100910318
SESN2	0.63824654	0.029846989	RGD1566319	502988
EXOC3L1	-0.6660272	0.029977003	Exoc3l|RGD1311132	291961
MYPOP	-0.6344545	0.03004929	P42pop|RGD1565160	499090
SYT13	-0.48842904	0.030050488		80977
LOC689986	-0.37586313	0.030110314		689986
IFT122	-0.36788243	0.030110314	Wdr10	312651
SWSAP1	-0.48390603	0.03014336	RGD1308026	363029
LRRC34	-0.8412541	0.030171528	RGD1565052	499589
ZHX2	0.55784607	0.030193167		314988
FBXL14	0.4120996	0.030285114		312675

SLC4A5	-0.92623943	0.030432098	NBC4	297386
SRCIN1	-0.5569027	0.030438466	P140|Snip	56029
IRX6	1.0180786	0.030557713	RGD1564830	307715
TRERF1	-0.6184687	0.03057336		316219
WNT16	-1.0102184	0.030616408		500047
ZFP697	0.8302689	0.030626684	RGD1308711|Znf697	295310
CACNB4	0.641858	0.030679084		58942
THSD7A	-0.6736665	0.030782651	RGD1566201	500032
PCDHGA2	-0.493664	0.030789359		498846
LARS2	-0.39411244	0.030789359		363172
CUL2	0.24325298	0.030789359		361258
CLINT1	0.54087186	0.030789359	Clint|Enth|Epn4	360515
NIT1	-0.2994909	0.030802669		289222
AARS	0.37740648	0.030811546		292023
LHFPL5	-0.72176534	0.030828876	Tmhs	294303
BCL2L11	-0.5232826	0.030841896	Bim|BimL	64547
HDX	0.7631826	0.030876638	RGD1563666	317617
APCS	0.9416904	0.030882161	Sap	29339
SLC4A10	-0.45854294	0.03095376	NCBE	295645
CCDC65	-0.37604862	0.03095376		362994
RHOT1	0.26838666	0.031272113		303351
STAT2	-0.37317953	0.031295735		288774
SCN1A	-0.72736907	0.031329434		81574
STEAP2	0.40834293	0.03136245		312052
RAMP2	-0.6928447	0.031428874		58966
CPT1C	-0.35671914	0.031512763	CPT IC|CPT1-B|CPTI-B	308579
FBXL20	-0.6405506	0.031550296	Fbl2|Fbxl2	64039
NRG1	0.55282503	0.0316391		112400
S100PBP	-0.5235443	0.031642627	RGD1564943	500551
NAA40	0.51848483	0.031666256	Nat11|RGD1565838	361718
IL25	-1.0208933	0.031695936	RGD1561632	501996
DCPS	-0.34323475	0.031695936	Hint-5	266605
CORO2A	-0.488291	0.031715386		313235
RNF5	-0.45976725	0.031731933		407784
HK2	0.47732583	0.03179401		25059
GEMIN8	-0.41399086	0.0318039	Fam51a1	363462
POU1F1	1.0202218	0.0318039	GHF1|GHF1A|PIT1Z|Pit1	25517
DCAF7	-0.3669658	0.031858068	RGD1305140|Wdr68	303602
ATP6V1B2	0.42750973	0.031886395	Atp6b1b2|Atp6b2|Vatb	117596
SMAP2	0.3646476	0.03202195	RGD1308418|Smap1l	298500
ATP11A	0.49652663	0.03202961	Ua20	306600
DDB2	-0.5134682	0.03221289		100362121
RGD1308601	0.568617	0.0323027		307249

TRIP12	0.48558667	0.03232823	Gtl6|TRIP-12	316575
RAD21	-0.26925308	0.032404546		314949
RGS5	0.9978223	0.03242148		54294
POGLUT1	0.39570394	0.032437116	Ktelc1|RGD1306248	288091
PATL1	0.5256846	0.032437116	Pat1b|RGD1305514	361736
SLC1A4	0.54220474	0.032437116	ASCT1	305540
XRCC4	-0.39937168	0.032484468		309995
LRRC41	0.385255	0.032493487	RGD1311221	362566
LOC680039	0.46632928	0.032493487		680039
TLK2	0.31502602	0.032496337		303592
NCSTN	-0.26698932	0.032625727		289231
SPATS2L	-0.40924394	0.032649647	RGD1309930	316426
MPP4	0.7599157	0.032875896	Dlg6	58808
SLC5A8	1.0145775	0.033064038	RGD1564146	500820
BARHL2	0.9757056	0.033074524	Barh|MBH1	65050
ERF	0.47308475	0.033135038		292721
42982	-0.764245	0.033291806	EG3-1RVC|EG3RVC|Pnutl2	287606
B4GALT5	0.33854157	0.033291806		362275
MTERF2	-0.63347954	0.033466984	Mterfd3|RGD1311836	366856
USF1	-0.32495648	0.033466984		83586
PKNOX1	0.6513941	0.03349886		294322
LOXHD1	1.0126847	0.033543635		291427
PANK4	0.3367602	0.03362458	Fang1	171053
DHRS3	0.47700343	0.033664837		313689
PHF1	-0.4050112	0.033805452	Tctex3	294287
CDC14B	0.7017648	0.033805452		361195
ZFYVE16	-0.5173794	0.033865884	RGD1564784	499508
AKR1E2	-0.483803	0.033865884	Akr1cl2|Akr1e1	307091
CHURC1	-0.601889	0.033934236		299154
USP49	-0.6277844	0.034041964	RGD1310513	316211
CRYM	-0.53354144	0.03418983	CDK108	117024
SRSF9	0.3987796	0.034256175	Sfrs9	288701
CDPF1	-0.5317033	0.034327634	RGD1306001	362975
RPH3A	-0.6514723	0.034347903		171039
CCDC53	-0.4767593	0.034459036	RGD1563761	299707
PHF14	-0.41190183	0.034459036	RGD1563764	500030
MINK1	0.39588234	0.034459036	MEKKK 6	303259
NET1	-0.32405576	0.034461897		307098
TLE3	0.3218495	0.03449508	Esp3	84424
PAPD4	0.43107226	0.03457216		361878
MESP1	-0.97030115	0.034614347		308766
GABRQ	0.72086304	0.034620695		65187
RAB14	0.27399725	0.03472653		94197

MFSD14B	0.24501477	0.034771945	Hiatl1|RGD1308377	306687
MCM10	0.53480804	0.034814026		307126
TNFAIP2	-0.80376416	0.034970935		299339
RASSF6	-1.0063156	0.03497475		305251
VRK3	-0.3422794	0.035095934		361565
SEC61A2	0.2270908	0.03526537		361273
PEX5	0.5473612	0.03528934	PTS1-BP|PTS1R	312703
NME7	-0.45875064	0.03532765	Nm23-r7	171566
DDX19B	0.31900993	0.035466228	Zd10a	690693
DNMT1	0.34042725	0.035562135		84350
GPRASP1	-0.24657959	0.035594195	pips	171407
CCNO	-0.83561397	0.03564552	RGD1565217	499528
CACUL1	0.45192146	0.03565909	RGD1308127	365493
PLAT	0.5291216	0.03566246	PATISS|tPA	25692
MRGBP	0.6698152	0.035740416	RGD1308612	311713
PDE12	0.5205767	0.03574115	LRRGT00074|RGD1310975	306231
B3GNT3	-0.95138377	0.03587172		290638
TGFB1	0.6852653	0.035985652	Tgfb	59086
CHML	0.8247513	0.036103465		689102
CDK5RAP3	-0.41523105	0.03611087	C53	80278
TSPAN15	-0.38312688	0.0361306		679462
PPP2R5A	0.3153681	0.036137		312754
PDZD7	-0.5707858	0.036299657	Pdzk7	293996
JPH3	-0.42839488	0.036348023		307916
SLK	0.5161005	0.036348023	Stk2	54308
MMP11	-0.59045744	0.03639457	ST3	25481
ALAS1	0.27712983	0.0364049	ALAS	65155
SLC35D3	0.7441862	0.03642329		308717
NOP2	0.30664676	0.036445048	Nol1	314969
ZFP39	-0.43446797	0.036447357		303173
ZFP397	0.6151469	0.036568984	Znf397	100909408
ITPRIPL2	0.9190477	0.03661702	RGD1564255	499253
ALDH1L2	-0.44637772	0.0366413	RGD1309458	299699
MCEE	-0.5760194	0.036644287		293829
ELP5	0.28309202	0.036664568	DERP6|Rai12	287446
SNN	-0.3975091	0.03668417		29140
TUBG2	-0.46170938	0.03676427		680991
CDK5	-0.45083693	0.03676427		140908
TUBA3B	0.98023945	0.036987737	RGD1565155	500363
PPWD1	0.34223482	0.037053958	RGD1310204	294711
ZC3H8	-0.52011245	0.037083894	Zc3hdc8	311414
SC5D	0.3328679	0.037091367	Sc5dl	114100
SLC35D1	0.5396388	0.037110966		298280

RGD1309079	0.42236546	0.037203502	Ab2-095	315891
SZRD1	0.2651899	0.03728946	C1orf144|RGD1560286	500575
PGGT1B	0.33448288	0.03728946		81746
CROCC	-0.508079	0.037360642		313663
ZGPAT	0.29030257	0.03737169	RGD1310801	296478
ADPGK	0.31411034	0.03744561	RGD1306103	315722
HTR5A	-0.67148125	0.037459828	5HT5A|MR22	25689
PERP	0.590914	0.037459828		292949
RPS6KB1	0.37649137	0.03754645		83840
CREG1	-0.26421645	0.03756935	Creg	289185
NMRK1	-0.60891306	0.037603244	Nrk1	499330
ARRDC3	-0.42791298	0.037853606	LRRGT00048	309945
ARHGEF2	-0.50566804	0.038085505		310635
ARHGEF25	-0.41118413	0.0381102	Geft	314904
COIL	0.32240334	0.038111575		50998
NKAP	-0.59048504	0.038151268	2610020o08rik	298342
ETHE1	-0.36724612	0.038245164		292710
NBR1	-0.32224736	0.038245164	Ca125|RGD1311421	303554
RPF1	0.58397985	0.038253207	Bxdc5	499725
SLC37A1	0.5571413	0.03828822		294321
SRSF2	0.53308135	0.038305525	Sfrs2	494445
ALG10	0.7551815	0.038305525	Alg10b|KCR1	245960
SLC16A8	0.9730974	0.038305525	Mct3	65200
TRIM54	-0.9230427	0.038337693	Rnf30	362708
PKM	0.5263086	0.038356286	PKM12|Pk3|Pkm2	25630
KIT	-0.5006792	0.038370207		64030
FDXR	-0.5313651	0.0385795	AR	79122
SNX7	0.398886	0.038629517		310815
ENPP1	0.72993696	0.038642246	Npps|Pc1	85496
PAWR	0.5601135	0.03864341	Par-4|Par4	64513
SIRT7	0.42087793	0.038659964		303745
CDC42EP3	-0.44628868	0.03866876		313838
LSG1	0.32500356	0.03869334	RGD1309089	288029
ZFP46	-0.440568	0.03882037		298558
TMEM41A	0.5112608	0.03897502		681708
ADCYAP1	0.6414165	0.03897502	Pacap	24166
CCSAP	0.43907428	0.03899341	RGD1563235	307926
ZFP513	0.26082954	0.039262705	Znf513	313913
CPTP	-0.57773244	0.03937554	Gltpd1	313771
FBXO44	-0.54700243	0.03943288	RGD1562463	500587
HSPA4	0.30015755	0.039481673	Hsp110|Hsp70|irp94	266759
ANKRD52	0.68396103	0.039582606	RGD1307124	362811
NOSIP	-0.43927485	0.03969875		292894

RNF141	-0.36530054	0.039720736		308900
TMEM179	-0.5009015	0.039815802	RGD1310269	314472
RAB11B	-0.41509232	0.039815802		79434
CHEK1	0.50829446	0.039869428		140583
RAB3A	-0.39905143	0.040116232	RAB3	25531
ERAL1	-0.3119239	0.040128108		363646
TACC1	0.471592	0.0402329	Tacc1a	306562
FXYD6	-0.32307225	0.040235706	Php	63847
PHF21B	0.6334849	0.040290035	RGD1308739	300117
GORAB	0.44201726	0.0403276	Scyl1bp1	304923
SIL1	-0.56205726	0.040359624		291673
AK2	0.5197275	0.04037918		24184
UBN1	0.28962803	0.04038311		302935
PIAS3	-0.45282632	0.04042212		83614
JPH4	-0.51739067	0.04046165		445271
PGD	-0.33300146	0.040510073	Cc2-27	100360180
ACADS	-0.35135093	0.040542137	Scad	64304
CAP2	0.34800616	0.04064034		116653
ZBTB8B	-0.50454503	0.040657874	RGD1562327|Zbtb8|Zbtb8l	500553
PADI2	-0.50108236	0.040773638	Pdi2	29511
ERLEC1	-0.46808836	0.040773638	RGD1306508	289874
TCTN1	-0.3580323	0.040773638	RGD1566266	304486
ST8SIA2	-0.69829434	0.040880658	Siat8b	117523
VWA5B2	-0.4674993	0.040880658	RGD1564491	303812
HNRNPAB	0.33188716	0.040880658	A1F-C1|Hnrpab	83498
FGF7	0.96359503	0.040880658	Kgf	29348
LHX4	0.79457676	0.040988516		360858
MED4	-0.36088556	0.041012213	Vdrip	306030
YTHDF1	0.23782311	0.041012213		296467
CDCA7	0.40414777	0.041012213		311742
CBLB	-0.6886468	0.041081518		171136
SAP30BP	0.32180488	0.041081518	RGD1565537	360662
METTL6	0.3548717	0.04120367		290564
MYLK	-0.6736588	0.04122695		288057
ITPR1	-0.43509513	0.041246727	I145TR|IP3R1|InsP3R|InsP3R	25262
CABLES1	-0.31079614	0.041444834		307585
SIRT3	-0.3815202	0.041483484		293615
OBFC1	-0.48785168	0.041653346		294025
LGMN	-0.4387056	0.04174131	Prsc1	63865
SOAT1	0.6703569	0.04174131	Acat-1	81782
CDH9	-0.8313626	0.041754942		29163
ICA1	-0.4694552	0.04176689	Ica69	81024
KDM1B	-0.31811586	0.04183083	Aof1	306819

CEP57L1	-0.5037394	0.041924905	cep57R	294519
CAMK1	-0.48706043	0.042015456	Camki|Gaip	171503
SRFBP1	0.40503505	0.042069778	p49/STRAP	291469
TRIM2	-0.30773902	0.0421367		361970
TSPAN31	0.2815228	0.042165656	Sas	362890
TNRC18	-0.57466465	0.042166073	Zfp469	304302
GPR101	0.9520749	0.042299286	RGD1564196	317608
GPX3	-0.553081	0.042353693	GPx-3|GPx-P|GSHPx-3|GSHP	64317
ZFP521	0.37923446	0.042425748	Znf521	307579
PRR5	-0.6435585	0.0425764	Protor1	315189
RASGRP1	0.4747592	0.042591874	Rasgrp	29434
GGACT	-0.52912664	0.04270827	A2ld1	290500
GTF2F1	0.2910501	0.04270827		316123
POLR1E	0.46758574	0.042786576	Praf1|RGD1565773	313245
MYRIP	0.47532845	0.043024935	Slac2c	360034
MGC95210	-0.425667	0.043049075		287798
ANK1	0.63647246	0.043114673		306570
SOCS6	0.3937871	0.043192185		307200
LOC688390	-0.661496	0.04323364		688390
CSPG5	-0.47791874	0.043257702	Caleb|Ngc	50568
TIA1	-0.39518464	0.043443162		312510
GM2A	-0.31562626	0.043772697		282838
FBP2	-0.8136375	0.043803196	FBPase	114508
ZFP367	0.37631714	0.043803196	Znf367	306695
CRK	0.55220175	0.043810494	Crko	54245
LYPLA2	0.41494977	0.043842703		83510
RGD1565033	-0.5849035	0.04386195		498014
EIF2B5	0.26243556	0.04387254		192234
FAM192A	-0.44237024	0.044030946	Nip30|RGD1307433	307652
EPN2	0.2372426	0.044030946		60443
SV2A	-0.44024414	0.044125818	Sv2	117559
OVCA2	-0.4248868	0.04432062	RGD1564623	497954
CD226	-0.8940893	0.044405293		307199
GLRA2	-0.4702876	0.04443878	RNNEOGLY	24397
NUP62	0.32010227	0.044462617	Np62	65274
TSC22D1	-0.30597922	0.04449759	TS22A|TSC-22|Tgfb1i4	498545
MYBL2	0.502166	0.04449759		296344
TOLLIP	0.3704476	0.044588216		361677
FPGS	0.30807278	0.04468685		687266
PPM1J	-0.68527406	0.044715635		295341
ADSS	-0.2692896	0.044726953	Adss2	289276
FOSL2	0.8236007	0.044848252	Fra2	25446
NEK1	-0.42037255	0.044919748		290705

FUT11	0.37389797	0.044919748		286971
LDLR	0.5501626	0.044919748	LDLRA	300438
PA2G4	0.2990845	0.04504876	Ebp1	288778
PCYT1A	0.5348623	0.045138597	CCT-alpha|CTP	140544
PPP1CA	0.24071914	0.045298655	PP1alpha	24668
TBC1D10A	0.41351005	0.045365326	Tbc1d10	360968
RHBDF1	0.40119374	0.045441378		303008
HES1	0.45293653	0.045441378		29577
ADAMTS10	0.37756658	0.045457304		314655
MYH9L1	0.5848633	0.045488283		25745
BTBD6	-0.35536626	0.045565758		690367
DYNC2LI1	-0.5108052	0.045751467	RGD1310286	298767
SLCO4A1	-0.40388513	0.045751467	OATP-E|Slc21a12	171144
SHISA5	-0.38090327	0.045767896		301013
FANCE	-0.50799674	0.045877352	RGD1561045	309643
CNP	-0.27311778	0.046021413	CNPF|CNPI|CNPII|Cnp1	25275
C2CD2L	-0.36675042	0.046023235	Tmem24	300666
MPV17L2	-0.42423078	0.046042807	RGD1308064	290645
ABHD17B	0.38789335	0.04624426	Cgi67l|Fam108b1|RGD13052	309399
SYNGR4	-0.89858645	0.046292707		292916
DDIT4	-0.54277253	0.04639547	Rtp801	140942
SLC1A6	-0.644496	0.04661186	EAAT4	84012
ZFP709	0.4258446	0.046780914	Hit40|Znf14	266773
FGF13	-0.47861964	0.04680864	FGF-13	84488
EEF2K	-0.41597348	0.046873935	SMEF2K	25435
USP31	0.6709861	0.046946503		308959
SYNGR1	-0.5001973	0.04698841		29205
FAM160A2	0.4618909	0.047051255	FHIP	293343
TTC30B	-0.37847698	0.047068115		499814
ERO1A	0.30140406	0.04717244	Ero1l	171562
PMVK	-0.48543307	0.04733122		310645
RALGAPA1	0.45262334	0.047342747	GRIPE|Garnl1|Tulip1|p240	56785
MAN1A2	0.58379173	0.04750291	Man1b	295319
GPC1	0.44138172	0.047604747	HSPG M12|HSPGM12	58920
NTN1	0.6117326	0.047619216	netrin-1	114523
ZBED4	0.6373196	0.04774105		315211
RBM33	0.62802976	0.047770053	RGD1310651	362297
CH25H	0.8603463	0.047844984		309527
RHPN2	-0.6892688	0.047902454		308516
FAT4	-0.68607205	0.04809833	RGD1564291	310341
GABRD	-0.7051563	0.048128575	GABAA-RD	29689
HACD3	0.26696298	0.04816285	Ptplad1|RGD1565496	300783
DEAR	0.41171983	0.04816285		446170

HOMEZ	-0.4243091	0.048186395		260325
DPY19L1	0.24113286	0.048194468	RGD1305822	315496
MED11	-0.46381506	0.04836857	RGD1563202	287456
HID1	-0.34535822	0.04836857	RGD1311422	287822
RASSF5	0.42041242	0.04836857	Maxp1|Nore1	54355
MEST	0.52117264	0.04836857		58827
SPRN	-0.7253101	0.048448265		541462
WDR46	0.33697125	0.04854942	Bing4	309628
GMPPA	-0.34430286	0.048574977		501167
WDR26	0.35423997	0.048592504	RGD1565589	498301
SENP7	-0.26622754	0.04878576		288167
ZFP583	0.3633975	0.04878576	RGD1562044	499068
SLC25A14	-0.30390215	0.048834655	Bmcp1	85263
ARHGEF11	-0.52531695	0.048962355	Gtrap48	78966
SQLE	0.28197297	0.048962355		29230
LMO1	-0.5265091	0.049009074	Dat1|Lmo3	245979
SLC17A6	0.6108406	0.049009074	Dnpi|Vglut2	84487
PACSIN1	0.6023192	0.04935037		29704
HEXDC	-0.33909637	0.049379133		100216475
SCYL3	0.4040984	0.049412906	RGD1308992	360866
UMPS	0.3245573	0.049528614		288051
HSDL1	-0.39937967	0.04956813	RGD1308433	361418
ISOC1	-0.25592902	0.049739197	DR-NR#1|RGD1307632	364879
CDC25A	0.31931496	0.049744014		171102
YARS2	0.38417423	0.049744014	CGI-04|RGD1311696	287924
NR4A3	0.46416077	0.049744014	NOR-2	58853
SHISA8	0.7559386	0.049744014	RGD1563996	315163
RBPMS	0.8294909	0.049915213	RGD1561067	498642
KLHL25	-0.38356707	0.04996569	RGD1310815	293023
ENPP5	-0.21971719	0.04996569	E-NPP 5|Npp5	316249
ZFP777	0.28266618	0.04996569	RGD1566056|Znf777	502764
TNFAIP1	-0.2973561	0.049998462	Edp1	287543
PABPN1	0.28493616	0.049998462		116697

W+B and RX2+B v V+B LIST

Gene Symbol	Gene ID	log2fc8-1	log2FC_db_wp (WP and DB v DW)
MARCH2	362849	1.0184354	-1.0184354
MARCH8	312656	0.5621597	-0.5621597
MARCH9	679272	1.2818643	-1.2818643
MARCH11	499558	-0.93179196	0.93179196
SEP6	691335	1.0019512	-1.0019512
AATK	690853	1.1776892	-1.1776892
ABCB10	361439	0.7567853	-0.7567853
ABCG1	85264	0.9672269	-0.9672269
ABHD14A	300982	0.98221266	-0.98221266
ABHD6	305795	0.49674532	-0.49674532
ABHD8	306338	1.4025161	-1.4025161
ABI1	79249	-0.3251728	0.3251728
ABTB1	297432	1.1204531	-1.1204531
ACAA1	24157	1.1053482	-1.1053482
ACAN	58968	-1.5901684	1.5901684
ACPP	56780	-1.9549279	1.9549279
ACSL1	25288	-0.25970042	0.25970042
ACSL4	113976	-0.5156731	0.5156731
ACTN1	81634	-1.6871865	1.6871865
ACTN2	291245	0.9964308	-0.9964308
ACTR3B	362298	1.1455386	-1.1455386
ACVR1	79558	-0.89764786	0.89764786
ACVR1C	245921	-2.020098	2.020098
ADAMTS1	79252	-1.3923155	1.3923155
ADAMTS15	300474	1.4631126	-1.4631126
ADAMTS2	287899	1.6056658	-1.6056658
ADAMTS5	304135	-2.0638483	2.0638483
ADAMTS7	315879	1.2275528	-1.2275528
ADAP1	171097	1.135771	-1.135771
ADCK3	360887	1.6959999	-1.6959999
ADCY7	84420	-1.5113342	1.5113342
ADCY8	29241	-1.4688996	1.4688996
ADGRB3	301309	0.9077113	-0.9077113
ADGRE5	361383	-1.1002142	1.1002142
ADM	25026	-0.8431437	0.8431437
ADORA1	29290	-1.5732242	1.5732242
ADORA2A	25369	2.0874836	-2.0874836
ADRA1A	29412	-3.6117969	3.6117969
ADRA1B	24173	-1.7472442	1.7472442
ADRA1D	29413	-2.5322816	2.5322816
ADSS	289276	0.4811795	-0.4811795

AEN	361594	-0.62921613	0.62921613
AFAP1L1	291565	-0.63077354	0.63077354
AFTPH	305544	-0.60534877	0.60534877
AGBL5	362710	0.69131386	-0.69131386
AGFG1	363266	-0.32273915	0.32273915
AGFG2	304375	0.84993124	-0.84993124
AGPAT4	170919	0.29892495	-0.29892495
AGTR1A	24180	-2.2749994	2.2749994
AHCYL2	312192	-0.7150247	0.7150247
AHI1	308923	-0.50456995	0.50456995
AIFM1	83533	0.48263925	-0.48263925
AIG1	292486	0.9889844	-0.9889844
AIPL1	59110	-1.7069412	1.7069412
AJAP1	687031	-0.8164796	0.8164796
AK1	24183	1.2302434	-1.2302434
AK4	29223	-1.5553226	1.5553226
AKAP7	361458	0.8108324	-0.8108324
AKT2	25233	-0.7264136	0.7264136
ALDH1B1	298079	1.5108378	-1.5108378
ALG9	367083	0.6862229	-0.6862229
ALKBH4	288587	-0.537583	0.537583
ALOXE3	287424	-1.671209	1.671209
AMN	314459	3.0723894	-3.0723894
AMOTL2	65157	0.9606952	-0.9606952
AMPD2	362015	0.99161434	-0.99161434
AMZ2	360650	0.91489744	-0.91489744
ANAPC2	296558	1.145698	-1.145698
ANAPC7	304490	0.78994304	-0.78994304
ANGPT2	89805	-1.492057	1.492057
ANK1	306570	-0.7242867	0.7242867
ANKEF1	296184	-1.1365056	1.1365056
ANKMY2	314046	0.89643574	-0.89643574
ANKRD12	316775	0.84804845	-0.84804845
ANKRD13D	361699	1.2342212	-1.2342212
ANKRD33B	310200	-3.6498084	3.6498084
ANKRD34A	295283	1.2890335	-1.2890335
ANKRD34C	300889	-2.4182699	2.4182699
ANKRD52	362811	-1.0459763	1.0459763
ANKRD6	500430	1.0373784	-1.0373784
ANKS1B	314721	-0.758562	0.758562
ANO1	309135	-2.3566136	2.3566136
ANO4	299714	-1.6268474	1.6268474
ANO6	315272	-0.7700067	0.7700067

ANXA11	290527	-1.4498796	1.4498796
ANXA7	155423	-0.34464207	0.34464207
AP1S3	367304	-1.3531628	1.3531628
AP3S2	683402	1.3042969	-1.3042969
APBA2	83610	-0.557084	0.557084
APBB1	29722	1.1575422	-1.1575422
APC2	299611	1.1566436	-1.1566436
APCS	29339	-2.2406497	2.2406497
APOLD1	444983	-1.2710346	1.2710346
AQP11	286758	0.8479076	-0.8479076
ARC	54323	-3.9109151	3.9109151
AREG	29183	-3.612455	3.612455
ARFGAP3	503165	0.5375412	-0.5375412
ARFIP1	60382	-1.0706038	1.0706038
ARHGAP10	688429	-0.851316	0.851316
ARHGAP17	63994	-0.656478	0.656478
ARHGAP20	367085	0.9537013	-0.9537013
ARHGAP25	500246	-1.0276208	1.0276208
ARHGEF25	314904	1.3536028	-1.3536028
ARHGEF28	361882	1.0131143	-1.0131143
ARHGEF3	290541	-1.7099833	1.7099833
ARID5A	316327	-1.8440592	1.8440592
ARIH1	300756	-0.68741125	0.68741125
ARL14EP	311279	-0.94357884	0.94357884
ARMC7	287827	0.30938548	-0.30938548
ARMCX6	363496	1.8307469	-1.8307469
ARPP21	363153	-0.39693156	0.39693156
ARRDC4	293019	0.89794666	-0.89794666
ARSJ	311013	-2.640662	2.640662
ASAP1	314961	-1.2466823	1.2466823
ASCC1	294512	1.1025828	-1.1025828
ASCC2	498402	-0.7461903	0.7461903
ASCC3	309887	-0.6759984	0.6759984
ASGR1	24210	-2.110998	2.110998
ASUN	690728	-0.6417649	0.6417649
ATF1	315305	-0.5402889	0.5402889
ATG10	688555	1.2703956	-1.2703956
ATG16L1	363278	-0.6789589	0.6789589
ATMIN	315037	-0.43551126	0.43551126
ATP11A	306600	-1.0544498	1.0544498
ATP13A3	678704	-1.1816852	1.1816852
ATP1B1	25650	-0.36451632	0.36451632
ATP2B1	29598	-0.34199724	0.34199724

ATP5SL	361520	0.81120443	-0.81120443
ATP6V0A2	116455	-1.0298127	1.0298127
ATPAF2	303190	1.1358901	-1.1358901
ATRAID	298841	1.178951	-1.178951
AUH	361215	1.0459806	-1.0459806
B3GNT2	305571	0.6440816	-0.6440816
B3GNT7	316583	1.7183048	-1.7183048
B4GALT7	364675	1.2259122	-1.2259122
BAALC	140720	-0.8724806	0.8724806
BACH1	304127	-1.7171786	1.7171786
BAD	64639	1.2983373	-1.2983373
BAG3	293524	-2.8785598	2.8785598
BAI1	362931	-0.7593267	0.7593267
BAIAP2	117542	-1.7085128	1.7085128
BANP	292064	0.29416624	-0.29416624
BAZ1A	314126	-1.0645523	1.0645523
BBC3	317673	1.5297897	-1.5297897
BBS2	113948	0.7999286	-0.7999286
BBX	303970	-2.2494485	2.2494485
BCAS3	363662	1.1168277	-1.1168277
BCKDHA	25244	0.8446007	-0.8446007
BCL11A	305589	0.9717859	-0.9717859
BCOR	317346	-0.53614026	0.53614026
BCORL1	302810	0.8902297	-0.8902297
BDNF	24225	-1.5365307	1.5365307
BEND5	362564	1.4132191	-1.4132191
BEND7	361275	-1.3499432	1.3499432
BHLHE22	365748	2.9591675	-2.9591675
BHLHE23	499952	-1.4622635	1.4622635
BHLHE40	79431	-2.205643	2.205643
BID	64625	0.8444431	-0.8444431
BLES03	266609	1.273569	-1.273569
BLOC1S5	306868	0.92540556	-0.92540556
BLOC1S6	317630	0.49731073	-0.49731073
BMPER	300455	-1.1944522	1.1944522
BNIP3	84480	1.0849622	-1.0849622
BNIP3L	140923	1.0528262	-1.0528262
BOK	29884	1.6824747	-1.6824747
BPHL	361239	1.2076544	-1.2076544
BRE	362704	1.037709	-1.037709
BRINP3	286901	1.3785894	-1.3785894
BROX	305031	0.6399443	-0.6399443
BSDC1	297890	0.70258904	-0.70258904

BTBD6	690367	0.77760196	-0.77760196
BTG4	315650	1.8256004	-1.8256004
BTRC	361765	-0.5605399	0.5605399
BVES	365603	-1.4005638	1.4005638
C1QL2	288979	-3.3434925	3.3434925
C1QL3	680404	-1.6164814	1.6164814
C1S	192262	-1.7718594	1.7718594
C2CD2L	300666	0.6377958	-0.6377958
CABLES1	307585	0.7776117	-0.7776117
CACNB1	50688	-0.37143254	0.37143254
CACNB3	25297	1.0428525	-1.0428525
CACNB4	58942	-1.2364125	1.2364125
CACNG3	140724	-1.3195261	1.3195261
CALCA	24241	-2.2412775	2.2412775
CALCOCO1	246047	0.6982477	-0.6982477
CALCRL	25029	-1.2262877	1.2262877
CALHM2	294019	0.9394895	-0.9394895
CALY	192349	1.3429091	-1.3429091
CAMK1G	171358	-0.5225028	0.5225028
CAMKK1	60341	-0.6206306	0.6206306
CAPN2	29154	-0.51820725	0.51820725
CAPN5	171495	1.1776319	-1.1776319
CARF	301446	0.7784706	-0.7784706
CARS	293638	-0.6864734	0.6864734
CAT	24248	0.48430485	-0.48430485
CAV1	25404	-1.3746773	1.3746773
CBFA2T3	361431	-1.1455648	1.1455648
CBFB	361391	-0.62609076	0.62609076
CBLN1	498922	1.3264753	-1.3264753
CBLN2	291388	-0.9794108	0.9794108
CBX8	303731	0.7328807	-0.7328807
CCBL2	541589	1.2280177	-1.2280177
CCDC129	500139	-1.8191351	1.8191351
CCDC148	311051	1.48715	-1.48715
CCDC175	500668	1.6173832	-1.6173832
CCDC184	500925	1.306872	-1.306872
CCDC28A	361454	2.0075464	-2.0075464
CCDC28B	682445	1.71312	-1.71312
CCDC32	296081	0.83603305	-0.83603305
CCDC40	287867	1.0490103	-1.0490103
CCDC53	299707	1.4643389	-1.4643389
CCDC86	293738	-1.2498329	1.2498329
CCDC92	100036765	-0.5050791	0.5050791

CCDC93	304743	-0.6232316	0.6232316
CCKAR	24889	2.4626114	-2.4626114
CCKBR	25706	-2.2215497	2.2215497
CCND1	58919	-1.5370443	1.5370443
CCNG2	29157	1.8686941	-1.8686941
CCNL2	298686	-0.5288063	0.5288063
CCPG1	363098	1.1234567	-1.1234567
CD200	24560	1.1759683	-1.1759683
CD226	307199	1.8789576	-1.8789576
CD55	64036	0.7351284	-0.7351284
CD59	25407	1.0753154	-1.0753154
CDC14A	310806	-1.7185223	1.7185223
CDC25B	171103	0.9233059	-0.9233059
CDC37L1	293886	-0.5121295	0.5121295
CDC42EP3	313838	0.95548755	-0.95548755
CDC42EP4	303653	1.3727965	-1.3727965
CDCA4	500727	-0.63640547	0.63640547
CDCA7L	619566	-0.6370459	0.6370459
CDH15	361432	1.6700618	-1.6700618
CDH7	29162	1.4516338	-1.4516338
CDK19	309804	0.8173471	-0.8173471
CDK5	140908	1.1217455	-1.1217455
CDK5RAP1	252827	1.0830865	-1.0830865
CDK5RAP3	80278	1.1755166	-1.1755166
CDK8	498140	-0.36754665	0.36754665
CDKN2AIPNL	287278	0.944174	-0.944174
CDO1	81718	1.284292	-1.284292
CDYL	361237	-1.3846971	1.3846971
CDYL2	292044	-1.3537384	1.3537384
CELF6	300758	0.9918951	-0.9918951
CEP57L1	294519	0.5916561	-0.5916561
CFB	294257	-1.421618	1.421618
CFLAR	117279	-1.0501424	1.0501424
CH25H	309527	-1.1801852	1.1801852
CHD1	308215	-1.0756745	1.0756745
CHD6	311607	0.6874034	-0.6874034
CHML	689102	-2.2738986	2.2738986
CHMP1A	365024	0.81924915	-0.81924915
CHMP6	287873	1.1243142	-1.1243142
CHN2	84031	1.0361619	-1.0361619
CHODL	288289	2.231705	-2.231705
CHRAC1	315058	0.6425566	-0.6425566
CHRM2	81645	-1.6941352	1.6941352

CHRNA5	25102	1.1824613	-1.1824613
CHST10	140568	-0.43606564	0.43606564
CHST12	304322	1.2780493	-1.2780493
CHST7	302302	-0.5012247	0.5012247
CHSY1	292999	-0.34165925	0.34165925
CHURC1	299154	1.9833574	-1.9833574
CIR1	362149	1.5603967	-1.5603967
CISD3	287661	1.9258878	-1.9258878
CISH	83681	1.444542	-1.444542
CITED2	114490	-1.593563	1.593563
CLASP2	114514	0.70147526	-0.70147526
CLDN9	287099	1.4729203	-1.4729203
CLDND1	288182	-0.4175044	0.4175044
CLIP2	29264	1.1156156	-1.1156156
CLMP	286939	1.8699203	-1.8699203
CLN5	306128	-0.8822822	0.8822822
CLN8	306619	0.57413274	-0.57413274
CMAS	312826	1.2177485	-1.2177485
CMPK2	314004	0.8633549	-0.8633549
CMTR2	292016	-1.0163047	1.0163047
CNGA4	85258	-3.4251666	3.4251666
CNNM1	309387	-0.75169325	0.75169325
CNNM2	294014	0.48656547	-0.48656547
CNP	25275	0.96667814	-0.96667814
CNR1	25248	0.5381837	-0.5381837
CNTFR	313173	-0.86567485	0.86567485
CNTN2	25356	1.4346097	-1.4346097
CNTN3	54279	1.2686888	-1.2686888
COASY	287711	0.91764677	-0.91764677
COBL	305497	0.6472813	-0.6472813
COG3	361073	-0.77866966	0.77866966
COL26A1	685612	1.0989481	-1.0989481
COL4A1	290905	-0.5921858	0.5921858
COL5A1	85490	-1.4546968	1.4546968
COMMD1	289831	1.6570169	-1.6570169
COMTD1	305685	1.2443414	-1.2443414
COX11	690300	-0.71707284	0.71707284
COX19	304330	1.2216917	-1.2216917
CPNE4	367160	1.0142878	-1.0142878
CPNE8	362988	1.2241803	-1.2241803
CRAT	311849	0.6730089	-0.6730089
CREBL2	362453	0.47197583	-0.47197583
CREM	25620	-1.4372749	1.4372749

CRHBP	29625	-3.949865	3.949865
CRIP2	338401	1.1930181	-1.1930181
CRIPT	56725	1.1421247	-1.1421247
CRLF3	54395	-0.7444929	0.7444929
CROT	83842	-0.85425067	0.85425067
CRY1	299691	-0.6343994	0.6343994
CRYM	117024	1.5057176	-1.5057176
CSPG5	50568	0.853645	-0.853645
CSRNP1	363165	-1.3948185	1.3948185
CSRP2	29317	1.7276167	-1.7276167
CTDP1	291414	-0.30255198	0.30255198
CTPS2	619580	0.95587426	-0.95587426
CTSF	361704	1.2331759	-1.2331759
CUBN	80848	-2.3821952	2.3821952
CUL9	316228	1.498927	-1.498927
CUX2	288665	0.9874247	-0.9874247
CX3CL1	89808	-0.6751452	0.6751452
CXCR4	60628	0.8277072	-0.8277072
CXXC5	291670	-0.8889998	0.8889998
CYB561	303601	1.338949	-1.338949
CYB561D2	363137	1.6831125	-1.6831125
CYB5R1	304805	1.3150365	-1.3150365
CYGB	170520	1.8016915	-1.8016915
CYHR1	100362155.chr	1.5363264	-1.5363264
CYLD	312937	0.6384103	-0.6384103
CYP11B2	24294	-1.7526295	1.7526295
CYP11B3	353498	-2.2118537	2.2118537
CYP26B1	312495	-3.2631261	3.2631261
CYP27B1	114700	-2.6242201	2.6242201
CYR61	83476	2.1667793	-2.1667793
CYS1	690489	1.237013	-1.237013
CYTH1	116691	0.5620195	-0.5620195
D2HGDH	301624	1.048223	-1.048223
DAAM1	314212	1.1973464	-1.1973464
DARS2	304919	0.78181463	-0.78181463
DBNDD1	361437	2.8623035	-2.8623035
DBP	24309	1.0060977	-1.0060977
DCAF10	313242	-0.7000545	0.7000545
DCAF4	362762	1.1153011	-1.1153011
DCAKD	360639	0.59539396	-0.59539396
DCLK1	83825	-0.5291546	0.5291546
DCLK3	316023	3.1585078	-3.1585078
DCPS	266605	0.740661	-0.740661

DDAH2	294239	1.7449005	-1.7449005
DDHD1	305816	-0.571644	0.571644
DDIT4	140942	0.8063096	-0.8063096
DDX19B	690693	-0.27219203	0.27219203
DDX20	84473	-0.47132653	0.47132653
DDX21	317399	-1.0781173	1.0781173
DDX3X	317335	-0.42627296	0.42627296
DEAF1	83632	0.9790304	-0.9790304
DEF6	309642	1.9787971	-1.9787971
DEPDC5	305464	0.60728824	-0.60728824
DES	64362	-1.3894205	1.3894205
DESI1	315160	-0.3931853	0.3931853
DGCR8	287954	-0.29063565	0.29063565
DGUOK	297389	0.75320053	-0.75320053
DHRS9	170635	-4.409934	4.409934
DHX40	287595	0.8696716	-0.8696716
DHX57	366532	0.9110711	-0.9110711
DIAPH1	307483	-0.6938537	0.6938537
DIEXF	305076	-0.45094988	0.45094988
DIP2C	307067	-1.248641	1.248641
DIRAS1	366826	1.1724926	-1.1724926
DIRC2	303902	0.7612408	-0.7612408
DIS3	306103	-0.55803543	0.55803543
DISC1	307940	-1.470493	1.470493
DISP2	311324	0.9088202	-0.9088202
DKK2	295445	-4.432964	4.432964
DLC1	58834	-2.8727405	2.8727405
DLEU7	290308	1.9654785	-1.9654785
DLG1	25252	-1.559255	1.559255
DLGAP2	116681	-3.2651875	3.2651875
DLK1	114587	-1.0597112	1.0597112
DMP1	25312	-3.0781653	3.0781653
DNAJB4	295549	0.95473754	-0.95473754
DNAJB5	313811	-0.76502573	0.76502573
DNAJC25	362526	-0.40392032	0.40392032
DNASE2B	59296	-3.7200184	3.7200184
DOC2A	65031	1.2574142	-1.2574142
DOCK8	499337	-1.8718106	1.8718106
DOK4	361364	1.2097769	-1.2097769
DOK5	502694	-1.1789211	1.1789211
DOT1L	362831	-1.4931031	1.4931031
DPF1	50545	1.4777873	-1.4777873
DPF3	299186	-1.2533493	1.2533493

DPM2	29640	1.2595621	-1.2595621
DPY19L1	315496	-0.47533405	0.47533405
DPYD	81656	0.5710019	-0.5710019
DPYSL2	25416	1.0006807	-1.0006807
DRD5	25195	-5.998091	5.998091
DSE	365583	-0.54123944	0.54123944
DSG3	291752	-3.3121614	3.3121614
DTX3	500847	0.92782456	-0.92782456
DUOX1	266807	-1.1840279	1.1840279
DUSP14	360580	-1.9785913	1.9785913
DUSP23	360881	1.6762038	-1.6762038
DUSP26	306527	1.5668025	-1.5668025
DUSP4	60587	-2.832405	2.832405
DUSP5	171109	-2.4208353	2.4208353
DUSP6	116663	-1.7100483	1.7100483
DYNC1I1	29564	0.8546949	-0.8546949
EBF1	116543	1.8131684	-1.8131684
EBF3	361668	-1.4570917	1.4570917
EBPL	361054	1.8555979	-1.8555979
ECEL1	60417	-2.5625746	2.5625746
ECH1	64526	0.8102297	-0.8102297
EDNRA	24326	-1.5468034	1.5468034
EEA1	314764	-1.3007274	1.3007274
EEF2K	25435	0.9492289	-0.9492289
EFNA4	310643	1.9769019	-1.9769019
EFNB3	360546	1.1232499	-1.1232499
EFR3A	362923	-1.2757814	1.2757814
EGR2	114090	-1.3127321	1.3127321
EGR3	25148	-2.6038425	2.6038425
EHBP1	305556	0.5023931	-0.5023931
EHBP1L1	309169	2.03378	-2.03378
EHD1	293692	1.2024988	-1.2024988
EHD4	192204	-1.3532994	1.3532994
EHF	295965	-2.370194	2.370194
EIF1B	301068	1.0278952	-1.0278952
EIF2B5	192234	0.2963045	-0.2963045
EIF5A	287444	0.4837144	-0.4837144
ELAVL4	432358	1.4632306	-1.4632306
ELF3	304815	2.784469	-2.784469
ELMOD1	315670	1.4567773	-1.4567773
ELOVL7	361895	-2.4834602	2.4834602
ELP2	307545	0.6777394	-0.6777394
EMC9	290224	2.015691	-2.015691

EMILIN3	362262	-1.2687697	1.2687697
EML1	362783	0.8281443	-0.8281443
EML4	313861	-0.95312595	0.95312595
EML5	444982	-1.1021036	1.1021036
EMP1	25314	-4.5941815	4.5941815
EMP3	81505	-1.6464361	1.6464361
ENTPD6	85260	0.9032556	-0.9032556
EPB41L4B	500464	-0.9424875	0.9424875
EPDR1	291180	-0.7201802	0.7201802
EPHA3	29210	1.0821818	-1.0821818
EPHA4	316539	0.9911312	-0.9911312
EPHB6	312275	1.5996736	-1.5996736
ERAL1	363646	0.8207574	-0.8207574
ERCC1	292673	-0.49968156	0.49968156
ERRFI1	313729	-1.0401924	1.0401924
ESR1	24890	-1.1356808	1.1356808
ETHE1	292710	1.3022593	-1.3022593
ETV4	360635	-0.70073	0.70073
ETV5	303828	-2.085397	2.085397
EVA1A	500221	-0.9362583	0.9362583
EXO1	305000	-0.83079153	0.83079153
EXOC3L1	291961	1.5982518	-1.5982518
EXOC5	60627	-0.7554035	0.7554035
EXTL2	310803	0.46723768	-0.46723768
EZR	54319	-1.5817354	1.5817354
F2R	25439	-0.67870164	0.67870164
FADS3	286922	0.8076848	-0.8076848
FAIM2	246274	1.2188671	-1.2188671
FAM102B	365903	-0.8933741	0.8933741
FAM107B	498796	-0.6215519	0.6215519
FAM110C	500638	-4.870246	4.870246
FAM110D	500563	2.551216	-2.551216
FAM118A	300120	1.0343237	-1.0343237
FAM127B	679038	0.98202944	-0.98202944
FAM129B	362115	-0.3663069	0.3663069
FAM129C	498604	1.7675214	-1.7675214
FAM134A	363252	0.820052	-0.820052
FAM136A	297415	1.1968178	-1.1968178
FAM150B	679566	-4.36002	4.36002
FAM163B	685169	-0.50279844	0.50279844
FAM184B	289671	2.1667516	-2.1667516
FAM192A	307652	0.84725934	-0.84725934
FAM196A	100233213	-1.6162378	1.6162378

FAM212B	310764	-2.0356653	2.0356653
FAM214A	300836	1.1667423	-1.1667423
FAM220A	498145	0.8113638	-0.8113638
FAM222B	497960	-0.9767736	0.9767736
FAM26F	294430	-2.4521663	2.4521663
FAM43A	288031	-1.4174006	1.4174006
FAM65B	306934	-0.95911074	0.95911074
FAM84A	313969	-1.5026602	1.5026602
FAR1	293173	-1.3329574	1.3329574
FARS2	306879	1.2801592	-1.2801592
FBN2	689008	-0.93448967	0.93448967
FBXL14	312675	-0.8897135	0.8897135
FBXL20	64039	0.5463844	-0.5463844
FBXL6	362941	1.4751589	-1.4751589
FBXO16	305970	1.7636634	-1.7636634
FBXO31	498959	1.4363247	-1.4363247
FBXO41	312504	1.2192515	-1.2192515
FBXO44	500587	1.0990988	-1.0990988
FBXW17	361219	1.3822906	-1.3822906
FCHO1	290639	1.3593366	-1.3593366
FDFT1	29580	1.176812	-1.176812
FDXR	79122	1.5488063	-1.5488063
FEZ1	81730	1.0974953	-1.0974953
FFAR4	294075	-2.5157187	2.5157187
FGF13	84488	1.0222241	-1.0222241
FGF2	54250	-4.242713	4.242713
FGF3	170633	-2.5571735	2.5571735
FGF8	29349	-2.0306342	2.0306342
FGF9	25444	-1.3539819	1.3539819
FGFR1	79114	-2.3136618	2.3136618
FHL2	63839	-0.5822633	0.5822633
FIBP	282837	1.2658006	-1.2658006
FIG4	309855	0.70847756	-0.70847756
FILIP1	246776	2.7372005	-2.7372005
FJX1	366140	-0.46838948	0.46838948
FKBP5	361810	-0.6876135	0.6876135
FLOT1	64665	1.4665278	-1.4665278
FLOT2	83764	1.4728571	-1.4728571
FLRT1	499308	-1.623491	1.623491
FLT1	54251	-2.632133	2.632133
FMNL1	287746	-0.7881743	0.7881743
FN3KRP	303755	0.49876475	-0.49876475
FNDC3A	306022	-1.3759376	1.3759376

FNDC3B	294925	-1.8846078	1.8846078
FOXP2	500037	1.0561723	-1.0561723
FRA10AC1	365458	1.5293473	-1.5293473
FRAS1	289486	-1.3616799	1.3616799
FRMD3	298141	0.5577625	-0.5577625
FRMD6	257646	-1.4485581	1.4485581
FRS3	316213	1.4993623	-1.4993623
FST	24373	3.1059115	-3.1059115
FSTL5	365823	0.41745064	-0.41745064
FTSJ3	303608	-0.36455974	0.36455974
FUCA2	292485	1.1626443	-1.1626443
FUNDC1	363442	0.85060465	-0.85060465
FURIN	54281	-0.75249976	0.75249976
FUT7	296564	-1.479171	1.479171
FXYD6	63847	1.0193477	-1.0193477
FYTTD1	360726	-0.31878003	0.31878003
FZD1	58868	0.79249483	-0.79249483
FZD4	64558	-1.4714973	1.4714973
FZR1	314642	1.0390644	-1.0390644
GABARAPL1	689161	0.7775922	-0.7775922
GABRD	29689	1.7964844	-1.7964844
GABRG2	29709	1.161652	-1.161652
GABRQ	65187	-0.915536	0.915536
GADD45B	299626	-1.4834048	1.4834048
GAL	29141	-2.946706	2.946706
GALE	114860	1.5647572	-1.5647572
GALK2	296117	1.3194399	-1.3194399
GALNT3	366061	0.69954914	-0.69954914
GALNT7	29750	-1.491607	1.491607
GALNT9	304571	-1.1128696	1.1128696
GALR1	50577	-6.854205	6.854205
GAS7	85246	0.69908094	-0.69908094
GBX1	246149	-1.8644722	1.8644722
GCGR	24953	-1.8537114	1.8537114
GCK	24385	4.057229	-4.057229
GCLC	25283	-1.5143988	1.5143988
GCNT1	64043	-3.3693295	3.3693295
GDNF	25453	-1.8685989	1.8685989
GEM	297902	-1.464599	1.464599
GEMIN8	363462	1.0965458	-1.0965458
GFOD1	306842	-1.8363347	1.8363347
GFPT2	360518	1.2477304	-1.2477304
GFRA1	25454	-2.5710554	2.5710554

GINS2	292058	-0.7468228	0.7468228
GJB2	394266	-1.0402259	1.0402259
GLDN	315675	-0.80972207	0.80972207
GLI2	304729	-1.8104296	1.8104296
GLRA2	24397	0.9108136	-0.9108136
GLRB	25456	0.6576773	-0.6576773
GLRX	64045	0.938423	-0.938423
GMPPA	501167	1.1785016	-1.1785016
GOLGB1	192243	0.6012115	-0.6012115
GOLPH3L	310669	0.26587063	-0.26587063
GPANK1	415064	1.29448	-1.29448
GPAT3	305166	-1.7013336	1.7013336
GPATCH2	289362	-1.0378615	1.0378615
GPC2	171517	1.5365086	-1.5365086
GPM6A	306439	0.86119384	-0.86119384
GPR101	317608	-2.4965632	2.4965632
GPR12	80840	2.111301	-2.111301
GPR139	293545	1.1281028	-1.1281028
GPR156	260430	0.9838025	-0.9838025
GPR158	291352	-3.9495723	3.9495723
GPR21	311911	0.32613963	-0.32613963
GPR26	192153	-1.0960113	1.0960113
GPR3	266769	-1.151957	1.151957
GPR37	117549	1.8543845	-1.8543845
GPR4	308408	-0.7456858	0.7456858
GPR45	301372	0.54334664	-0.54334664
GPR50	117097	-2.2457151	2.2457151
GPR52	684623	1.0648395	-1.0648395
GPR61	310780	1.0142778	-1.0142778
GPR63	297952	-2.409319	2.409319
GPR68	314386	-2.2744858	2.2744858
GPR83	140595	-2.6879358	2.6879358
GPR85	64020	0.8875574	-0.8875574
GPR88	64443	-0.5728938	0.5728938
GPRASP1	171407	0.37424657	-0.37424657
GPRC5A	312790	-5.1008306	5.1008306
GPRC5C	287805	1.4053327	-1.4053327
GPX3	64317	1.0541209	-1.0541209
GRASP	192254	-2.925807	2.925807
GREM1	50566	-1.078983	1.078983
GRHL2	299979	-1.4455799	1.4455799
GRIN2A	24409	-3.1377962	3.1377962
GRIP1	84016	-0.62972784	0.62972784

GRK5	59075	-0.7295949	0.7295949
GRM1	24414	1.7649225	-1.7649225
GRM6	24419	1.5778718	-1.5778718
GRN	29143	0.6929331	-0.6929331
GRP	171101	1.7887181	-1.7887181
GRWD1	308592	-0.5673416	0.5673416
GSPT2	501582	-0.37514183	0.37514183
GTF2IRD1	246770	0.80117756	-0.80117756
GTF3C5	362095	0.5371806	-0.5371806
GTPBP4	114300	-1.0845741	1.0845741
GUCY1A3	497757	1.2316636	-1.2316636
GUCY1B3	25202	1.2535505	-1.2535505
GYPC	364837	-3.377353	3.377353
GZF1	311508	-0.41640782	0.41640782
H2AFY2	361844	1.0627307	-1.0627307
HACE1	361866	-1.2985991	1.2985991
HAS1	282821	-2.8278217	2.8278217
HAUS8	290626	-2.3493516	2.3493516
HBEGF	25433	-0.8350807	0.8350807
HCN1	84390	-0.9801577	0.9801577
HCN3	114245	1.0103825	-1.0103825
HDAC11	297453	1.309217	-1.309217
HDX	317617	-1.6189289	1.6189289
HEATR5B	362683	0.72567636	-0.72567636
HECA	308624	-0.65091056	0.65091056
HECTD2	309514	-2.1175406	2.1175406
HEPHL1	500946	-1.8817065	1.8817065
HERC3	362377	0.5495569	-0.5495569
HERPUD2	300463	0.3756691	-0.3756691
HES6	316626	1.6718974	-1.6718974
HIC1	303310	-1.6527004	1.6527004
HIGD2A	290999	1.5173984	-1.5173984
HIST3H2A	64646	1.7466613	-1.7466613
HMG20A	315689	0.5274166	-0.5274166
HMGA1	117062	-2.1395297	2.1395297
HMGA2	84017	-2.8688316	2.8688316
HMGCLL1	367112	1.3452446	-1.3452446
HOMER1	29546	-1.7807477	1.7807477
HOMER2	29547	-0.7872916	0.7872916
HP1BP3	313647	0.68039024	-0.68039024
HPCAL1	50871	1.0417874	-1.0417874
HPN	29135	-1.9175618	1.9175618
HRH1	24448	-1.7427148	1.7427148

HRH3	85268	2.7061095	-2.7061095
HRK	117271	0.6865796	-0.6865796
HS3ST5	294449	-0.7514898	0.7514898
HSD11B1	25116	-0.91425896	0.91425896
HSDL1	361418	0.47044277	-0.47044277
HSF2BP	499413	-3.7208776	3.7208776
HSH2D	100360518	-2.35337	2.35337
HSPA12A	307997	0.68739927	-0.68739927
HSPA1B	294254	1.3913642	-1.3913642
HSPA4L	294993	-1.1560411	1.1560411
HSPB11	685284	1.5743185	-1.5743185
HSPH1	288444	-0.590423	0.590423
HTR1B	25075	-3.1142626	3.1142626
HTR1F	60448	-3.0416794	3.0416794
HTR7	65032	-0.6663122	0.6663122
ICA1	81024	0.96159065	-0.96159065
ICK	84411	0.7886756	-0.7886756
IDH1	24479	0.74111575	-0.74111575
IER5	498256	-1.1308113	1.1308113
IER5L	499772	-0.9994531	0.9994531
IFFO2	641315	-1.5317856	1.5317856
IFNG	25712	-2.047485	2.047485
IFNGR2	360697	0.80174	-0.80174
IFRD1	29596	-0.88495636	0.88495636
IFT122	312651	0.8522669	-0.8522669
IFT52	362265	0.8561634	-0.8561634
IGF1	24482	-1.4307919	1.4307919
IGF2BP2	303824	-1.0935881	1.0935881
IGSF3	295325	1.2240644	-1.2240644
IGSF8	304979	1.394032	-1.394032
IGSF9	304982	2.1925702	-2.1925702
IL1RAP	25466	-1.0855343	1.0855343
IL1RL2	171106	-2.4500093	2.4500093
IL25	501996	1.6658764	-1.6658764
IL34	498951	2.079161	-2.079161
IL6R	24499	-2.765288	2.765288
IMPDH1	362329	1.1701523	-1.1701523
ING4	297597	1.3283275	-1.3283275
INHBA	29200	-2.3986232	2.3986232
INIP	298032	-0.40450808	0.40450808
INO80C	291737	0.64258486	-0.64258486
INPP5D	54259	-1.4566199	1.4566199
INSC	293166	-3.3949947	3.3949947

IPPK	306808	-1.4250371	1.4250371
IQSEC3	404781	-0.8191139	0.8191139
IRF6	364081	-1.33301	1.33301
IRGQ	292708	0.5293753	-0.5293753
IRS2	29376	-1.7454215	1.7454215
IRX6	307715	-1.503687	1.503687
ISG20L2	361977	-0.82030123	0.82030123
ISM1	311760	-3.1640146	3.1640146
ISOC1	364879	0.45082656	-0.45082656
ITGA11	315744	1.8450263	-1.8450263
ITGA6	114517	-2.3379338	2.3379338
ITM2C	301575	1.0857115	-1.0857115
ITPR3	25679	-1.2132822	1.2132822
ITSN1	29491	-0.89033645	0.89033645
IVD	24513	0.46442395	-0.46442395
IWS1	291705	-0.4862273	0.4862273
JADE3	299305	-1.4989351	1.4989351
JAK1	84598	-0.6616703	0.6616703
JAK2	24514	-1.0800953	1.0800953
JAKMIP1	305434	1.5035542	-1.5035542
JAKMIP3	365380	0.5178917	-0.5178917
JPH3	307916	0.8579227	-0.8579227
JPH4	445271	0.83087945	-0.83087945
JUNB	24517	1.1961887	-1.1961887
KATNBL1	691543	-0.8469377	0.8469377
KBTBD2	312372	-0.7062145	0.7062145
KCNA1	24520	-2.9324117	2.9324117
KCNA4	25469	-1.1384053	1.1384053
KCNB1	25736	-0.9069853	0.9069853
KCNC4	684516	-1.2006938	1.2006938
KCND2	65180	0.85068125	-0.85068125
KCNE2	171138	0.578719	-0.578719
KCNF1	298908	-1.5706642	1.5706642
KCNH3	27150	1.2644694	-1.2644694
KCNH4	114032	2.7187452	-2.7187452
KCNJ12	117052	-0.89960426	0.89960426
KCNJ4	116649	-0.8559248	0.8559248
KCNJ5	29713	-1.992862	1.992862
KCNK1	59324	-0.40121496	0.40121496
KCNK2	170899	2.3072448	-2.3072448
KCNK3	29553	-1.1693228	1.1693228
KCNMB2	294961	-1.3542088	1.3542088
KCNMB4	66016	1.8512713	-1.8512713

KCNN1	54261	1.5103672	-1.5103672
KCNN3	54263	-1.9845036	1.9845036
KCNV1	60326	-1.0496781	1.0496781
KCTD15	499129	0.79915696	-0.79915696
KCTD6	305792	-0.46940184	0.46940184
KDM1A	500569	-0.24557911	0.24557911
KDM4A	313539	0.65337425	-0.65337425
KDSR	360833	0.5790338	-0.5790338
KIF27	246209	0.9834024	-0.9834024
KIFAP3	289168	0.73230666	-0.73230666
KITLG	60427	-1.4576013	1.4576013
KL	83504	-3.6032693	3.6032693
KLC4	316226	1.3974475	-1.3974475
KLF10	81813	-2.4324164	2.4324164
KLF11	313994	2.2614818	-2.2614818
KLF13	499171	0.9078538	-0.9078538
KLF14	312203	-2.951865	2.951865
KLF2	306330	-1.3621902	1.3621902
KLF5	84410	-0.84676117	0.84676117
KLF7	363243	1.1907438	-1.1907438
KLF9	117560	-1.7787198	1.7787198
KLHDC8A	305096	3.1098526	-3.1098526
KLHDC8B	306589	1.5225165	-1.5225165
KLHL14	364823	0.937737	-0.937737
KLHL23	311114	0.75821215	-0.75821215
KLHL24	303803	0.91934127	-0.91934127
KLHL25	293023	0.5308118	-0.5308118
KLHL29	298867	-0.89261734	0.89261734
KLHL36	498957	1.0984741	-1.0984741
KLHL5	305351	0.54883945	-0.54883945
KLHL7	362303	0.7690857	-0.7690857
KMT2E	311968	0.7637932	-0.7637932
KRAS	24525	-0.9396105	0.9396105
LAMB3	305078	-1.7006404	1.7006404
LAMP5	362220	-2.8400226	2.8400226
LANCL2	362375	0.60344684	-0.60344684
LASP1	29278	0.83576113	-0.83576113
LBH	683626	-1.3358486	1.3358486
LCK	313050	1.7219892	-1.7219892
LDAH	313949	0.5557382	-0.5557382
LDB1	309447	0.9988345	-0.9988345
LDLRAD4	679578	-0.878956	0.878956
LEF1	161452	-2.2935052	2.2935052

LENG8	361506	-1.1925628	1.1925628
LEXM	500516	-4.3647046	4.3647046
LGI1	252892	1.1382487	-1.1382487
LHFPL2	294643	-1.3977135	1.3977135
LHX2	296706	1.1847873	-1.1847873
LHX4	360858	-0.7624696	0.7624696
LIMK2	29524	1.1858875	-1.1858875
LINGO3	690755	-0.95841247	0.95841247
LIPA	25055	0.5183348	-0.5183348
LIPG	291437	-1.3595645	1.3595645
LIX1	292381	1.0929558	-1.0929558
LMNA	60374	-1.3383297	1.3383297
LMO1	245979	1.6234221	-1.6234221
LMO2	362176	-1.4608102	1.4608102
LMO4	362051	1.6763406	-1.6763406
LOC100125362	100125362	-0.4107643	0.4107643
LOC100125367	100125367	-0.791909	0.791909
LOC100302465	100302465	-2.2428775	2.2428775
LOC100910945	100910945	1.6606487	-1.6606487
LOC314140	314140	-0.47866273	0.47866273
LOC500877	500877	1.3496305	-1.3496305
LOC680039	680039	-0.6987722	0.6987722
LOC680663	680663	-1.9476616	1.9476616
LOC682102	682102	1.5073361	-1.5073361
LOC688390	688390	1.0159336	-1.0159336
LOC688452	688452	-1.316452	1.316452
LOC688459	688459	-3.1551375	3.1551375
LOC688765	688765	-0.62610525	0.62610525
LPAR1	116744	-3.2895625	3.2895625
LPCAT2	100359680	-0.8129258	0.8129258
LPL	24539	0.85419005	-0.85419005
LRCH1	502020	-0.8170938	0.8170938
LRFN5	314164	1.121322	-1.121322
LRRC10B	309208	-1.2030287	1.2030287
LRRC16B	361041	1.1732049	-1.1732049
LRRC17	502715	-0.84104425	0.84104425
LRRC20	499430	1.2454054	-1.2454054
LRRC26	311803	-2.5454128	2.5454128
LRRC28	361588	0.92711407	-0.92711407
LRRC34	499589	1.666489	-1.666489
LRRC4	641521	-0.7702187	0.7702187
LRRC42	298309	0.95530796	-0.95530796
LRRC55	311171	0.8532531	-0.8532531

LRRC56	365389	1.6173358	-1.6173358
LRRC6	299920	0.894351	-0.894351
LRRC61	500111	1.6301228	-1.6301228
LRRC71	310689	1.3037688	-1.3037688
LRRC8B	305135	-1.5113169	1.5113169
LRRN1	500280	0.79205996	-0.79205996
LRRN3	81514	1.1927134	-1.1927134
LRRTM3	294380	-0.68070257	0.68070257
LRTM2	680883	1.338333	-1.338333
LTBP1	59107	-0.5354814	0.5354814
LUZP1	79428	-0.898674	0.898674
LYPD1	360838	0.981903	-0.981903
LYPLA2	83510	0.44875744	-0.44875744
LYRM7	686506	1.144265	-1.144265
LYRM9	497962	1.8536752	-1.8536752
LYVE1	293186	-1.9220386	1.9220386
LZIC	366507	0.5858828	-0.5858828
LZTFL1	316102	0.6239684	-0.6239684
LZTS1	266711	1.459883	-1.459883
M6PR	312689	-0.55362326	0.55362326
MAF1	315093	1.2809612	-1.2809612
MAFK	246760	0.609026	-0.609026
MAGED2	113947	1.1529831	-1.1529831
MAGEE2	302392	1.4546404	-1.4546404
MAL2	362911	1.1839454	-1.1839454
MAML3	310405	-2.5122805	2.5122805
MAN1A1	294410	-2.6563742	2.6563742
MAN1A2	295319	-1.0048935	1.0048935
MAN1C1	362625	-1.4619563	1.4619563
MAN2A1	25478	-1.7447019	1.7447019
MAP2K3	303200	-1.8593556	1.8593556
MAP2K4	287398	-0.89620936	0.89620936
MAP2K5	29568	0.8882246	-0.8882246
MAP2K6	114495	1.7449335	-1.7449335
MAP3K12	25579	1.0383974	-1.0383974
MAP3K7	313121	-0.4686874	0.4686874
MAP7D1	681287	-0.6060024	0.6060024
MAPK15	286997	2.5421202	-2.5421202
MAPK1IP1	499280	0.7967403	-0.7967403
MAPK3	50689	1.2152724	-1.2152724
MAS1	25153	-1.5918338	1.5918338
MAT2B	683630	0.6810566	-0.6810566
MB21D2	498100	0.7683288	-0.7683288

MBLAC2	365627	0.65403515	-0.65403515
MC5R	25726	-1.2832022	1.2832022
MCCC2	361884	0.7789034	-0.7789034
MCEE	293829	1.3414524	-1.3414524
MCHR1	83567	-1.8251122	1.8251122
MCL1	60430	-0.5577267	0.5577267
MCM5	291885	-0.33948404	0.33948404
MCOLN3	308022	-1.3112947	1.3112947
ME1	24552	-0.7285156	0.7285156
MED15	360743	-0.7941409	0.7941409
MED4	306030	0.919518	-0.919518
MED9	497914	1.1482514	-1.1482514
MEIS2	311311	-1.1404159	1.1404159
MELTF	288038	1.8393661	-1.8393661
MEPCE	304361	0.7829449	-0.7829449
MEST	58827	-0.84213364	0.84213364
METRNL	316842	-1.0411627	1.0411627
METTL18	304928	-0.6321552	0.6321552
MFAP2	313662	0.541674	-0.541674
MFAP3L	306424	-2.319295	2.319295
MFN1	192647	-0.5755169	0.5755169
MFSD13A	309454	0.64404565	-0.64404565
MFSD14B	306687	-0.8376799	0.8376799
MFSD6	301388	0.665314	-0.665314
MFSD9	316356	0.5732246	-0.5732246
MGAT1	81519	0.5889136	-0.5889136
MGAT5B	303693	1.1863223	-1.1863223
MICAL2	365352	-0.9250694	0.9250694
MICU3	364601	-0.73718745	0.73718745
MINK1	303259	-0.5068592	0.5068592
MKRN1	296988	0.7501028	-0.7501028
MLLT3	114510	-0.9280919	0.9280919
MMP10	117061	-4.506247	4.506247
MMP13	171052	-5.4765487	5.4765487
MMP17	288626.chr12.2	-0.74054533	0.74054533
MMP3	171045	-3.3737705	3.3737705
MMS19	171124	0.6874268	-0.6874268
MNT	287521	0.9935537	-0.9935537
MON2	314894	-0.6843731	0.6843731
MORN4	293950	1.3041744	-1.3041744
MOSPD3	288557	0.751187	-0.751187
MPDU1	303244	1.1387473	-1.1387473
MPEG1	64552	0.9923391	-0.9923391

MPPED1	362971	1.5517403	-1.5517403
MPV17L2	290645	1.1072813	-1.1072813
MRM1	363661	-0.40253547	0.40253547
MRPL10	691075	0.88254374	-0.88254374
MRPL14	301250	1.6324209	-1.6324209
MRPL34	290632	1.77923	-1.77923
MRPS12	292758	1.1417923	-1.1417923
MRRF	311903	0.69935894	-0.69935894
MSL3L2	309790	-1.0766865	1.0766865
MSRB1	685059	1.255364	-1.255364
MSTN	29152	1.3471953	-1.3471953
MTERF2	366856	1.3015225	-1.3015225
MTHFD2	680308	-0.6058509	0.6058509
MTMR12	310155	-2.155846	2.155846
MTPAP	307050	-0.4685971	0.4685971
MTSS1	362918	1.1206813	-1.1206813
MTUS2	498136	2.035879	-2.035879
MUSK	81725	-5.454042	5.454042
MUSTN1	290553	-0.9738472	0.9738472
MVK	81727	1.5795974	-1.5795974
MXD4	360961	0.5827962	-0.5827962
MXI1	25701	0.50135267	-0.50135267
MYADML2	303744	2.5961335	-2.5961335
MYBPH	83708	-0.956229	0.956229
MYC	24577	-0.7247556	0.7247556
MYCL	298506	2.5626357	-2.5626357
MYH1	287408	-2.2530081	2.2530081
MYH4	360543	-3.5680916	3.5680916
MYH7	29557	1.8198395	-1.8198395
MYO1E	25484	-2.8696394	2.8696394
MYO9B	25486	-0.92271817	0.92271817
MYRIP	360034	-1.1458832	1.1458832
MYT1L	116668	1.4418831	-1.4418831
N4BP2L1	498131	0.57142353	-0.57142353
NAA40	361718	-0.3950268	0.3950268
NAAA	497009	1.1311554	-1.1311554
NAB2	314910	-2.149525	2.149525
NAGA	315165	0.841882	-0.841882
NAMPT	297508	-1.0371566	1.0371566
NAP1L3	170914	0.95103115	-0.95103115
NAPEPLD	296757	-1.5130607	1.5130607
NARF	360681	1.2755058	-1.2755058
NAT14	361500	1.6346843	-1.6346843

NBR1	303554	0.380894	-0.380894
NCALD	553106	0.59072584	-0.59072584
NCBP1	298075	-0.4643829	0.4643829
NCBP3	360563	-0.32995352	0.32995352
NCOR2	360801	-0.64690495	0.64690495
NCSTN	289231	0.35748908	-0.35748908
NDRG4	64457	0.98793954	-0.98793954
NDST3	295430	-1.1042091	1.1042091
NDST4	362035	1.3141888	-1.3141888
NDUFAF4	362495	-0.99508625	0.99508625
NEDD9	291044	-0.98134995	0.98134995
NEFL	83613	-1.707625	1.707625
NET1	307098	0.47926953	-0.47926953
NETO1	307206	-1.0864983	1.0864983
NEUROD1	29458	1.0511507	-1.0511507
NEUROD2	54276	0.49624836	-0.49624836
NEUROD4	288821	0.92741853	-0.92741853
NFATC1	100361818	-1.4382105	1.4382105
NFIB	29227	0.49739763	-0.49739763
NFKBIE	316241	0.68254673	-0.68254673
NFRKB	315523	-0.55807513	0.55807513
NFX1	313166	-0.36331108	0.36331108
NGB	85382	1.2327213	-1.2327213
NGF	310738	-1.8514184	1.8514184
NHLH2	295327	1.1201491	-1.1201491
NIPSNAP1	360971	1.297048	-1.297048
NIPSNAP3B	313211	1.4569368	-1.4569368
NIT1	289222	0.8626085	-0.8626085
NKAP	298342	1.2555282	-1.2555282
NKD1	364952	1.1703247	-1.1703247
NKIRAS2	287707	0.8874032	-0.8874032
NMBR	25264	-0.97207475	0.97207475
NME7	171566	0.61801004	-0.61801004
NOC3L	361753	-0.6718607	0.6718607
NOCT	310395	-0.38256145	0.38256145
NOG	25495	-0.9140755	0.9140755
NOL10	313981	-0.3505987	0.3505987
NOL3	85383	1.4650979	-1.4650979
NOP2	314969	0.28176972	-0.28176972
NOSIP	292894	1.5772415	-1.5772415
NOTCH4	406162	1.0245112	-1.0245112
NOV	81526	1.4443339	-1.4443339
NPAP60	25497	-0.4197655	0.4197655

NPL	304860	-2.4390168	2.4390168
NPLOC4	140639	-0.4270526	0.4270526
NPTX1	266777	-2.6545897	2.6545897
NPTX2	288475	-3.609085	3.609085
NPY1R	29358	1.4898225	-1.4898225
NPY2R	66024	-1.3297244	1.3297244
NR1D1	252917	0.50560164	-0.50560164
NR1D2	259241	0.41467774	-0.41467774
NR1I3	65035	0.6986578	-0.6986578
NR2F1	81808	1.0604424	-1.0604424
NRF1	312195	-0.95736855	0.95736855
NRIP1	304157	1.0291498	-1.0291498
NRIP3	361625	-0.4573317	0.4573317
NRN1	83834	-2.2758472	2.2758472
NRSN2	689978	1.8389419	-1.8389419
NTF3	81737	1.4229482	-1.4229482
NTM	50864	1.2541375	-1.2541375
NTRK3	29613	-0.6188653	0.6188653
NUAK1	299694	0.9856098	-0.9856098
NUB1	296731	0.8141831	-0.8141831
NUDT14	299346	1.9057776	-1.9057776
NUDT16	363129	1.4501662	-1.4501662
NUDT18	361068	1.2897798	-1.2897798
NUDT6	207120	-1.108387	1.108387
NUTM1	366153	-1.0077661	1.0077661
NVL	289323	-0.4274949	0.4274949
NYAP1	304376	2.2479792	-2.2479792
OGDHL	290566	0.8859162	-0.8859162
OGFOD1	307657	-1.2811823	1.2811823
OLIG2	304103	-0.64515185	0.64515185
OPRL1	29256	1.2142957	-1.2142957
ORAI2	304592	1.5564501	-1.5564501
OS9	362891	0.8949342	-0.8949342
OSBP2	305475	-1.1393825	1.1393825
OSBPL1A	259221	0.4714191	-0.4714191
OSBPL2	296461	0.5098632	-0.5098632
OSBPL6	311129	-1.4557711	1.4557711
OSCAR	292537	-1.0710164	1.0710164
OSCP1	362595	0.8735821	-0.8735821
OTUD3	500572	-1.2368755	1.2368755
OVCA2	497954	0.8804624	-0.8804624
OXTR	25342	-1.5087687	1.5087687
P2RX2	114115	1.5780302	-1.5780302

P2RY1	25265	0.70958847	-0.70958847
P2RY14	171108	-3.1473017	3.1473017
P4HA1	64475	-0.59373695	0.59373695
PACSIN1	29704	-0.7012393	0.7012393
PACSIN2	124461	-0.6784627	0.6784627
PAFAH1B3	114113	1.4949573	-1.4949573
PAK6	296078	-0.74153435	0.74153435
PAK7	311450	0.50949806	-0.50949806
PALMD	310811	1.181603	-1.181603
PAM	25508	-1.4187125	1.4187125
PANX1	315435	-1.0286161	1.0286161
PAPOLA	314417	-0.50829095	0.50829095
PAPPA	313262	-2.3755753	2.3755753
PARD6A	307799	1.2980179	-1.2980179
PATL1	361736	-0.7010471	0.7010471
PAX1	311505	-1.1285974	1.1285974
PBX1	304947	0.7519762	-0.7519762
PCBP4	363133	0.9265226	-0.9265226
PCDH19	317183	1.8977797	-1.8977797
PCDH8	64865	-1.3071531	1.3071531
PCDHA10	116778	0.8411842	-0.8411842
PCDHA12	116779	0.84128857	-0.84128857
PCDHA2	393086	0.8769241	-0.8769241
PCDHA3	116780	0.68591297	-0.68591297
PCDHA4	116741	0.67511904	-0.67511904
PCDHA5	393087	0.6871557	-0.6871557
PCDHA6	393088	0.8123535	-0.8123535
PCDHA7	393089	0.75973856	-0.75973856
PCDHA9	393090	0.5936863	-0.5936863
PCDHGA2	498846	0.62522614	-0.62522614
PCGF2	287662	1.1421169	-1.1421169
PCGF5	681178	-0.94097286	0.94097286
PCSK1	25204	-1.1705414	1.1705414
PCSK4	171085	1.9182475	-1.9182475
PDCD6IP	501083	-0.48611596	0.48611596
PDE10A	63885	-1.4955032	1.4955032
PDE12	306231	-0.8079652	0.8079652
PDE6D	363272	1.5448804	-1.5448804
PDE8A	308776	-3.1037586	3.1037586
PDE9A	191569	-0.9505414	0.9505414
PDGFB	24628	-2.7598	2.7598
PDK1	116551	-0.314967	0.314967
PDP1	54705	-1.9958472	1.9958472

PDSS2	365592	1.6004679	-1.6004679
PDXK	83578	-2.4952135	2.4952135
PDXP	727679	1.1974871	-1.1974871
PDZD8	308000	-0.95551646	0.95551646
PEAK1	315686	-2.5138175	2.5138175
PECAM1	29583	1.6324714	-1.6324714
PENK	29237	-2.9786656	2.9786656
PEO1	309441	1.0950004	-1.0950004
PEX14	64460	1.0501068	-1.0501068
PFKFB3	117276	-2.1468453	2.1468453
PGD	100360180	0.7458782	-0.7458782
PHACTR1	306844	0.7627509	-0.7627509
PHACTR3	362284	0.9712113	-0.9712113
PHEX	25512	-3.0974095	3.0974095
PHF14	500030	0.48963156	-0.48963156
PHF20	311575	0.6254803	-0.6254803
PHLDA1	29380	-1.0356759	1.0356759
PHOSPHO2	295663	-0.24552187	0.24552187
PI16	294312	1.547693	-1.547693
PIANP	312711	1.1342764	-1.1342764
PIEZO1	361430	-1.1661532	1.1661532
PIGM	79112	-1.5399154	1.5399154
PIH1D1	292898	1.2580909	-1.2580909
PIK3C3	65052	1.0280429	-1.0280429
PIK3CB	85243	-0.8965338	0.8965338
PIK3CD	366508	2.0016377	-2.0016377
PIK3IP1	305472	1.0467699	-1.0467699
PITX1	113983	-3.433762	3.433762
PJA2	192256	0.38071728	-0.38071728
PKD2	498328	-1.0872481	1.0872481
PKLR	24651	2.0960937	-2.0960937
PKNOX1	294322	-0.9983487	0.9983487
PKNOX2	680549	0.7530365	-0.7530365
PLA2G4A	24653	-1.7562926	1.7562926
PLAGL1	25157	-0.4995203	0.4995203
PLAUR	50692	-4.892946	4.892946
PLCL1	84587	-2.184532	2.184532
PLCXD2	363781	1.5329875	-1.5329875
PLEKHG5	310999	-1.0774326	1.0774326
PLEKHH2	313866	-2.63918	2.63918
PLK2	83722	-0.8687869	0.8687869
PLK4	310344	-0.31353673	0.31353673
PLPPR3	314614	1.3086072	-1.3086072

PLPPR4	295401	-0.35939395	0.35939395
PLXNA2	289392	-1.0976771	1.0976771
PLXND1	312652	0.890478	-0.890478
PMP22	24660	-0.6559772	0.6559772
PMVK	310645	1.8580743	-1.8580743
PNCK	29660	1.7745042	-1.7745042
PNMA2	305977	1.2323488	-1.2323488
PNMAL1	361515	1.4421582	-1.4421582
PNMAL2	308393	0.617953	-0.617953
PNPO	64533	0.6252789	-0.6252789
PNRC1	286988	1.235424	-1.235424
PODXL	192181	0.6308847	-0.6308847
PODXL2	297433	1.2327952	-1.2327952
POLD1	59294	0.31041476	-0.31041476
POLE4	362385	1.2117931	-1.2117931
POLG	85472	-1.0260669	1.0260669
POLL	361767	1.2602353	-1.2602353
POLR1A	83581	-0.618907	0.618907
POLR3E	361640	-0.6538515	0.6538515
POMK	306549	0.8079559	-0.8079559
POP1	315045	-0.6307121	0.6307121
POPDC2	360718	1.4212865	-1.4212865
POPDC3	641520	-0.6938103	0.6938103
POU4F2	171355	-4.1835046	4.1835046
POU4F3	364855	-3.2644193	3.2644193
POU6F1	116545	-1.1412504	1.1412504
PPEF1	317498	1.2263446	-1.2263446
PPFIA4	140592	1.3249093	-1.3249093
PPHLN1	366975	-0.89656925	0.89656925
PPIF	282819	-0.40926883	0.40926883
PPM1H	314897	-0.7404914	0.7404914
PPP1CA	24668	0.3484464	-0.3484464
PPP1R12A	116670	-0.4603236	0.4603236
PPP1R18	361790	1.5800347	-1.5800347
PPP2R2B	60660	1.0847508	-1.0847508
PPP2R3A	363122	0.93942696	-0.93942696
PPP2R5A	312754	-0.5296165	0.5296165
PPP3CC	171378	0.7928479	-0.7928479
PPP4R3A	314388	-0.26442242	0.26442242
PPRC1	294007	-0.74539727	0.74539727
PRAGMIN	306506	-1.5715748	1.5715748
PRICKLE1	315259	-1.5160234	1.5160234
PRIMA1	690195	-4.5349665	4.5349665

PRKAA1	65248	-0.53383154	0.53383154
PRKAR2A	29699	-1.7279683	1.7279683
PRKCQ	85420	0.6951319	-0.6951319
PRKCZ	25522	0.98278683	-0.98278683
PRKG2	25523	-1.4253035	1.4253035
PRKRA	311130	1.2576514	-1.2576514
PRMT2	499420	1.3451191	-1.3451191
PRR13	363004	0.84493697	-0.84493697
PRR15	312358	-1.0145926	1.0145926
PRRT1	406167	1.2082696	-1.2082696
PRRT2	361651	0.9984324	-0.9984324
PRRT4	500059	1.8222485	-1.8222485
PRSS12	85266	1.6767384	-1.6767384
PRSS23	308807	-2.5837831	2.5837831
PRSS35	315866	-2.0219622	2.0219622
PRUNE	310664	0.6687089	-0.6687089
PSD2	307500	1.2645555	-1.2645555
PSENEN	292788	1.3393663	-1.3393663
PSME3	287716	-0.2551777	0.2551777
PSME4	498433	-0.70672363	0.70672363
PSMG1	288236	1.2615643	-1.2615643
PSTPIP2	307248	-1.2847602	1.2847602
PTAFR	58949	-4.0518913	4.0518913
PTBP1	29497	-0.45423856	0.45423856
PTCHD1	317517	-1.8570472	1.8570472
PTGER4	84023	-2.125272	2.125272
PTGS2	29527	-2.3201041	2.3201041
PTH2R	81753	-2.445413	2.445413
PTPDC1	291022	-0.88963217	0.88963217
PTPN1	24697	-0.3797934	0.3797934
PTPRE	114767	-0.6064726	0.6064726
PTPRH	171125	1.9810252	-1.9810252
PTPRN	116660	-0.87433666	0.87433666
PTPRR	94202.chr7.2	2.134171	-2.134171
PURG	361162	-0.59971136	0.59971136
PUS3	315554	-0.87038594	0.87038594
PUS7	296751	-0.7606243	0.7606243
PVR	25066	-1.5210682	1.5210682
PVRL1	192183	-0.618076	0.618076
PWWP2B	361671	-0.5893987	0.5893987
PXN	360820	-0.61022323	0.61022323
QDPR	64192	1.2607766	-1.2607766
QPCT	313837	1.2014691	-1.2014691

R3HDM1	304763	-0.93854046	0.93854046
RAB11B	79434	0.882444	-0.882444
RAB20	689377	-1.370957	1.370957
RAB27B	84590	-1.699384	1.699384
RAB2B	305853	0.8936181	-0.8936181
RAB30	308821	1.1979102	-1.1979102
RAB33A	317580	2.0812652	-2.0812652
RAB36	690407	1.0716302	-1.0716302
RAB3A	25531	1.0002227	-1.0002227
RAB3D	140665	1.7104299	-1.7104299
RAB43	500249	-1.0554436	1.0554436
RAB9B	367915	0.6534948	-0.6534948
RAD21	314949	0.46561742	-0.46561742
RAI14	294804	-1.4602149	1.4602149
RALGAPA1	56785	-0.4897604	0.4897604
RALGDS	29622	0.93801826	-0.93801826
RALYL	294883	1.3761951	-1.3761951
RAMP2	58966	1.5750382	-1.5750382
RAMP3	56820	2.9852746	-2.9852746
RANBP6	309326	-0.20998384	0.20998384
RAP1B	171337	-0.7228956	0.7228956
RASA1	25676	-0.7775865	0.7775865
RASAL2	304893	-1.3808692	1.3808692
RASGEF1C	360519	1.6070697	-1.6070697
RASGRF1	192213	0.5586639	-0.5586639
RASGRP2	361714	1.6968195	-1.6968195
RASIP1	292912	1.7952964	-1.7952964
RASL11A	304268	-3.7108407	3.7108407
RASSF8	312846	-3.7016206	3.7016206
RAVER1	298705	-0.3390794	0.3390794
RBAK	288489	-0.7255992	0.7255992
RBFA	307235	1.1691409	-1.1691409
RBM11	288321	2.2333245	-2.2333245
RBM12	652928	-0.8207188	0.8207188
RBM24	690139	1.451968	-1.451968
RBM46	310548	1.0298349	-1.0298349
RBMS1	362138	-1.9032842	1.9032842
RBPMS	498642	-0.87195694	0.87195694
RCAN2	140666	-0.7658639	0.7658639
RDH10	353252	-1.5777113	1.5777113
REEP1	362384	0.88295263	-0.88295263
REEP2	682105	0.91989726	-0.91989726
REM2	64626	-0.6479169	0.6479169

RET	24716	-1.3632734	1.3632734
RFXAP	499617	-0.5034862	0.5034862
RGD1304810	306504	-1.4057148	1.4057148
RGD1305014	309029	-0.93383926	0.93383926
RGD1305587	294499	2.1736956	-2.1736956
RGD1306227	310377	0.99300516	-0.99300516
RGD1307443	361244	0.8686419	-0.8686419
RGD1307461	300990	1.7910181	-1.7910181
RGD1308147	307008	-0.3366559	0.3366559
RGD1309028	299265	1.2496943	-1.2496943
RGD1309079	315891	-0.5708009	0.5708009
RGD1310110	361032	1.0895427	-1.0895427
RGD1310769	299207	1.0653373	-1.0653373
RGD1312005	291580	1.0583966	-1.0583966
RGD1560108	499309	0.961564	-0.961564
RGD1561849	500393	-1.1715535	1.1715535
RGD1561931	302396	0.8947085	-0.8947085
RGD1563072	313595	1.8446188	-1.8446188
RGD1564036	497895	1.2878602	-1.2878602
RGD1564804	313551	1.4052379	-1.4052379
RGS12	54292	1.0016395	-1.0016395
RGS14	114705	1.3399516	-1.3399516
RGS16	360857	-2.010148	2.010148
RGS17	308118	-0.698156	0.698156
RGS2	84583	-0.40462452	0.40462452
RGS20	362477	-1.5197148	1.5197148
RGS4	29480	-1.4679604	1.4679604
RGS5	54294	-2.4916008	2.4916008
RHBDF2	303690	2.3448224	-2.3448224
RHBDL1	117025	1.6193181	-1.6193181
RHOQ	85428	-1.2808673	1.2808673
RHOV	171581	2.48307	-2.48307
RHPN2	308516	0.9445657	-0.9445657
RIN1	207119	-2.4847422	2.4847422
RIT1	499652	0.43847728	-0.43847728
RIT2	291713	1.3842981	-1.3842981
RNASEH2A	364974	1.6685215	-1.6685215
RND1	362993	1.7105467	-1.7105467
RNF111	300813	-0.30827323	0.30827323
RNF145	287212	0.68509424	-0.68509424
RNF150	364983	-0.74297404	0.74297404
RNF152	293561	1.9067645	-1.9067645
RNF167	360554	0.67522365	-0.67522365

RNF180	685384	0.40405306	-0.40405306
RNF182	498726	1.5751994	-1.5751994
RNF19B	313806	0.6673586	-0.6673586
RNF217	292188	-0.64998645	0.64998645
RNF34	282845	0.41530994	-0.41530994
RNF39	171387	-0.73396945	0.73396945
RNF5	407784	1.0183594	-1.0183594
RNFT2	304521	0.75005347	-0.75005347
RNGTT	313131	0.5777789	-0.5777789
ROCK2	25537	-0.97574705	0.97574705
RPH3A	171039	0.8633184	-0.8633184
RPS6KA2	117269	1.3743302	-1.3743302
RPS6KA3	501560	-0.86574256	0.86574256
RPS6KA5	314384	0.6330049	-0.6330049
RPS6KA6	317203	-0.65257776	0.65257776
RPS6KB1	83840	-0.6176701	0.6176701
RRAD	83521	-3.126623	3.126623
RRAGD	297960	0.9920717	-0.9920717
RRP8	308911	-0.91041267	0.91041267
RSPO3	498997	1.8153454	-1.8153454
RSRP1	362626	-0.6119179	0.6119179
RT1-S3	294228	-2.6764774	2.6764774
RTF1	366169	0.8068774	-0.8068774
RTFDC1	296410	0.5922399	-0.5922399
RTN4RL1	303311	-1.1240036	1.1240036
RTN4RL2	311169	-1.19438	1.19438
RUNX1	50662	-2.4223049	2.4223049
RUNX1T1	362489	0.62385863	-0.62385863
RUNX2	367218	-1.8867472	1.8867472
RXFP3	294807	-2.7440076	2.7440076
RXRB	361801	1.4743892	-1.4743892
RXRG	83574	2.587212	-2.587212
RYBP	312603	-0.2905089	0.2905089
RYK	140585	-0.49521574	0.49521574
SAMD4A	305826	-2.2564552	2.2564552
SAMD5	365038	-1.5033222	1.5033222
SAMHD1	311580	-0.70444477	0.70444477
SAP30BP	360662	-0.2611519	0.2611519
SATB1	316164	0.87971145	-0.87971145
SC5D	114100	-0.57787454	0.57787454
SCG2	24765	-2.7256134	2.7256134
SCLY	363285	0.79577345	-0.79577345
SCN1A	81574	1.082227	-1.082227

SCN1B	29686	-2.0425715	2.0425715
SCN2A	24766	1.2908763	-1.2908763
SCN3B	245956	0.8881229	-0.8881229
SCN4A	25722	2.258376	-2.258376
SCNN1G	24768	-1.5599666	1.5599666
SCRT2	366229	-2.0606408	2.0606408
SDC1	25216	-1.3037148	1.3037148
SDCBP	83841	-0.3909704	0.3909704
SDCCAG8	305002	1.5519366	-1.5519366
SDHC	289217	0.9934685	-0.9934685
SDK2	360652	-1.9475592	1.9475592
SDR39U1	361044	1.1415017	-1.1415017
SEC13	297522	0.3394574	-0.3394574
SEC16B	89868	-2.1565294	2.1565294
SEC24B	295461	-0.5457721	0.5457721
SEC24D	310843	-1.615148	1.615148
SELE	25544	-1.9188021	1.9188021
SEMA3A	29751	-2.3533933	2.3533933
SEMA3D	246262	-1.2178336	1.2178336
SEMA3E	296789	-0.8667002	0.8667002
SEMA4F	29745	1.4172438	-1.4172438
SEMA4G	361764	1.1764392	-1.1764392
SEMA6B	84609	1.1183448	-1.1183448
SEMA6C	29744	2.2092333	-2.2092333
SEPHS1	291314	-0.42142496	0.42142496
SERPINB2	60325	-4.882518	4.882518
SERPINB8	288937	-2.8327084	2.8327084
SERTAD1	361526	-1.5891567	1.5891567
SERTM1	690333	-0.82669574	0.82669574
SESN1	294518	1.064322	-1.064322
SFI1	305467	0.84867126	-0.84867126
SFMBT1	58967	-0.62853	0.62853
SFMBT2	307106	-1.643639	1.643639
SFRP4	89803	-1.0778743	1.0778743
SGCA	303468	-1.9697973	1.9697973
SGMS1	353229	-0.8720232	0.8720232
SH2B2	114203	1.1817917	-1.1817917
SH2D3C	362111	3.8986006	-3.8986006
SH3BP1	300067	-0.7575102	0.7575102
SH3BP5	117186	1.111334	-1.111334
SH3GLB1	292156	-0.39560103	0.39560103
SHC4	679845	-2.4207256	2.4207256
SHISA5	301013	0.8572069	-0.8572069

SHQ1	297483.chr4.1	-0.90175235	0.90175235
SHROOM2	317435	0.94060034	-0.94060034
SHROOM4	317391	-3.285885	3.285885
SHTN1	292139	0.7538315	-0.7538315
SIAH3	692004	-2.7351675	2.7351675
SIDT1	288109	0.9055039	-0.9055039
SIK1	59329	-1.636797	1.636797
SIK3	684112	-1.1976771	1.1976771
SIL1	291673	1.5942905	-1.5942905
SIM1	309888	-2.920881	2.920881
SIN3A	363067	0.41163677	-0.41163677
SIPA1L2	361442	-0.8146569	0.8146569
SIRT3	293615	1.1229125	-1.1229125
SIRT5	306840	1.8049828	-1.8049828
SKP2	294790	-1.1556857	1.1556857
SLAIN2	305310	-0.46348232	0.46348232
SLC16A14	316578	-2.186105	2.186105
SLC16A8	65200	-1.2989899	1.2989899
SLC17A5	363103	0.6097403	-0.6097403
SLC17A6	84487	-0.9674586	0.9674586
SLC18A2	25549	-0.6991742	0.6991742
SLC1A6	84012	1.3026911	-1.3026911
SLC22A1	24904	4.297857	-4.297857
SLC22A3	29504	2.5468235	-2.5468235
SLC24A4	314396	-0.37487805	0.37487805
SLC25A14	85263	0.5005022	-0.5005022
SLC25A20	117035	0.8498117	-0.8498117
SLC25A37	306000	-1.8937051	1.8937051
SLC25A39	360636	1.3247854	-1.3247854
SLC27A2	65192	1.1857318	-1.1857318
SLC28A3	140944	1.9427401	-1.9427401
SLC2A3	25551	-1.5491256	1.5491256
SLC30A10	289353	-1.2617816	1.2617816
SLC30A3	366568	-0.72683805	0.72683805
SLC30A4	64469	-0.8386116	0.8386116
SLC35B4	296969	0.3722925	-0.3722925
SLC35D1	298280	-1.6176689	1.6176689
SLC35D3	308717	-1.6887423	1.6887423
SLC35E3	362883	-0.41176802	0.41176802
SLC35F5	288993	0.37466466	-0.37466466
SLC37A4	29573	1.1339908	-1.1339908
SLC40A1	170840	-2.2319286	2.2319286
SLC41A2	362861	-1.2773671	1.2773671

SLC4A11	311423	-1.7083405	1.7083405
SLC4A7	117955	-2.0753016	2.0753016
SLC5A10	303205	-1.8730152	1.8730152
SLC5A3	114507	-1.519374	1.519374
SLC5A8	500820	-1.7296529	1.7296529
SLC6A15	282712	0.711413	-0.711413
SLC6A17	613226	-1.3041549	1.3041549
SLC6A7	117100	1.1860822	-1.1860822
SLC6A8	50690	-0.67422664	0.67422664
SLC9A2	24783	-1.3528452	1.3528452
SLC9B2	679958	1.9674516	-1.9674516
SLCO2A1	24546	-4.5949497	4.5949497
SLCO3A1	140915	-1.3849361	1.3849361
SLCO4A1	171144	1.2394837	-1.2394837
SLITRK4	302473	0.9764185	-0.9764185
SMAD3	25631	-2.0338812	2.0338812
SMAGP	300236	-2.016632	2.016632
SMARCA2	361745	0.8919621	-0.8919621
SMARCD3	296732	1.4601693	-1.4601693
SMIM17	499067	1.050431	-1.050431
SMIM20	501923	1.1410125	-1.1410125
SMIM3	286910	0.81856567	-0.81856567
SMOC2	292401	2.2719147	-2.2719147
SMPD3	94338	1.0461674	-1.0461674
SMPDL3B	362619	-2.7852929	2.7852929
SMR3B	24867	-1.3570703	1.3570703
SMTN	289734	-1.9741095	1.9741095
SMYD5	312503	0.71387887	-0.71387887
SNCA	29219	1.2924238	-1.2924238
SNN	29140	0.44895124	-0.44895124
SNRNP40	313056	0.3734343	-0.3734343
SNX9	683687	-1.3578744	1.3578744
SOAT1	81782	-1.5339559	1.5339559
SOCS3	89829	-1.716651	1.716651
SOGA3	292199	0.7784561	-0.7784561
SORBS2	114901	-1.709716	1.709716
SOX4	364712	1.2455767	-1.2455767
SP7	300260	-4.5945225	4.5945225
SPAST	362700	0.5576073	-0.5576073
SPATA2L	498963	-0.4327329	0.4327329
SPCS3	680782	-0.78490406	0.78490406
SPHK1	170897	-3.063561	3.063561
SPHKAP	316561	1.4794879	-1.4794879

SPIRE1	307348	-0.45116532	0.45116532
SPIRE2	307925	1.3488461	-1.3488461
SPNS2	100270678	-0.66034466	0.66034466
SPOP	287643	1.0444767	-1.0444767
SPRED3	308478	-1.8490422	1.8490422
SPRR1A	499660	-5.059616	5.059616
SPRTN	292101	-1.1680199	1.1680199
SPRY2	306141	-1.6108091	1.6108091
SPRY4	291610	-2.687808	2.687808
SPSB1	313722	-2.0201378	2.0201378
SQLE	29230	0.30901468	-0.30901468
SRD5A1	24950	0.84864455	-0.84864455
SRPK1	361811	-0.41135523	0.41135523
SRPRA	315548	-0.29907146	0.29907146
SRRM3	685890	0.9302858	-0.9302858
SRSF9	288701	0.4320581	-0.4320581
SRXN1	296271	-4.2581544	4.2581544
SS18	361295	-0.85127825	0.85127825
SSR1	361233	-0.19632502	0.19632502
SSTR2	54305	-0.9175802	0.9175802
SSTR3	171044	-3.1048226	3.1048226
SSTR4	25555	-1.9505814	1.9505814
ST18	266680	-1.01843	1.01843
ST3GAL2	64442	0.8283027	-0.8283027
ST3GAL3	64445	0.82038593	-0.82038593
ST6GAL1	25197	-0.36678654	0.36678654
ST6GALNAC6	407765	0.9609258	-0.9609258
ST8SIA5	364901	-2.471564	2.471564
STAC2	363674	-0.914047	0.914047
STAMBP	171565	0.9105669	-0.9105669
STC2	63878	-1.6325964	1.6325964
STEAP1	297738	-1.2303724	1.2303724
STEAP2	312052	-0.30343154	0.30343154
STIM1	361618	0.4927793	-0.4927793
STK26	317589	-3.2381546	3.2381546
STOML1	300748	1.3919854	-1.3919854
STRADA	303605	0.83367527	-0.83367527
STT3B	363160	-0.8150197	0.8150197
STX11	292483	-3.5040588	3.5040588
SULT2B1	292915	1.1133765	-1.1133765
SUN1	360773	-0.8405576	0.8405576
SUOX	81805	0.60807633	-0.60807633
SV2A	117559	1.0175225	-1.0175225

SV2B	117556	1.2700803	-1.2700803
SV2C	29643	-3.5015628	3.5015628
SVBP	362578	1.7390351	-1.7390351
SVOP	171442	1.1907222	-1.1907222
SYK	25155	-0.5262172	0.5262172
SYNJ2	84018	-1.7209162	1.7209162
SYNPO	60324	-1.3905915	1.3905915
SYPL2	362018	-2.3790407	2.3790407
SYT10	60567	-3.5787656	3.5787656
SYT12	191595	-0.9574822	0.9574822
SYT13	80977	0.60884726	-0.60884726
SYT3	25731	1.6051745	-1.6051745
SYT6	60565	-0.7595564	0.7595564
SYT9	60564	0.75644463	-0.75644463
SYTL5	302538	-1.7463497	1.7463497
TAC1	24806	-1.1776572	1.1776572
TACC1	306562	-1.1586126	1.1586126
TACR2	25007	-2.0917666	2.0917666
TACR3	24808	-2.047815	2.047815
TADA2B	289717	-0.44116637	0.44116637
TAF12	682902	-0.5072301	0.5072301
TAGAP	308097	-1.8094736	1.8094736
TARS	294810	-0.57766813	0.57766813
TAS1R3	170634	-1.8621123	1.8621123
TBC1D14	360956	0.71468055	-0.71468055
TBC1D17	292886	1.2522173	-1.2522173
TBC1D25	302552	0.55837303	-0.55837303
TBC1D9	304645	0.7860574	-0.7860574
TBCK	295446	0.8337724	-0.8337724
TBX21	303496	-1.7847887	1.7847887
TCEAL1	302593	0.9830134	-0.9830134
TCP11L2	314683	0.6612161	-0.6612161
TCTN1	304486	0.77660185	-0.77660185
TDRD6	316254	2.2980094	-2.2980094
TEAD1	361630	-2.510715	2.510715
TEF	29362	0.608276	-0.608276
TEKT2	298532	1.7960329	-1.7960329
TENM3	306451	0.9096696	-0.9096696
TEX264	300988	1.285548	-1.285548
TFAP4	360482	0.829667	-0.829667
TGFB1	59086	-0.9455964	0.9455964
TGFBR2	81810	-2.4523053	2.4523053
TGIF1	316742	-1.1005571	1.1005571

THBD	83580	-1.7839245	1.7839245
THNSL1	498805	0.4096687	-0.4096687
THRB	24831	-1.8771453	1.8771453
THSD7A	500032	0.6431325	-0.6431325
TIGAR	502894	-0.43789047	0.43789047
TIMP1	116510	-1.594023	1.594023
TLK2	303592	-0.35235876	0.35235876
TM4SF1	295061	-1.8866996	1.8866996
TMCC2	305095	1.1049625	-1.1049625
TMCC3	314751	-0.9580081	0.9580081
TMEM106B	312132	0.31767172	-0.31767172
TMEM106C	315286	1.0677006	-1.0677006
TMEM107	691750	1.5588582	-1.5588582
TMEM141	499755	1.8384227	-1.8384227
TMEM150C	360916	1.5865021	-1.5865021
TMEM163	360839	-0.7065779	0.7065779
TMEM179	314472	1.2138616	-1.2138616
TMEM182	501129	-2.9876928	2.9876928
TMEM184B	362959	0.3989972	-0.3989972
TMEM19	299800	0.858972	-0.858972
TMEM200C	501201	1.0343357	-1.0343357
TMEM202	691306	-1.2168857	1.2168857
TMEM218	300516	1.4755671	-1.4755671
TMEM229B	503035	0.8901293	-0.8901293
TMEM246	362518	1.4409344	-1.4409344
TMEM25	689172	1.2348101	-1.2348101
TMEM39A	288092	0.632154	-0.632154
TMEM41A	681708	-0.71854585	0.71854585
TMEM42	363171	1.2895105	-1.2895105
TMEM45A	680866	-2.076463	2.076463
TMEM55B	364298	0.9689839	-0.9689839
TMEM65	500874	-0.7801371	0.7801371
TMEM68	312946	0.97808397	-0.97808397
TMEM8A	303004	0.98735017	-0.98735017
TMIE	501061	2.1490898	-2.1490898
TMOD1	25566	-0.79898345	0.79898345
TMPRSS6	315388	-2.1276395	2.1276395
TMTC4	290501	1.451589	-1.451589
TNFAIP1	287543	0.38224366	-0.38224366
TNFRSF11A	498206	0.8378635	-0.8378635
TNFRSF12A	302965	-1.5811874	1.5811874
TNFRSF25	500592	-2.0133827	2.0133827
TNIP1	363599	0.5466149	-0.5466149

TNIP2	305451	-0.9859405	0.9859405
TNRC18	304302	0.62138903	-0.62138903
TOR1AIP1	246314	-0.38566485	0.38566485
TOX3	291908	1.0046704	-1.0046704
TP73	362675	-1.8165276	1.8165276
TPBG	83684	-1.2230638	1.2230638
TPD52L3	293894	2.1558177	-2.1558177
TPH1	24848	-1.759721	1.759721
TPK1	680668	1.1043934	-1.1043934
TPM4	24852	-0.78833544	0.78833544
TPPP3	291966	2.493314	-2.493314
TRAPPC2B	100910318	1.4842247	-1.4842247
TRAPPC3	362599	1.1807364	-1.1807364
TRERF1	316219	0.6702241	-0.6702241
TRIB1	78969	-2.3380017	2.3380017
TRIB2	313974	-0.85800225	0.85800225
TRIM23	81002	0.6327717	-0.6327717
TRIM45	295323	1.0136446	-1.0136446
TRIM9	155812	-1.8680766	1.8680766
TRIQK	500413	1.4419904	-1.4419904
TRMT10C	304012	-0.3914864	0.3914864
TRMT6	311441	-0.88919955	0.88919955
TRPC1	89821	0.63926923	-0.63926923
TRPC4	84494	1.104643	-1.104643
TRPC6	89823	-3.409288	3.409288
TRPC7	282822	1.1666611	-1.1666611
TRPM4	171143	1.397249	-1.397249
TRPT1	293704	1.089093	-1.089093
TRPV6	114246	1.3203087	-1.3203087
TSC22D1	498545	0.61734474	-0.61734474
TSPAN17	306771	1.5900029	-1.5900029
TSPAN2	64521	0.8337795	-0.8337795
TSPAN5	362048	-0.75329566	0.75329566
TSPYL1	29544	0.55921113	-0.55921113
TTC19	691506	-0.5289188	0.5289188
TTC9	500689	-0.55293614	0.55293614
TTLL1	362969	1.3057308	-1.3057308
TTPAL	296349	-1.0306127	1.0306127
TUBA8	500377	-1.2659577	1.2659577
TUBB4B	296554	0.42896006	-0.42896006
TUBG2	680991	1.3486153	-1.3486153
TUBGCP3	306599	-0.40801	0.40801
TUSC5	360576	-2.4632652	2.4632652

TWIST1	85489	-0.961156	0.961156
TXNIP	117514	1.4114915	-1.4114915
TXNRD1	58819	-0.74920535	0.74920535
TYMS	29261	-0.8746929	0.8746929
TYR	308800	2.1056445	-2.1056445
TYRO3	25232	-0.3947916	0.3947916
UAP1	498272	-0.38090712	0.38090712
UBASH3B	315579	-2.500164	2.500164
UBE2D1	361831	1.0117896	-1.0117896
UHRF1BP1L	363009	-0.48036596	0.48036596
ULK2	303206	0.4279266	-0.4279266
UNC119	29402	1.4818367	-1.4818367
UNC5B	60630	-2.809945	2.809945
UOX	114768	-2.7210045	2.7210045
USF1	83586	0.7900194	-0.7900194
USO1	56042	-0.34504065	0.34504065
USP11	408217	0.580222	-0.580222
USP20	311856	1.0782478	-1.0782478
USP28	315639	-0.79407537	0.79407537
USP46	289584	0.6767605	-0.6767605
USP49	316211	0.6756008	-0.6756008
UST	361450	-1.3118315	1.3118315
UTP15	310019	-0.9198079	0.9198079
UTP20	314713	-0.92448187	0.92448187
VAC14	307842	0.91569674	-0.91569674
VASP	361517	-1.2618965	1.2618965
VCL	305679	-1.6796203	1.6796203
VEGFA	83785	-1.0660167	1.0660167
VGF	29461	-3.8885574	3.8885574
VGLL4	297523	-0.45178235	0.45178235
VIPR1	24875	-2.1832316	2.1832316
VKORC1	309004	1.4050825	-1.4050825
VOPP1	362374	0.8389173	-0.8389173
VPS25	681059	0.9774818	-0.9774818
VPS53	287535	0.69485587	-0.69485587
VSTM2B	361560	-0.8955742	0.8955742
VWA1	298683	2.2382662	-2.2382662
VWA5B2	303812	1.0999229	-1.0999229
VWC2	501231	-0.7515774	0.7515774
VWC2L	501160	2.1847382	-2.1847382
WASH1	367328	1.4061364	-1.4061364
WDHD1	305827	-0.54014874	0.54014874
WDR1	360950	-0.49472558	0.49472558

WDR34	296618	1.4017438	-1.4017438
WDR43	362703	-0.6477642	0.6477642
WDR45	302559	1.1314515	-1.1314515
WDR45B	360682	-0.67207795	0.67207795
WDR6	301007	0.90195644	-0.90195644
WFS1	83725	-0.6478758	0.6478758
WIPI2	288498	-0.4373512	0.4373512
WISP1	65154	-2.8569026	2.8569026
WNT6	316526	-2.9822986	2.9822986
WNT7A	114850	0.54971087	-0.54971087
WSCD1	287466	-0.6364453	0.6364453
WSCD2	360824	1.4729046	-1.4729046
XKR4	297801	0.6514431	-0.6514431
XKR7	311549	-1.9481895	1.9481895
XKR8	313033	-1.112087	1.112087
XPO4	290280	-0.98097914	0.98097914
XRCC4	309995	1.1296892	-1.1296892
YPEL3	293491	1.6609019	-1.6609019
YPEL5	298792	0.9776608	-0.9776608
ZBTB16	353227	1.1991673	-1.1991673
ZBTB22	309630	0.99966913	-0.99966913
ZBTB46	311718	-1.4119025	1.4119025
ZBTB5	298084	0.5326453	-0.5326453
ZBTB9	294289	-0.53306437	0.53306437
ZC2HC1A	310244	1.0619466	-1.0619466
ZC3H18	292067	-0.47602633	0.47602633
ZC3H8	311414	0.7709499	-0.7709499
ZC4H2	367838	1.3166186	-1.3166186
ZDHHC14	499014	-0.5482368	0.5482368
ZDHHC20	305923	-0.734937	0.734937
ZDHHC5	362156	-0.77307737	0.77307737
ZFAND2B	363253	1.1858829	-1.1858829
ZFAND6	293067	1.0159383	-1.0159383
ZFAT	362925	-0.368832	0.368832
ZFP217	311764	-2.161255	2.161255
ZFP238	64619	1.1051159	-1.1051159
ZFP358	360754	0.9876498	-0.9876498
ZFP362	297879	0.492345	-0.492345
ZFP395	305972	0.5507338	-0.5507338
ZFP414	299647	1.2685622	-1.2685622
ZFP467	500110	0.87794447	-0.87794447
ZFP513	313913	0.50401187	-0.50401187
ZFP575	308430	1.4536451	-1.4536451

ZFP672	303165	-0.67905396	0.67905396
ZFP697	295310	-1.3314832	1.3314832
ZFP709	266773	-0.7732385	0.7732385
ZFP775	312309	1.252074	-1.252074
ZFP786	100158223	0.84110814	-0.84110814
ZFP90	498945	1.1531198	-1.1531198
ZFPM1	691504	-1.1701488	1.1701488
ZFYVE1	299188	0.5481313	-0.5481313
ZFYVE19	499871	1.4545959	-1.4545959
ZFYVE9	313477	-0.6973551	0.6973551
ZGPAT	296478	0.540302	-0.540302
ZMIZ1	361103	-1.3804868	1.3804868
ZMYND8	296374	0.6084991	-0.6084991
ZNRF2	362367	0.7356772	-0.7356772
ZPBP2	363676	0.8807924	-0.8807924
ZW10	363059	-0.37213176	0.37213176
ZYX	114636	-0.7388414	0.7388414

Epilepsy-linked V+B v CTRL

GENE SYMBOL
GALR1
ARC
PLAUR
EGR2
FOSB
GAL
NPTX2
LAMP5
MMP9
EGR4
PRIMA1
EGR1
GRASP
SCG2
ADRA1D
GADD45B
DUSP5
KLF10
KCNA1
SCN1B
DUSP6
HTR1B
EGR3
PTGS2
GFRA1
HTR1F
INHBA
FGF2
NGF
RAG1
SERTAD1
BAG3
RGS4
NRN1
CITED2
THRB
TRIB1
PLCL1
PDP1
FOS
ADRA1B
LAMB3
RGS16

TAC1
BDNF
BAIAP2
HOMER1
GDNF
PRICKLE1
IFRD1
CREM
ADRA1A
CDKN1A
BHLHE40
SOCS3
PNOC
HAS1
KCNV1
TRIM9
IER2
SV2C
SPRY2
HSPA1A/HSPA1B
OPRD1
SLC2A3
PLK2
NPY2R
GADD45G
SPNS2
ERRFI1
JUNB
GRIN2A
PTPRN
DNAJB5
DUSP2
POLG
ADM
FGF7
CYR61
NPY
SSTR2
CALCRL
JUN
CAMKK1
KCNB1
CH25H
HCN1
GADD45A

NR4A1
METRNL
HTR7
TYRO3
DIAPH1
SPATA5
ZFP36
RGS2
MYC
GABRQ
NAMPT
GFRA2
SH3GL1
CACNB4
ADCYAP1
LINGO1
KCNA4
STAM
PACSIN1
PLK3
MCM6
FGF22
GRIN1
NRG1
LDLR
GRIN3B
PLAT
GNA11
ADARB1
NR4A3
SIGMAR1
HES1
SLC2A1
HNRNPU
ARHGEF7
PPP1R1B
FYN
PNKP
ARHGEF9
RAPGEF3
MCL1
AARS
KCNK1
WDR26
FKBP1A

PMP22
USF1
TRIM3
TWNK
DEPDC5
SCLY
ARRDC3
ITPR1
SV2A
ABHD6
KCNJ9
SLC4A10
STRADB
FGF13
STRADA
PRRT2
BCL2L11
SLC37A4
CRYM
BAD
TEF
DEAF1
SLITRK3
NGLY1
CNR1
MBNL2
PNPO
BEND5
CXCR4
NR2F1
CNNM2
ST3GAL3
GABRD
KCNH3
BID
SCN1A
SCN3B
ANKRD6
ST8SIA4
GABRA1
AKAP5
DBP
BBC3
NTF3
KCNMB4

TRPC7
PSEN2
PCDH19
ELAVL4
SCN2A
CHRNA5
LGI1
CNTN2
SV2B
SCN4A
GABRG2
OPRK1
IGSF9
GRM1
NEUROD1
KCNK2

Epilepsy-linked WP+B and RX2+B reversal gene list

Gene Syml	ID
KCNK2	170899
SCN4A	25722
IGSF9	304982
CYR61	83476
PCDH19	317183
KCNMB4	66016
GABRD	29689
GRM1	24414
BBC3	317673
CRYM	117024
ELAVL4	432358
CNTN2	25356
NTF3	81737
BEND5	362564
HSPA1A/H	294254
BAD	64639
SCN2A	24766
SV2B	117556
KCNH3	27150
JUNB	24517
CHRNA5	25102
TRPC7	282822
GABRG2	29709
LGI1	252892
SLC37A4	29573
TWNK	309441
SCN1A	81574
NR2F1	81808
NEUROD1	29458
ANKRD6	500430
FGF13	84488
SV2A	117559
DBP	24309
PRRT2	361651
SCN3B	245956
BID	64625
STRADA	303605
CXCR4	60628
ST3GAL3	64445
USF1	83586
PNPO	64533
TEF	29362
DEPDC5	305464

CNR1	25248
ABHD6	305795
CNNM2	294014
TYRO3	25232
KCNK1	59324
RGS2	84583
MCL1	60430
CAMKK1	60341
PMP22	24660
SPNS2	1E+08
HTR7	65032
DIAPH1	307483
PACSIN1	29704
MYC	24577
DNAJB5	313811
ADM	25026
PLK2	83722
PTPRN	116660
IFRD1	29596
KCNB1	25736
GABRQ	65187
SSTR2	54305
HCN1	84390
POLG	85472
NAMPT	297508
ERRFI1	313729
METRNL	316842
KCNV1	60326
KCNA4	25469
TAC1	24806
CH25H	309527
CALCRL	25029
CACNB4	58942
EGR2	114090
NPY2R	66024
CREM	25620
RGS4	29480
GADD45B	299626
PRICKLE1	315259
BDNF	24225
SLC2A3	25551
SERTAD1	361526
CITED2	114490
SPRY2	306141
LAMB3	305078

BAIAP2	117542
DUSP6	116663
SOCS3	89829
ADRA1B	24173
HOMER1	29546
NGF	310738
TRIM9	155812
GDNF	25453
THRB	24831
PDP1	54705
RGS16	360857
SCN1B	29686
PLCL1	84587
BHLHE40	79431
NRN1	83834
PTGS2	29527
TRIB1	78969
INHBA	29200
DUSP5	171109
KLF10	81813
ADRA1D	29413
GFRA1	25454
EGR3	25148
SCG2	24765
HAS1	282821
LAMP5	362220
BAG3	293524
GRASP	192254
KCNA1	24520
GAL	29141
HTR1F	60448
HTR1B	25075
GRIN2A	24409
SV2C	29643
NPTX2	288475
ADRA1A	29412
ARC	54323
FGF2	54250
PLAUR	50692
GALR1	50577

Top WP+B and RX2+B in network by category

Synaptic Plasticity Group	Entrez ID
ADCY8	29241
ADORA1	29290
ADORA2A	25369
ARC	54323
BAIAP2	117542
BDNF	24225
CACNB4	58942
CDK5	140908
CNR1	25248
CNTN2	25356
CREM	25620
DRD5	25195
EFNA4	310643
EFNB3	360546
EGR2	114090
EGR3	25148
ERCC1	292673
GRIN2A	24409
GRIP1	84016
GRM1	24414
HOMER1	29546
HRH1	24448
IGF1	24482
INHBA	29200
JPH3	307916
JPH4	445271
JUNB	24517
KCNH3	27150
KLF9	117560
KLF10	81813
KRAS	24525
LZTS1	266711
NGF	310738
NPTX2	288475
NTF3	81737
NTRK3	29613
PCDH8	64865
PLK2	83722
PPP1CA	24668
PRKAR2A	29699

RAB3A	25531
RASGRF1	192213
RGS2	84583
SNCA	29219
SYNPO	60324
TIMP1	116510
VGF	29461

**Receptors and ion channels group**	**Entrez ID**
ADGRB1	362931
ADGRB3	301309
ADGRE5	361383
ADORA1	29290
ADORA2A	25369
ADRA1A	29412
ADRA1B	24173
ADRA1D	29413
AGTR1	24180
ANO1	309135
CACNB3	25297
CALCRL	25029
CCKAR	24889
CCKBR	25706
CHRNA5	25102
CNR1	25248
DRD5	25195
EDNRA	24326
F2R	25439
FFAR4	294075
FZD1	58868
FZD4	64558
GABARAPL1	689161
GABRD	29689
GABRG2	29709
GABRQ	65187
GALR1	50577
GCGR	24953
GPR3	266769
GPR4	308408
GPR12	80840
GPR26	192153
GPR37	117549
GPR45	301372

GPR50	117097
GPR61	310780
GPR63	297952
GPR68	314386
GPR83	140595
GPR85	64020
GPR88	64443
GPR101	317608
GPR139	293545
GPR156	260430
GPR158	291352
GPRC5A	312790
GPRC5C	287805
GRIN2A	24409
GRIP1	84016
GRM1	24414
GRM6	24419
HCN1	84390
HCN3	114245
HRH1	24448
HRH3	85268
HTR7	65032
HTR1B	25075
HTR1F	60448
KCNA1	24520
KCNA4	25469
KCNB1	25736
KCNC4	684516
KCND2	65180
KCNE2	171138
KCNF1	298908
KCNH3	27150
KCNH4	114032
KCNJ4	116649
KCNJ5	29713
KCNJ12	117052
KCNK1	59324
KCNK2	170899
KCNK3	29553
KCNMB2	294961
KCNMB4	66016
KCNN1	54261
KCNN3	54263

KCNV1	60326
KCTD6	305792
KCTD15	499129
LPAR1	116744
MAS1	25153
MC5R	25726
MCHR1	83567
NMBR	25264
NPY1R	29358
NPY2R	66024
OPRL1	29256
OXTR	25342
P2RY1	25265
P2RY14	171108
PTAFR	58949
PTGER4	84023
RXFP3	294807
SCN1A	81574
SCN1B	29686
SCN2A	24766
SCN3B	245956
SCN4A	25722
SCNN1G	24768
SSTR2	54305
SSTR3	171044
SSTR4	25555
TACR2	25007
TACR3	24808
TAS1R3	170634
VIPR1	24875

**Proliferation Group**	**Entrez ID**
AATK	690853
ADCY7	84420
ADCY8	29241
ADORA2A	25369
AKT2	25233
ANKRD6	500430
APBA2	83610
APBB1	29722
ARHGAP17	63994
ARHGEF25	314904
BAIAP2	117542

BDNF	24225
CABLES1	307585
CACNB3	25297
CACNB4	58942
CAMKK1	60341
CAT	24248
CAV1	25404
CCND1	58919
CDH7	29162
CDH15	361432
CDK5	140908
CFLAR	117279
CLASP2	114514
CNP	25275
CNR1	25248
CNTN2	25356
COL4A1	290905
COL5A1	85490
CSPG5	50568
CX3CL1	89808
CXCR4	60628
DISC1	307940
DLC1	58834
DOK4	361364
DOK5	502694
DPYSL2	25416
EEA1	314764
EFNB3	360546
EGR3	25148
EIF5A	287444
ELAVL4	432358
EML1	362783
ESR1	24890
ETV4	360635
ETV5	303828
EXOC5	60627
EZR	54319
FEZ1	81730
FGF2	54250
FGF3	170633
FGF8	29349
FGF9	25444
FGF13	84488

FGFR1	79114
FKBP5	361810
FZR1	314642
GAL	29141
GAS7	85246
GDNF	25453
GFRA1	25454
GPM6A	306439
GPR3	266769
GPR12	80840
GRASP	192254
GRK5	59075
GRN	29143
IFNG	25712
IGF1	24482
IGSF9	304982
IL6R	24499
IRS2	29376
IRX6	307715
ITGA6	114517
JAK1	84598
JAK2	24514
KCNA1	24520
KITLG	60427
KL	83504
KRAS	24525
LGI1	252892
LPAR1	116744
MAFK	246760
MAML3	310405
MAP2K3	303200
MAP2K4	287398
MAP2K5	29568
MAP2K6	114495
MAPK3	50689
MAPK15	286997
MAR8	312656
MFN1	192647
MUSK	81725
NEUROD4	288821
NFATC1	100361818
NGB	85382
NGF	310738

NOG	25495
NPTX1	266777
NR1D1	252917
NRF1	312195
NRN1	83834
NTF3	81737
NTRK3	29613
PAK5	311450
PAK6	296078
PDGFB	24628
PDP1	54705
PIK3C3	65052
PIK3CB	85243
PIK3CD	366508
PJA2	192256
PLAGL1	25157
PLCL1	84587
PLPPR4	295401
POLR3E	361640
POU4F2	171355
POU4F3	364855
PRAG1	306506
PRKAA1	65248
PRKAR2A	29699
PRKCQ	85420
PRKCZ	25522
PTPDC1	291022
PXN	360820
RAB33A	317580
RALGDS	29622
RAP1B	171337
RASA1	25676
RASGRF1	192213
RET	24716
RHOQ	85428
RHOV	171581
RIT1	499652
RIT2	291713
RND1	362993
ROCK2	25537
RTN4RL2	311169
RXRB	361801
RXRG	83574

SCN1B	29686
SDC1	25216
SEMA3A	29751
SEMA3D	246262
SH2D3C	362111
SMARCD3	296732
SOCS3	89829
SPAST	362700
SPRR1A	499660
SPRY2	306141
SS18	361295
SSTR3	171044
SYK	25155
TGFB1	59086
TGFBR2	81810
TNFRSF12A	302965
TP73	362675
Tpm4	24852
TRPC1	89821
TWIST1	85489
VCL	305679
VEGFA	83785
VGF	29461
ZBTB18	64619

**Neurogenesis group**	**Entrez ID**
AATK	690853
ABI1	79249
ACSL4	113976
ACVR1	79558
ADAP1	171097
ADCY7	84420
ADCY8	29241
ADGRB1	362931
ADGRB3	301309
ADGRE5	361383
ADM	25026
ADORA2A	25369
AIPL1	59110
AKT2	25233
ANAPC2	296558
ANKRD6	500430
APBA2	83610

APBB1	29722
ARC	54323
AREG	29183
ARHGAP17	63994
ARHGEF25	314904
ARHGEF28	361882
ARPP21	363153
BAIAP2	117542
BDNF	24225
BHLHE22	365748
BHLHE23	499952
BID	64625
BLOC1S6	317630
BRINP3	286901
CABLES1	307585
CACNB3	25297
CACNB4	58942
CAMK1G	171358
CAMKK1	60341
CAT	24248
CAV1	25404
CBLN1	498922
CBLN2	291388
CCKAR	24889
CCND1	58919
CDH7	29162
CDH15	361432
CDK5	140908
CDK5RAP1	252827
CDK5RAP3	80278
CFLAR	117279
Chrm2	81645
CLASP2	114514
CLN8	306619
CNP	25275
CNR1	25248
CNTFR	313173
CNTN2	25356
COL4A1	290905
COL5A1	85490
CREM	25620
CSPG5	50568
CTSF	361704

CUX2	288665
CX3CL1	89808
CXCR4	60628
CYP26B1	312495
CYR61	83476
DDIT4	140942
DIAPH1	307483
DISC1	307940
DLC1	58834
DLG1	25252
DLK1	114587
DOK4	361364
DOK5	502694
DPYSL2	25416
EBF1	116543
EBF3	361668
ECEL1	60417
EDNRA	24326
EEA1	314764
EEF2K	25435
EFNA4	310643
EFNB3	360546
EGR2	114090
EGR3	25148
EHD1	293692
EIF5A	287444
ELAVL4	432358
EML1	362783
EPHA3	29210
EPHA4	316539
EPHB6	312275
ESR1	24890
ETV4	360635
ETV5	303828
EXOC5	60627
EZR	54319
F2R	25439
FAIM2	246274
FBXO31	498959
FEZ1	81730
FGF2	54250
FGF3	170633
FGF8	29349

FGF9	25444
FGF13	84488
FGFR1	79114
FIG4	309855
FKBP5	361810
FLOT1	64665
FLRT1	499308
FOXP2	500037
FST	24373
FZR1	314642
GABRG2	29709
GAL	29141
GAS7	85246
GBX1	246149
GDNF	25453
GEM	297902
GFRA1	25454
GLDN	315675
GLI2	304729
GPC2	171517
GPM6A	306439
GPR3	266769
GPR12	80840
GRASP	192254
GRIN2A	24409
GRIP1	84016
GRK5	59075
GRM1	24414
GRM6	24419
GRN	29143
GUCY1A3	497757
HCN1	84390
HMG20A	315689
HOMER1	29546
HOMER2	29547
HSPA1A/HSPA1B	294254
HTR7	65032
HTR1B	25075
IFNG	25712
IFT122	312651
IGF1	24482
IGSF9	304982
IL1RAP	25466

IL6R	24499
INHBA	29200
IRS2	29376
IRX6	307715
ITGA6	114517
ITM2C	301575
ITSN1	29491
JAK1	84598
JAK2	24514
JUNB	24517
KCNA1	24520
KCND2	65180
KDM1A	500569
KDM4A	313539
KITLG	60427
KL	83504
KLF9	117560
KRAS	24525
LCK	313050
LDB1	309447
LEF1	161452
LGI1	252892
LHX2	296706
LHX4	360858
LIMK2	29524
LMNA	60374
LMO4	362051
LPAR1	116744
LRRC4	641521
LRRN1	500280
LRRN3	81514
LRRTM3	294380
LRTM2	680883
LZTS1	266711
MAFK	246760
MAML3	310405
MAN2A1	25478
MAP2K3	303200
MAP2K4	287398
MAP2K5	29568
MAP2K6	114495
MAP3K12	25579
MAPK3	50689

MAPK15	286997
MAR8	312656
MCOLN3	308022
MFN1	192647
MINK1	303259
MMP3	171045
MUSK	81725
MYC	24577
MYT1L	116668
NAMPT	297508
NAPEPLD	296757
NCOR2	360801
NCSTN	289231
NECTIN1	192183
NEFL	83613
NEUROD1	29458
NEUROD2	54276
NEUROD4	288821
NFATC1	100361818
NFIB	29227
NGB	85382
NGF	310738
NOG	25495
NOTCH4	406162
NPTX1	266777
NPY1R	29358
NPY2R	66024
NR1D1	252917
NR2F1	81808
NRF1	312195
NRN1	83834
NTF3	81737
NTRK3	29613
NUAK1	299694
NYAP1	304376
OLIG2	304103
OXTR	25342
P2RX2	114115
PACSIN1	29704
PAK5	311450
PAK6	296078
PANX1	315435
PARD6A	307799

PBX1	304947
PCDH8	64865
PDGFB	24628
PDP1	54705
PECAM1	29583
PHACTR3	362284
PIEZO1	361430
PIK3C3	65052
PIK3CB	85243
PIK3CD	366508
PJA2	192256
PLAGL1	25157
PLAUR	50692
PLCL1	84587
PLPPR4	295401
PLXNA2	289392
PLXND1	312652
PMP22	24660
POLR3E	361640
POU4F2	171355
POU4F3	364855
PPP3CC	171378
PRAG1	306506
PRKAA1	65248
PRKAR2A	29699
PRKCQ	85420
PRKCZ	25522
PRSS12	85266
PRUNE1	310664
PSENEN	292788
PTGS2	29527
PTPDC1	291022
PTPRE	114767
PXN	360820
RAB11B	79434
RAB33A	317580
RAB3A	25531
RALGDS	29622
RAP1B	171337
RASA1	25676
RASGRF1	192213
REEP1	362384
REM2	64626

RET	24716
RGS2	84583
RGS14	114705
RHOQ	85428
RHOV	171581
RIT1	499652
RIT2	291713
RND1	362993
ROCK2	25537
RPS6KA3	501560
RPS6KB1	83840
RTN4RL1	303311
RTN4RL2	311169
RUNX1	50662
RXRB	361801
RXRG	83574
RYK	140585
SCN1A	81574
SCN1B	29686
SDC1	25216
SDCBP	83841
SDK2	360652
SEMA3A	29751
SEMA3D	246262
SEMA3E	296789
SH2D3C	362111
SHTN1	292139
SLC17A6	84487
SLC18A2	25549
SLC5A3	114507
SLC6A15	282712
SLITRK4	302473
SMAD3	25631
SMARCD3	296732
SNCA	29219
SOCS3	89829
SORBS2	114901
SOX4	364712
SPAST	362700
SPHK1	170897
SPRR1A	499660
SPRY2	306141
SS18	361295

SSTR3	171044
SUN1	360773
SV2A	117559
SYK	25155
TAC1	24806
TACC1	306562
TCTN1	304486
TGFB1	59086
TGFBR2	81810
TGIF1	316742
THRB	24831
TMEM106B	312132
TNFRSF12A	302965
TP73	362675
TPBG	83684
Tpm4	24852
TRIM9	155812
TRPC1	89821
TRPC6	89823
TWIST1	85489
TYRO3	25232
ULK2	303206
UST	361450
VASP	361517
VCL	305679
VEGFA	83785
VGF	29461
VWC2	501231
WNT6	316526
WNT7A	114850
XRCC4	309995
ZBTB18	64619

**Transcription factors group**	**Entrez ID**
ABTB1	297432
ANAPC2	296558
ANKRD5	296184
ANKRD6	500430
APBB1	29722
ARC	54323
ARHGEF25	314904
ARID5A	316327
ASCC1	294512

ASCC2	498402
ASCC3	309887
ATF1	315305
ATMIN	315037
BACH1	304127
BANP	292064
BAZ1A	314126
BBX	303970
BCL11A	305589
BCOR	317346
BHLHE22	365748
BHLHE23	499952
BHLHE40	79431
BTBD6	690367
CALCOCO1	246047
CARF	301446
CBFA2T3	361431
CBFB	361391
CBX8	303731
CCRN4L	310395
CDK5	140908
CHD1	308215
CHD6	311607
CHRAC1	315058
CHURC1	299154
CIR1	362149
CITED2	114490
CREBL2	362453
CREM	25620
CRIP2	338401
CRY1	299691
CSRNP1	363165
CSRP2	29317
CTDP1	291414
CUX2	288665
DBP	24309
DDX20	84473
DDX3X	317335
DEAF1	83632
DEPDC5	305464
DHX57	366532
DIP2C	307067
DOT1L	362831

DPF1	50545
DPF3	299186
EBF1	116543
EBF3	361668
EEA1	314764
EGR2	114090
EGR3	25148
EHF	295965
ELF3	304815
ELP2	307545
ESR1	24890
ETV4	360635
ETV5	303828
FBXO41	312504
FHL2	63839
FOXP2	500037
GAS7	85246
GBX1	246149
GLI2	304729
GRHL2	299979
GRM6	24419
GTF2IRD1	246770
GTF3C5	362095
GZF1	311508
HDAC11	297453
HES6	316626
HIC1	303310
HMG20A	315689
HMGA1	117062
HMGA2	84017
HP1BP3	313647
HPCAL1	50871
HSF2BP	499413
ING4	297597
IQSEC3	404781
IRF6	364081
IRX6	307715
JAK2	24514
JUNB	24517
KDM1A	500569
KDM4A	313539
KLF10	81813
KLF11	313994

KLF13	499171
KLF14	312203
KLF2	306330
KLF5	84410
KLF7	363243
KLF9	117560
KLHL14	364823
KLHL25	293023
KLHL36	498957
KLHL5	305351
LDB1	309447
LEF1	161452
LHX2	296706
LHX4	360858
LMO1///LMO3	245979
LMO2	362176
LMO4	362051
LZTFL1	316102
LZTS1	266711
MAF1	315093
MAFK	246760
MAML3	310405
MAPK3	50689
MCM5	291885
MED15	360743
MED4	306030
MEIS2	311311
MINK1	303259
MKRN1	296988
MLL5	311968
MLLT3	114510
MMS19	171124
MNT	287521
MRRF	311903
MTERFD3	366856
MXD4	360961
MXI1	25701
MYC	24577
MYCL1	298506
MYT1L	116668
NAB2	314910
NAP1L3	170914
NCALD	553106

NCOR2	360801
NEUROD1	29458
NEUROD2	54276
NEUROD4	288821
NFATC1	100361818
NFIB	29227
NFKBIE	316241
NFRKB	315523
NFX1	313166
NHLH2	295327
NOC3L	361753
NOP2	314969
NOTCH4	406162
NR1D1	252917
NR1D2	259241
NR1I3	65035
NR2F1	81808
NRF1	312195
NRIP1	304157
NRIP3	361625
OLIG2	304103
PAPOLA	314417
PAX1	311505
PBX1	304947
PCBP4	363133
PCGF2	287662
PEX14	64460
PHF14	500030
PHF20	311575
PITX1	113983
PKNOX1	294322
PKNOX2	680549
PLAGL1	25157
PLXNA2	289392
PLXND1	312652
PNRC1	286988
POLE4	362385
POLR1A	83581
POLR3E	361640
POU4F2	171355
POU4F3	364855
POU6F1	116545
PPRC1	294007

PTBP1	29497
PURG	361162
RAD21	314949
RAI14	294804
RBAK	288489
RBPMS	498642
RFXAP	499617
RGD1563666	317617
RGS14	114705
ROCK2	25537
RPS6KA5	314384
RUNX1	50662
RUNX1T1	362489
RUNX2	367218
RXRB	361801
RXRG	83574
RYBP	312603
SAP30BP	360662
SATB1	316164
SCRT2	366229
SERTAD1	361526
SFMBT1	58967
SIM1	309888
SIN3A	363067
SIRT3	293615
SIRT5	306840
SMAD3	25631
SMARCA2	361745
SMARCD3	296732
SMYD5	312503
SORBS2	114901
SOX4	364712
SP7	300260
SRSF9	288701
ST18	266680
TADA2B	289717
TAF12	682902
TBX21	303496
TCEAL1	302593
TEAD1	361630
TEF	29362
TFAP4	360482
TGFB1	59086

TGIF1	316742
THRB	24831
TOX3	291908
TP73	362675
TRERF1	316219
TRIM23	81002
TRIM9	155812
TSC22D1	498545
TWIST1	85489
TXNIP	117514
USF1	83586
VGLL4	297523
VPS25	681059
WDHD1	305827
WDR45L	360682
ZBTB16	353227
ZBTB22	309630
ZBTB46	311718
ZBTB5	298084
ZBTB9	294289
ZC3H18	292067
ZC3H8	311414
ZFA///ZFAT	362925
ZFAND6	293067
ZFP62	361500
ZFPM1	691504
ZGPAT	296478
ZMIZ1	361103
ZMYND8	296374
ZNF217	311764
ZNF238	64619
ZNF358	360754
ZNF395	305972
ZNF414	299647
ZNF467	500110
ZNF513	313913
ZNF575	308430
ZNF672	303165
ZNF697	295310
ZNF709///ZNF14	266773
ZNF775	312309
ZNF786	100158223
ZNF90///ZFP90	498945

ZYX	114636

**Epilepsy Group**	**Entrez ID**
ABHD6	305795
ADM	25026
ADRA1A	29412
ADRA1B	24173
ADRA1D	29413
ANKRD6	500430
ARC	54323
BAG3	293524
BAIAP2	117542
BBC3	317673
BDNF	24225
BEND5	362564
CACNB4	58942
CALCRL	25029
CAMKK1	60341
CH25H	309527
CITED2	114490
CNTN2	25356
CREM	25620
CRYM	117024
CXCR4	60628
CYR61	83476
DBP	24309
DEPDC5	305464
DNAJB5	313811
DUSP5	171109
DUSP6	116663
EGR2	114090
EGR3	25148
ERRFI1	313729
FGF13	84488
GABRD	29689
GABRG2	29709
GABRQ	65187
GADD45B	299626
GAL	29141
GFRA1	25454
GRASP	192254
GRIN2A	24409
HAS1	282821

HCN1	84390
HOMER1	29546
HSPA1A/HSPA1B	294254
HTR1B	25075
HTR1F	60448
HTR7	65032
IFRD1	29596
INHBA	29200
JUNB	24517
KCNA1	24520
KCNB1	25736
KCNK1	59324
KCNV1	60326
KLF10	81813
LAMB3	305078
LAMP5	362220
LGI1	252892
MCL1	60430
METRNL	316842
NAMPT	297508
NGF	310738
NPTX2	288475
NTF3	81737
PCDH19	317183
PDP1	54705
PLK2	83722
PNPO	64533
POLG	85472
PRICKLE1	315259
PRRT2	361651
PTGS2	29527
RGS16	360857
RGS2	84583
SCG2	24765
SCN1A	81574
SCN1B	29686
SCN2A	24766
SCN3B	245956
SCN4A	25722
SERTAD1	361526
SLC2A3	25551
SOCS3	89829
SPNS2	100270678
SPRY2	306141

SSTR2	54305
ST3GAL3	64445
STRADA	303605
SV2A	117559
SV2C	29643
TAC1	24806
TEF	29362
TRIB1	78969
TRIM9	155812
TRPC7	282822
TWNK	309441
TYRO3	25232

Neuroinflammation list of WP+B and RX2+B reversal genes

Symbol	Log2 Fold Change
KL	-3.603
MMP3	-3.374
GRIN2A	-3.138
IL6R	-2.765
TGFBR2	-2.452
PTGS2	-2.32
FGFR1	-2.314
IFNG	-2.047
ACVR1C	-2.02
KCNJ5	-1.993
GDNF	-1.869
NGF	-1.851
PLA2G4A	-1.756
IRS2	-1.745
BDNF	-1.537
NFATC1	-1.438
JAK2	-1.08
CFLAR	-1.05
TGFB1	-0.946
GABRQ	-0.916
ACVR1	-0.898
PIK3CB	-0.897
MAP2K4	-0.896
AKT2	-0.726
CX3CL1	-0.675
JAK1	-0.662
SYK	-0.526
NCSTN	0.357
FZD1	0.792
PPP3CC	0.793
IFNGR2	0.802
PIK3C3	1.028
GABRG2	1.162
CD200	1.176
MAPK3	1.215
SNCA	1.292
PSENEN	1.339
GABRD	1.796
PIK3CD	2.002
IL34	2.079
MAPK15	2.542
